# Pathogenic and Non-Pathogenic Microorganisms in the Rapid Alert System for Food and Feed

**DOI:** 10.3390/ijerph16030477

**Published:** 2019-02-06

**Authors:** Marcin Pigłowski

**Affiliations:** Department of Commodity and Quality Management, Faculty of Entrepreneurship and Quality Science, Gdynia Maritime University, Morska 81-87 Str., 81-225 Gdynia, Poland; m.piglowski@wpit.umg.edu.pl; Tel.: +48-58-558-6295

**Keywords:** food safety, pathogens, European Union, RASFF, pivot tables, cluster analysis

## Abstract

The most frequently notified pathogenic microorganisms in the RASFF in 1980–2017 were *Salmonella* sp., *Listeria*, *Escherichia* and *Vibrio*, whereas, among the notified non-pathogenic microorganisms were unspecified microorganisms, Enterobacteriaceae, *Salmonella* sp. and Coliforms. Microorganisms were reported mainly in poultry meat, meat, fish, molluscs, crustaceans, fruits, vegetables, herbs, spices, nuts, milk, cereals (in food) and in feed materials and pet food (in feed). The number of notifications decreased at the turn of 2005 and 2006, but has steadily increased since then. The notification basis were official controls, border controls and company’s checks. Products were notified mainly by Italy, France, United Kingdom, Germany and Netherlands. The reported products originated from Brazil, European Union countries and India, Thailand and Vietnam. The notification types were alerts, information and border rejections. The distribution status was often not specified or distribution on the market was possible. The risk decision was usually not made. Products were re-dispatched, import was not authorised or products were withdrawn from the market, destroyed and recalled from the market. Proper cooperation within the framework of the RASFF can contribute to shaping public health law and reducing outbreaks associated with microorganisms.

## 1. Introduction

Microorganisms and substances produced by them can be present and can grow in food and feed of animal and non-animal origin. They can cause serious diseases risk to people and animals, threatening their health and life [1]. A pathogenic organism is an organism which is capable of causing diseases in a host (person) [2]. The World Health Organization (WHO) listed among hazards that may be present in food potentially harmful bacteria, viruses, toxins, parasites and chemicals. According to the WHO, one person out of 10 people falls ill and 420,000 people die each year as a result of eating contaminated food [1]. Foodborne diseases caused by pathogenic bacteria can be, e.g., salmonellosis, listeriosis, campylobacteriosis and yersiniosis [3]. Organisms, which do not cause diseases are called non-pathogenic [2].

In 1980–2017 notifications on pathogenic microorganisms (bacteria, viruses and toxins that they produced) were about 19% of all notifications in the Rapid Alert System for Food and Feed (RASFF) and were preceded only by mycotoxins (21.3%) [4]. However, according to Parisi et al., in periods 1979–1990 and 2011–2014 just pathogenic microorganisms were most frequently notified [5]. Yet, also non-pathogenic microorganisms were notified in the RASFF (about 2.5% of notifications) [4].

The RASFF was created already in 1979; however, its current legal basis is Regulation (EC) No 178/2002 laying down the general principles and requirements of food law, establishing the European Food Safety Authority (EFSA) and laying down procedures in matters of food safety [6,7]. The implementing measures for the RASFF were laid down in Regulation (EU) No 16/2011 [8]. The RASFF enables exchanging information between its members and reacting when risks to public health in the food and feed chain are detected. The RASSF members are 28 national food authorities of the European Union (EU) countries, European Commission, EFSA, European Free Trade Association Surveillance Authority (ESA), Norway, Liechtenstein, Iceland and Switzerland. The information exchange helps RASFF members to act rapidly and in a coordinated way in response to health hazard in food or feed. The food or feed product can be inspected on the market or at the border, including also laboratory examination. If the product does not meet the requirements it can be reported within the national system. The authority decides if this product should be reported to the national RASFF contact point. This point verifies and completes the notification and submits it to the European Commission. The Commission is responsible for the RASFF management and it checks notifications before making them available to all the network members. The European Commission also informs the country, from which the notified product comes or to which it is to be exported [7].

In the RASFF alert notifications (the notification type sent already since 1979) are used when food or feed presenting a serious risk is on the market and rapid action is required (e.g., product withdrawal). Information notifications (sent since 1989) are used when a risk in food or feed has been identified, but rapid action is not needed. Information notifications are divided into information for follow-up (since 2010) and information for attention (since 2011). Border rejections (sent since 2008) are used when food or feed consignments have been tested and rejected at the EU (or the European Economic Area) external border post [7].

Research including notifications related to microorganisms in the RASFF can be used by European and national institutions to ensure food and feed safety (eliminating or limiting the spread of hazards) and to improve the RASFF. Data on the most frequently notified hazards can be useful for business operators, who can take appropriate preventive actions and thus shape their image and avoid economic losses. In turn, consumers can enhance their awareness of possible hazards. However, only in RASFF annual reports for 2016 and 2017 notifications on microorganisms were widely discussed, yet this discussion related generally only to the year in question. In previous annual reports these hazards were discussed only in a general way. Moreover, each annual RASFF report was released only in the middle of the next year (or even later), so many months after the year it concerned. Therefore, it is necessary to analyse in detail the data from the RASFF database and take into consideration the largest possible number of variables available. The goal of the study was to examine similarities in notifications regarding pathogenic and non-pathogenic microorganisms in food and feed taking into account: year, product category, notifying country, origin country, notification basis, notification type, distribution status, risk decision and action taken.

## 2. Materials and Methods

### 2.1. Data and Methods Used

The data originated from the RASFF database from 1980–2017 and concerned 11,031 notifications. They related to two hazard categories: pathogenic microorganisms (9729 notifications) and non-pathogenic microorganisms (1302 notifications). The data was transferred to Excel and ordered in pivot tables, separately for different product type (food and feed). The data collected in tables included microorganisms and the following variables: year, product category, notifying country, origin country, notification basis, notification type, distribution status, risk decision and action taken.

The data gathered in pivot tables was next transferred to Statistica 12 (Tulsa, OK, USA), where the cluster analysis based on each table was carried out. The purpose of this analysis was to identify similarities in notifications of microorganisms within particular variables. There were two methods of the cluster analysis used: first joining (tree clustering) and then two-way joining.

In the case of joining the Euclidean distance as the distance measure was adopted and a Ward’s method as a linkage rule. The results were presented in tree diagrams. The Euclidean distance is a geometric distance in the multidimensional space and it is the most commonly used distance measure. The Ward’s method uses a variance analysis to evaluate a distance between clusters, attempting to minimize the sum of squares of deviations within clusters. This method is considered to be very efficient, but it tends to form clusters of small size. The use of joining was intended to examine notifications, in which microorganisms were similar, considering particular variables mentioned above.

In contrast, in the case of two-way joining figures were presented with contours, which changed colours (from green, through yellow, orange, red to brown) and thickened in the clusters. The two-way joining was used to uncover which notifications created clusters (i.e., which notifications were similar) if we took into consideration simultaneously: microorganisms and values of other, particular variables.

### 2.2. Remarks on Processing of Some Data

If the microorganism name in the RASFF database was not given, the phrase “unspecified” was adopted. Similarly, if the data related to the notification basis, distribution status and action taken was not given, the phrase “not specified” or “(not specified)” was used. In the early years of the RASFF, species of microorganisms were not reported in notifications. In later years, in turn, species were often very diverse. Therefore, in the case of *Salmonella* the phrase “*Salmonella* sp.” was used and in other cases only the microorganism genus was adopted. If notification related to more than one microorganism or more than one origin country, only the first microorganism or the first origin country from the mentioned ones was adopted. All these actions were necessary to prepare the data for statistical calculations.

In order to make figures more readable, the names of some values were shortened and the full names were given below the figures. Moreover, before the two-way joining cluster analysis the number of microorganisms and values of other variables were limited to twenty most often notified. The disadvantage of the two-way joining was on the one hand—reducing clusters and excluding brown and red colour (if the data was more scattered) and on the other hand—quenching clusters with a smaller number of notifications.

## 3. Results

### 3.1. Pathogenic Microorganisms

Notifications on pathogenic microorganisms in the RASFF, considering all product types (food, feed and food contact material), were about 19% of all notifications (9729 notifications) in 1980–2017. The vast majority were notifications on food (8322; 85.5%), there were also 1406 notifications on feed (14.5%) and one notification on food contact material. Notifications concerned mainly bacteria and viruses (in very rare cases their presence was only suspected). In particular, notifications concerned: *Salmonella* sp. (6202 notifications; 63.7% of all notifications), *Listeria* (1390; 14.3%), *Escherichia* (859; 8.8%), *Vibrio* (341; 3.5%), Norovirus (226; 2.3%), *Bacillus* (185; 1.9%), *Campylobacter* (153; 1.6%) and also *Clostridium*, unspecified microorganisms, *Staphylococcus*, *Hepatitis A*, *Pseudomonas*, Coliforms, *Cronobacter*, *Hyphomycetes*, *Shigella*, *Brucella*, *Yersinia*, *Calicivirus*, *Mycobacterium* and others (below 1%).

The results of the joining cluster analysis taking into account particular variables were presented in Supplementary Materials, in Figures S1–S9 for food and in Figures S10–S18 for feed. Within notifications on food, notifications on *Salmonella* sp. created a one-element cluster in the case of each variable. However, similar character had notifications on *Listeria* and *Escherichia*, which formed a two-element cluster in the case of year (Figure S1), product category (Figure S2), notifying country (Figure S3), origin country (Figure S4), notification basis (Figure S5) and risk decision (Figure S8). Whereas, notifications on *Listeria*, *Escherichia* and *Vibrio* created a cluster in the case of distribution status (Figure S7) and action taken (Figure S9). Notifications within feed related mainly to *Salmonella* sp. and they created a one-element cluster in the case of each considered variable (Figures S10–S18).

Notifications on microorganisms mentioned above exceeded the mean value. In the case of food these were: *Salmonella* sp. (4809 notifications; 57.8%), *Listeria* (1388; 16.7%), *Escherichia* (857; 10.3%) and *Vibrio* (341; 4.1%). In the case of feed it was only *Salmonella* sp. (1406; about 99%). Figure 1 presents the numbers of notifications on these pathogenic microorganisms taking into account particular variables. The results are supported by the two-way joining cluster analysis (Supplementary Materials, Figures S19–S27 for food and Figures S28–S36 for feed).

#### 3.1.1. Pathogenic Microorganisms in Food

**Figure 1:**
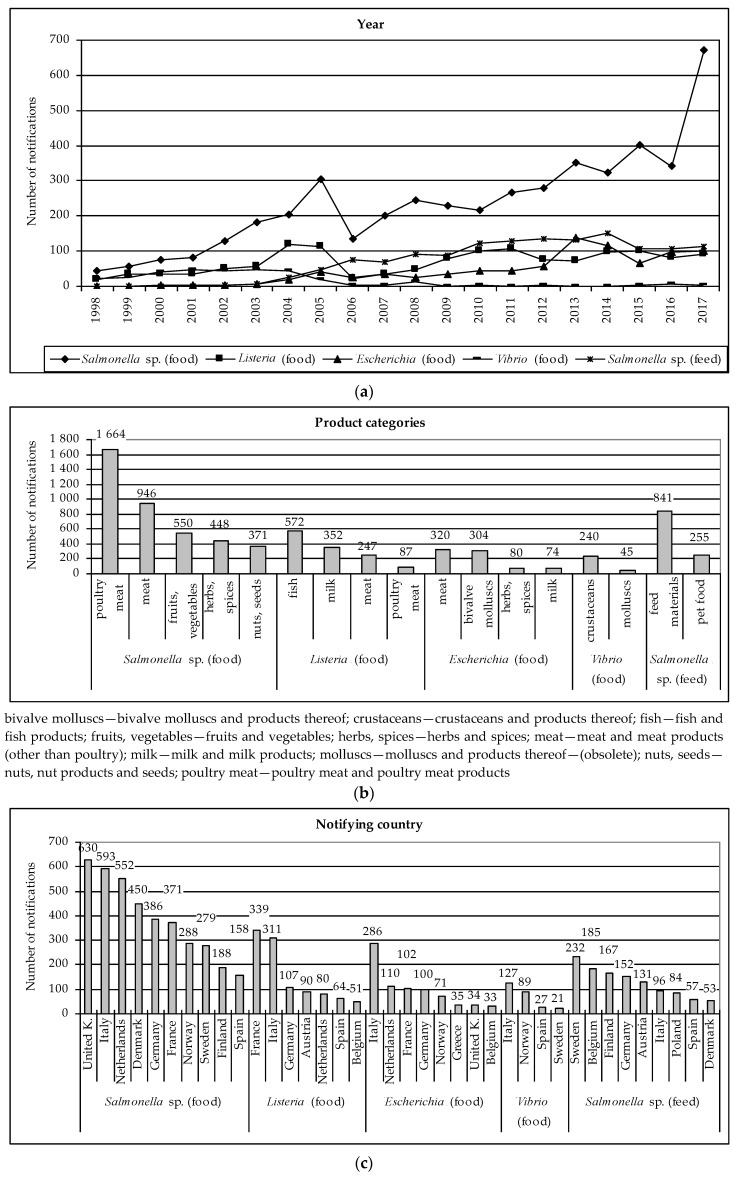

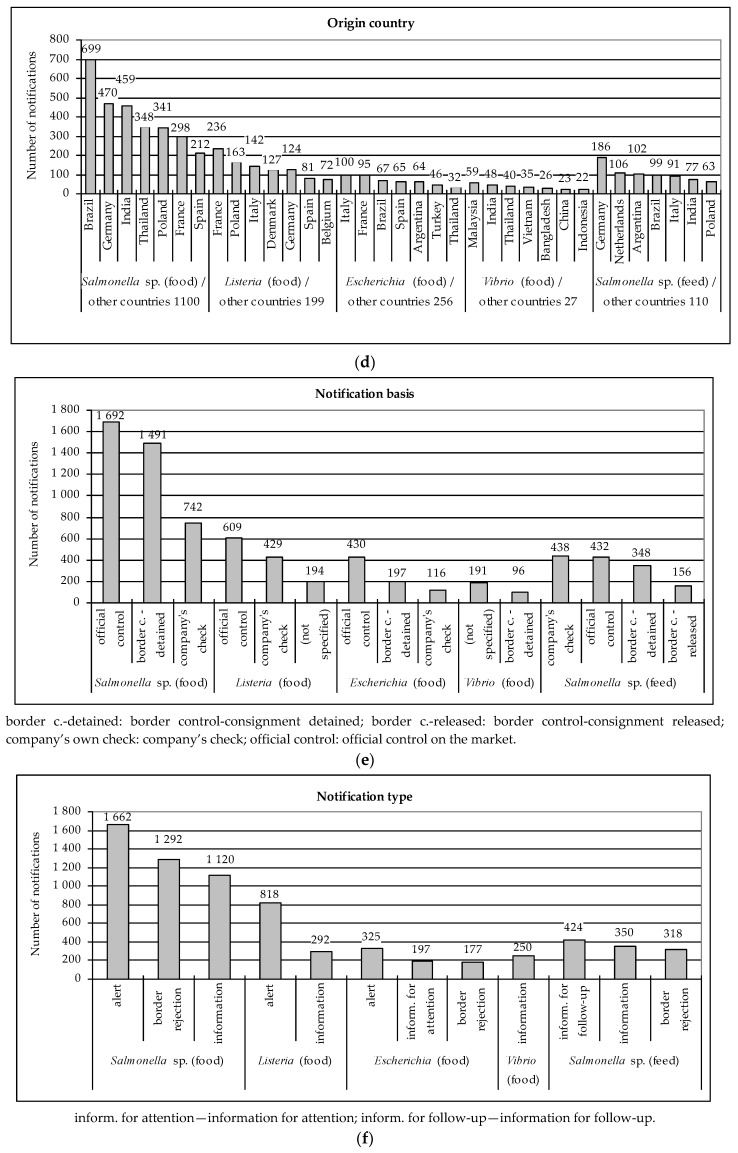

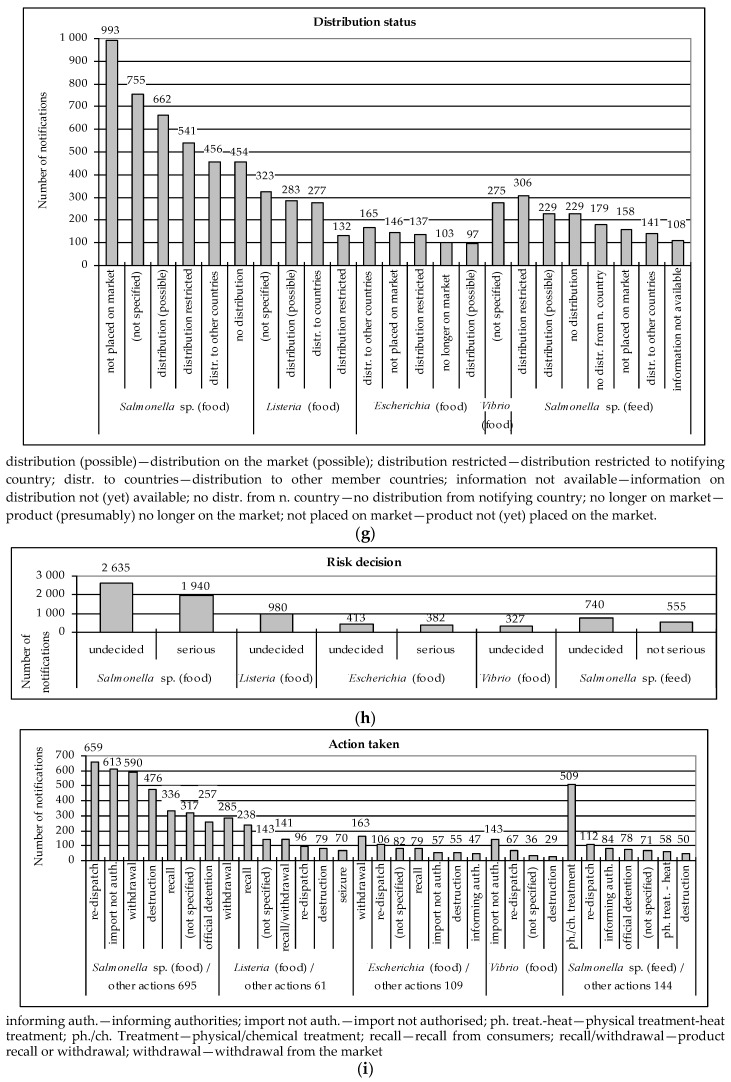
The numbers of RASFF notifications of pathogenic microorganisms in food and feed: (**a**) Year; (**b**) Product categories; (**c**) Notifying country; (**d**) Origin country; (**e**) Notification basis; (**f**) Notification type; (**g**) Distribution status; (**h**) Risk decision; (**i**) Action taken. Source: own study on the basis of [4].

Values of variables concerning origin country and action taken were most diverse, particularly in the case of *Salmonella* sp. The number of notifications on *Salmonella* sp. in food increased from 1998 to 2005. After a decrease in 2006 one can observe a mild increase in the number of these notifications and then a sharp increase in 2017 (Figure 1a). The highest number of notifications on *Salmonella* sp., *Listeria* and *Escherichia* was in 2015–2017 (Figure S19). Whereas, the highest numbers of notifications on *Vibrio* occurred in 2003 and 2004. The most frequent notifications concerned poultry meat (*Salmonella* sp. and *Listeria*), meat (*Salmonella* sp., *Listeria* and *Escherichia*) or aquaculture products, i.e., fish (*Listeria*), bivalve molluscs (*Escherichia*) and crustaceans (*Vibrio*). Notifications concerned also fruits, vegetables (*Salmonella* sp.), herbs, spices (*Salmonella* sp. and *Escherichia*), nuts (*Salmonella* sp.) and milk (*Listeria* and *Escherichia*) (Figure 1b, Figure S20).

Products with pathogenic microorganisms were notified mainly by Italy, then by France, Netherlands, Germany, United Kingdom (Figure 1c), Denmark and Norway (Figure S21). The largest number of notifications concerned products originating from Brazil. The notified products originated also from EU countries, i.e., Germany, France, Italy, Spain, Netherlands and Poland. Furthermore, notifications concerned products from Far Eastern countries, i.e., India, Thailand and Vietnam (Figure 1d, Figure S22). The notification basis was usually official control and border control, after which the consignment was detained, and company’s own check (Figure 1e, Figure S23). The most common notification type was alert, then information and border rejection (Figure 1f, Figure S24). Distribution status was most often not specified. Distribution on the market could be possible, restricted to the notifying country or the product was not (yet) placed on the market (Figure 1g, Figure S25). In most notifications the risk decision was not made or risk was serious (Figure 1h, Figure S26). Products with pathogenic microorganisms were usually withdrawn from the market and re-dispatched (Figure 1i, Figure S27).

#### 3.1.2. Pathogenic Microorganisms in Feed

The number of notifications on *Salmonella* sp. in feed in the RASFF increased from 2002 to 2014 and afterwards decreased and stabilisation occurred in 2015–2017 (Figure 1a). The number of notifications above the mean value occurred in 2008 and then in 2010–2017 (Figure S28). The notifications concerned mainly feed materials and pet food (Figure 1b, Figure S29). They were reported by Sweden, Belgium, Finland, Germany and Austria (Figure 1c, Figure S30). The notified products originated from Germany, then from Netherlands, Argentina, Brazil and Italy (Figure 1d, Figure S31). The notified basis was mainly company’s own check and official control, then border control, after which the consignment was detained or released (Figure 1e, Figure S32). The notification type was information for follow-up, information or border rejection (Figure 1f, Figure S33). Distribution was restricted to the notifying country, distribution on the market was possible or products were not distributed (Figure 1g, Figure S34). The risk decision was not made or risk was not serious (Figure 1h, Figure S35). The notified products were usually physically or chemically treated and also re-dispatched (Figure 1i, Figure S36).

### 3.2. Non-Pathogenic Microorganisms

Notifications on non-pathogenic microorganisms, considering all product types, were over 2.5% (1302 notifications) of all the notifications in the RASFF in 1980–2017. The majority were notifications on food (1077; 82.7%), on feed there were 224 notifications (17.2%) and only one notification related to food contact material. The name of the non-pathogenic microorganism was usually not indicated or was too general (e.g., mesophiles) and it related to as many as 768 notifications (about 59%). In these cases the phrase “unspecified” was adopted. However, in the description of this kind of notifications very often moulds were given as a hazard or in rare cases—yeasts. The other notifications were related to: Enterobacteriaceae (277; 21.3%), *Salmonella* sp. (99; 7.6%), Coliforms (68; 5.2%), *Bacillus* (19; 1.5%), *Streptococcus* (19; 1.5%) and also *Escherichia*, *Listeria*, *Staphylococcus*, *Hyphomycetes*, *Penicillium*, *Pseudomonas*, *Aspergillus*, *Fusarium*, *Clostridium*, *Vibrio*, *Aureobasidium*, *Campylobacter* and *Enterococcus* (below 1%).

The results of the cluster analysis using joining and taking into account individual variables were presented in Supplementary Materials, in Figures S37–S45 for food and in Figures S46–S54 for feed. Notifications on food had similar character in the case of unspecified microorganisms and Enterobacteriaceae, and also *Salmonella* sp. and Coliforms. They formed two separate two-element clusters in the case of year (Figure S37), product category (Figure S38), notification basis (Figure S41), notification type (Figure S42) and risk decision (Figure S44). The cluster consisting of unspecified microorganisms and Enterobacteriaceae was formed also in the case of notifying country (Figure S39) and action taken (Figure S45). Two one-element clusters (concerned notifications on unspecified microorganisms and Enterobacteriaceae, respectively) were created in the case of origin country (Figure S40). Whereas, notifications on *Salmonella* sp., Coliforms and *Streptococcus* had also similar character in the case of notifying country (Figure S39), origin country (Figure S40) and distribution status (Figure S43). Within notifications on feed mainly unspecified microorganisms were reported and they created a one-element cluster in the case of all the considered variables (Figures S46–S54). Notifications on Enterobacteriaceae, *Salmonella* sp. and *Bacillus* were similar in the case of year (Figure S46) and product category (Figure S47). In turn, notifications on Enterobacteriaceae, Coliforms, *Salmonella* sp. and *Bacillus* had similar character in the case of distribution status (Figure S52) and risk decision (Figure S53). However, notifications on Enterobacteriaceae and *Salmonella* sp. were similar in the case of action taken (Figure S54).

The mean value was exceeded by notifications on: unspecified microorganisms (597 notifications; 55.4%), Enterobacteriaceae (258; 24.0%), *Salmonella* sp. (86; 8.0%) and Coliforms (61; 5.7%) in food and only unspecified microorganisms (170; 75.9%) in feed. The numbers of notifications on these microorganisms taking into account particular variables were presented in Figure 2. These results were supported by the two-way joining cluster analysis (Supplementary Materials, Figures S55–S63 for food and Figures S64–S72 for feed).

#### 3.2.1. Non-Pathogenic Microorganisms in Food

**Figure 2:**
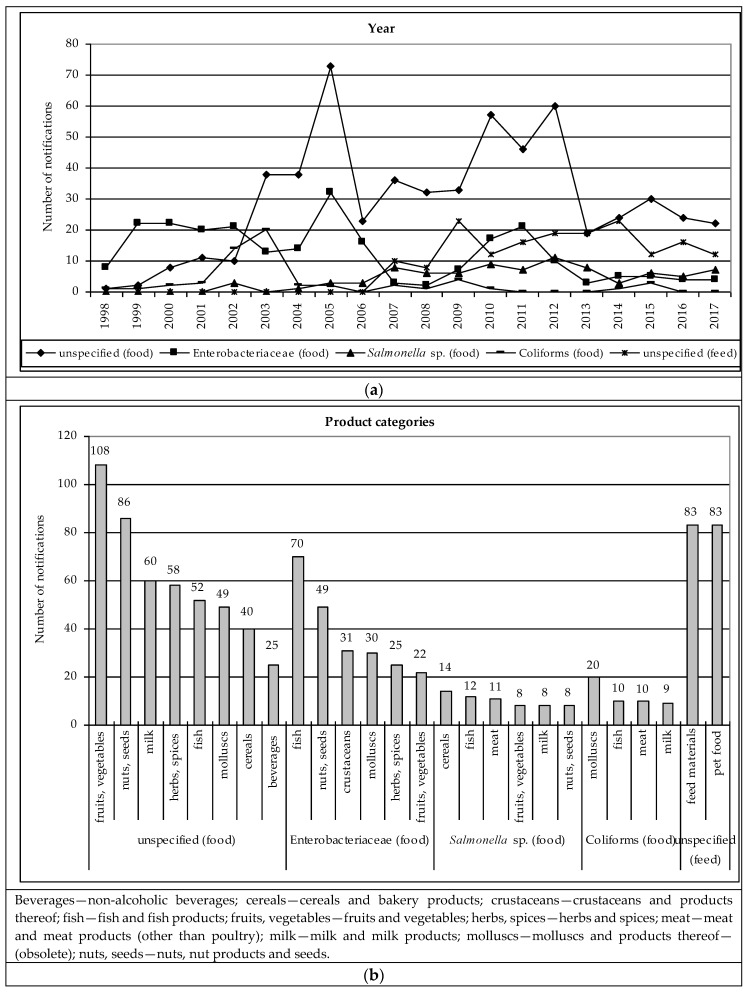

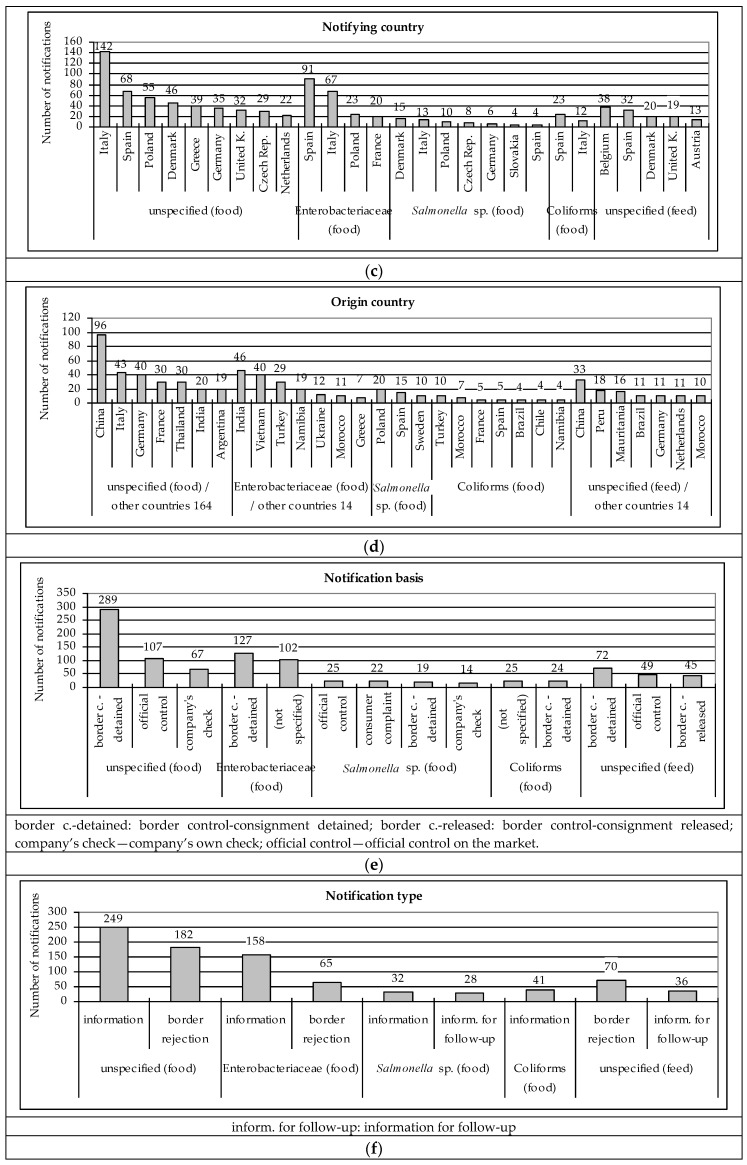

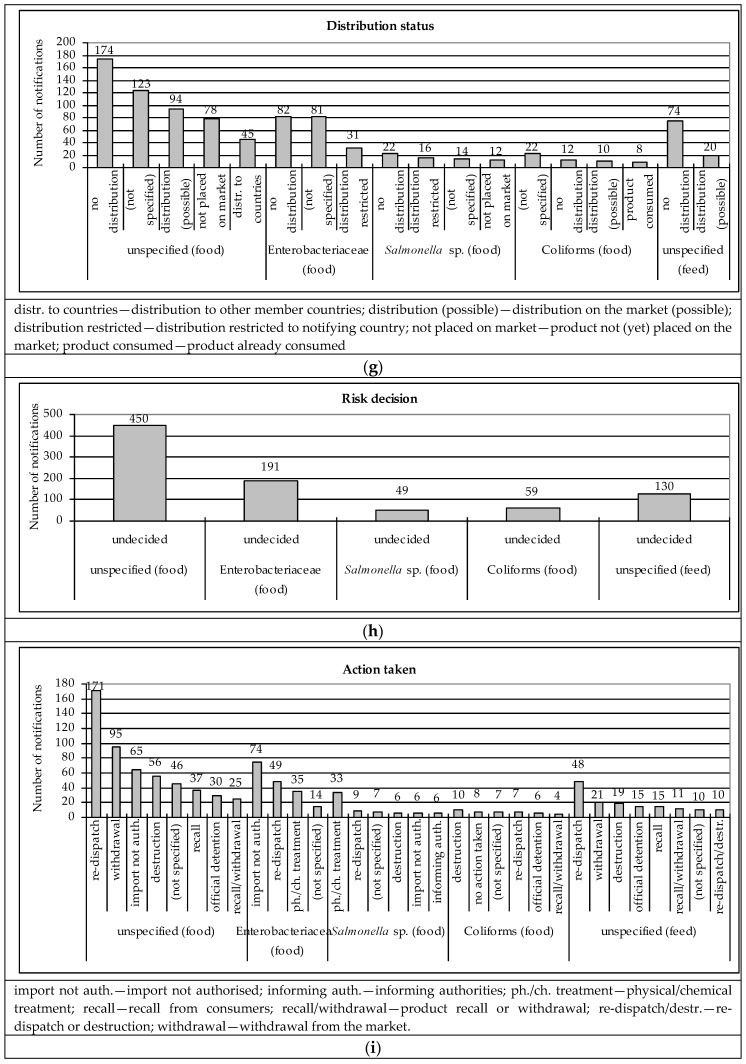
The numbers of RASFF notifications of non-pathogenic microorganisms in food and feed: (**a**) Year; (**b**) Product categories; (**c**) Notifying country; (**d**) Origin country; (**e**) Notification basis; (**f**) Notification type; (**g**) Distribution status; (**h**) Risk decision; (**i**) Action taken. Source: own study on the basis of [4].

The number of notifications of non-pathogenic microorganisms in food decreased after 2005. It can be particularly observed in the case of unspecified microorganisms and Enterobacteriaceae, but also *Salmonella* sp. Then, an increase continued to 2012, after which a decrease could be observed again and in the last years (2013–2017) the number of notifications stabilised (Figure 2a, Figure S55). The highest numbers of notifications on Coliforms occurred in 2002 and 2003. Notifications on fruits and vegetables were most frequently reported and related to unspecified microorganisms, Enterobacteriaceae and *Salmonella* sp. These microorganisms were also notified in nuts and seeds. In fish unspecified microorganisms, Enterobacteriaceae, *Salmonella* sp. and Coliforms were notified. In other aquaculture products, like molluscs, unspecified microorganisms, Enterobacteriaceae and Coliforms were notified and in crustaceans—Enterobacteriaceae. Notifications concerned also herbs and spices (unspecified microorganisms and Enterobacteriaceae), milk (unspecified microorganisms, *Salmonella* sp. and Coliforms), cereals (unspecified microorganisms and *Salmonella* sp.) and meat (*Salmonella* sp. and Coliforms) (Figure 2b, Figure S56).

The products were notified mainly by Italy and Spain, then by Poland, Denmark, Greece, Germany, United Kingdom, Czech Republic and France (Figure 2c, Figure S57). Products with non-pathogenic microorganisms originated mainly from Asian countries, i.e., China, India, Turkey, Vietnam and Thailand, and from EU countries, i.e., Italy, Germany and France and Poland and Spain (Figure 2d, Figure S22). The notification basis were usually: border control, after which the consignment was detained or official control; however, it could be also not specified (Figure 1e, Figure S59). The notification type were mainly information and border rejection (Figure 1f, Figure S60). Products were usually not distributed or their distribution status was not specified (Figure 2g, Figure S61). The risk decision was mainly not made (Figure 2h, Figure S62). The notified products were re-dispatched, withdrawn from the market or import was not authorised (Figure 2i, Figure S63).

#### 3.2.2. Non-Pathogenic Microorganisms in Feed

In the case of notifications of non-pathogenic microorganisms in feed only notifications on unspecified microorganisms exceeded the mean value. The greatest number of notifications occurred in 2009 and 2014 (Figure 2a, Figure S64). The notifications related to feed materials and pet food (Figure 2b, Figure S65). These products were notified mainly by Belgium and Spain (Figure 2c, Figure S66) and originated from China and Peru (Figure 2d, Figure S67). The notification basis were border control, after which the consignment was detained or released, then official control on the market (Figure 2e, Figure S68). Therefore, the notification type was mainly border rejection, but also information for follow-up (Figure 2f, Figure S69). Products were usually not distributed, however, distribution on the market was also possible (Figure 2g, Figure S70). The risk decision was usually not made (Figure 2h, Figure S35). The notified products were mainly re-dispatched, destroyed or withdrawn from the market (Figure 2i, Figure S36).

## 4. Discussion

### 4.1. Legal Conditions for Notifications of Microorganisms in the RASFF

The basis for food safety in the European Union is Regulation (EC) No 178/2002 laying down the general principles and requirements of food law (in force from 2002 and 2005). However, both in the case of pathogenic (particularly *Salmonella* sp. and *Listeria*) and non-pathogenic microorganisms a significant increase in the number of notifications can be observed at the turn of 2004 and 2005 (Figure 1a and Figure 2a, respectively). This can be connected with two law acts that came into force in 2003: Regulation (EC) No 2160/2003 on the control of *Salmonella* and other specified food-born zoonotic agents [9] and Directive 2003/99/EC on the monitoring of zoonoses and zoonotic agents [10]. The purpose of Regulation (EC) No 2160/2003 was to ensure measures to detect and to control *Salmonella* sp. and other zoonotic agents at all stages of production (particularly primary production), processing and distribution in order to reduce their prevalence and the risk to public health. It required designating a competent authority in each country and establishing relevant community targets, control programmes and methods [9]. The purpose of Directive 2003/99/EC was to ensure that zoonoses and zoonotic agents are monitored and food-borne outbreaks are investigated to evaluate relevant trends and sources [10]. Thus, these two legal acts obliged EU countries to create a system for tracking hazards related to microorganisms, which initially contributed to an increasing number of notifications.

In turn, in 2004 and 2006 hygiene package came into force. It consisted of: Regulation (EC) No 852/2004 on the hygiene of foodstuffs [11], Regulation (EC) No 853/2004 laying down specific hygiene rules for food of animal origin [12] and Regulation (EC) No 854/2004 laying down specific rules for organising official controls of products of animal origin intended for human consumption [13]. The official controls on the market were most often the notification basis in the case of pathogenic microorganisms (Figure 1e). However, they were also the basis for notifying non-pathogenic microorganisms (Figure 2e). The results of official controls were then alerts or information notifications (Figure 1f and Figure 2f).

Regulation (EC) No 854/2004 was amended by Regulation (EC) No 882/2004 on official controls performed to ensure verification in compliance with feed and food law, animal health and animal welfare rules [14]. In 2005 and 2006 Regulation (EC) No 183/2005 laying down requirements for feed hygiene came into force [15]. The important fact was that in 2004 ten countries joined the European Union. This resulted in an increase in trade in the field of food and feed within the enlarged EU, as well as between EU and non-EU countries. At the same time the supervisory authorities from newly accessed countries were fully included in the RASFF.

In 2006 Regulation (EC) No 2073/2005 laying down microbiological criteria for foodstuffs came into force and in this year an important decrease in RASFF notifications could be observed. This regulation obliged food operators to ensure that foodstuff meets relevant microbiological criteria and to take measures based on the Hazard Analysis Critical Control Point (HACCP) principles at each stage of food production, processing and distribution, including retail. In the annex to this Regulation food safety criteria for *Salmonella* sp., *Listeria monocytogenes* and *Escherichia coli* for various food categories were given. The annex included also process hygiene criteria for meat and products thereof (*Salmonella* sp., *Escherichia coli*, Enterobacteriaceae), milk and dairy products (*Escherichia coli*, Enterobacteriaceae), egg products (Enterobacteriaceae), fishery products (*Escherichia coli*) and vegetables, fruits and products thereof (*Escherichia coli*). In the European law, attention was therefore drawn to the most frequently notified product categories (Figure 1b and Figure 2b). When the results of testing were unsatisfactory, food operators were obliged to take relevant measures, e.g., withdrawal, recall, further processing by treatment eliminating the hazard in question. These actions were most frequently taken with regard to notified products (Figure 1i and Figure 2i). Food operators were also obliged to analyse trends in the test results and take the appropriate actions to prevent occurrence of microbiological risks [16].

In 2009 and 2010 Regulation (EC) No 669/2009 came into force. It implemented Regulation (EC) No 882/2004 (related to hygiene package) as regards the increased level of official controls on imports of certain feed and food of non-animal origin [17]. Border controls were often the notification basis (Figure 1e and Figure 2e) and they resulted in border rejections or information notifications (Figure 1f and Figure 2f). In turn, in 2011 Regulation (EU) No 16/2011 came into force. It laid down the implementing measures for the RASFF. In these years, a further increase in the number of notifications could be observed, but it was more related to non-pathogenic microorganisms. Currently, further changes in law are introduced, and Regulations No 854/2004 and No 882/2004 will be repealed by Regulation (EU) 2017/625 on official controls and other official activities performed to ensure the application of food and feed law, rules on animal health and welfare, plant health and plant protection products (date of effect: 14.12.2019) [18].

### 4.2. Microorganisms in the RASFF and Other Warning Systems Reports

In RASFF annual reports prevalence of pathogenic microorganisms in individual products was observed only from 2005. Usually, these reports indicated presence of *Salmonella* sp. in poultry meat, meat, fruits, vegetables, herbs and spices. *Salmonella* sp. was also indicted in feed materials and pet food. This data confirms a dominant share of *Salmonella* sp. in RASFF notifications (Figure 1b). The annual reports mentioned also *Listeria monocytogenes* in milk, meat and fish, *Escherichia coli* in bivalve molluscs, herbs and spices and *Vibrio* in crustaceans. It is important that RASFF annual reports often highlighted a growing trend in the number of *Salmonella* sp. notifications [19]. In turn, an increase in the number of notifications regarding presence of *Listeria monocytogenes* in meat, poultry, seafood and dairy products in 2000–2012 was noticed by Allerberger [20].

However, a more detailed analysis related to pathogenic microorganisms was presented only in RASFF annual reports for 2016 and 2017. Pathogenic microorganisms were the most frequently notified hazard category in products originating from the EU countries in these years. If we take into account non-EU countries, it was the second category in 2016, and the first in 2017. In the case of EU countries the reports indicated notifications on *Salmonella* spp. and *Salmonella enteritidis* in poultry meat and meat, *Salmonella enteritidis* in egg products, *Listeria monocytogenes* in fish (smoked salmon), meat, cheese and *Escherichia coli* in bivalve molluscs. The products with *Salmonella* sp. originated mainly from Poland, Netherlands, Belgium, France, Germany, Italy and Spain. In the case of products with *Listeria* the countries of origin were mainly France, Netherlands, Belgium, Germany, Italy, Poland and Spain, whereas, in the case of *Escherichia*, the products originated predominantly from France, Spain, Italy, Germany and United Kingdom. Recurrent notifications were notifications on *Salmonella enteritidis* in fresh poultry from Poland, *Listeria monocytogenes* in cheese from France and *Escherichia coli* in live mussels from Spain. In turn, in the case of products originated from non-EU countries notifications on *Salmonella* spp. in fruits, vegetables, poultry meat, herbs, spices and nuts had dominant share. *Escherichia coli* was notified in meat, herbs and spices. Recurrent notifications were on *Salmonella* sp. in betel leaves from India, chicken meat from Thailand and turkey and chicken meat from Brazil. A rapid increase of these notifications in 2017 was related to a fraud of certification (Figure 1a,b,d) [19].

Studies covering several-year periods carried out by other authors confirm that pathogenic microorganisms notified in the RASFF were characteristic for given products. Considering years 2003–2007 Kleter et al. indicated notifications on *Salmonella* sp. in meat, poultry and animal feed, *Listeria monocytogenes* in dairy, meat, poultry and seafood products, *Escherichia coli* in seafood, spices, condiments and *Vibrio* in seafood [21]. Notifications on *Salmonella* sp. in poultry meat and *Escherichia coli* in meat in 2008–2013 were also noticed by Jansen et al. [22]. In turn, Van Asselt et al. noted RASFF notifications on *Salmonella* spp., *Listeria monocytogenes* and *Escherichia coli* in cheese and milk in 2009–2014 [23]. D.’Amico et al. analysed RASFF notifications on seafood in 2011–2015. They stated that *Salmonella* spp. was reported in bivalve molluscs and crustaceans, *Listeria monocytogenes* in fish and crustaceans, *Escherichia coli* in bivalve molluscs and *Vibrio* in crustaceans [24]. However, notifications related to pathogenic microorganisms in seafood were also signaled by other authors: *Salmonella* spp., *Listeria monocytogenes* and *Vibrio* in frozen fish in 2005–2010 [25], *Salmonella* sp. and *Listeria* in pangasius in 2005, 2009 and 2010 [26], *Vibrio* in shrimps in 2005 and 2008 [27], *Salmonella* sp. and *Escherichia coli* in bivalve molluscs in 2009–2011 [28].

These microorganisms were also often notified in similar products in other warning systems. *Salmonella* sp. and *Listeria monocytogenes* were the most frequently reported hazards in 2009–2014 in the Reportable Food Registry maintained by the United States Food and Drug Administration. They accounted for over 56% of all so-called primary entries. *Salmonella* sp. was reported mainly in animal food/feed, nuts/nut products/seed products, raw agriculture commodities, spices and seasonings. In turn, *Listeria monocytogenes* was notified in dairy products, prepared foods, fresh cut and raw agriculture commodities and also seafood [29]. These hazards were also often reported in 2014–2015 in the International Food Safety Authorities Network managed by the Food and Agriculture Organization of the United Nations and the World Health Organization. *Salmonella enterica* spp. was notified mainly in eggs and nuts, *Listeria monocytogenes* in meat and fruits and *Escherichia coli* in meat [30].

### 4.3. Food Poisoning Caused by Microorganisms Based on RASFF Reports

Data for foodborne outbreaks in the United States in 1998–2008 indicated that food, mainly poultry, beef, leafy vegetables, fruits and nuts, was identified as a vehicle of 60% of outbreaks [31]. Among different microorganisms *Salmonella* spp., *Listeria monocytogenes* and *Escherichia coli* were listed as responsible for the majority of foodborne illnesses outbreaks [32,33]. It is important that Hoagland et al. noticed that almost half of outbreaks in the United States were linked to consumption of fresh fruits and vegetables [34]. In turn, Esbelin et al. noted that the number of food-borne bacterial outbreaks raised over the past decade due to an increasing role of fruits and vegetables as pathogen transmission [35].

Cases of food poisoning including also (suspected) foodborne outbreaks have been identified in the RASFF since 2008. Most of them concerned poisoning with norovirus, histamine or hepatitis A [19]. However, it is worth paying particular attention to notifications related to the discussed microorganisms, i.e., *Salmonella* sp., *Listeria* and *Escherichia*, reported as alert notifications with more than one person affected (Table 1). In the annual reports for 2016 and 2017 the data concerned only selected cases of food poisoning.

Therefore, the cases presented in Table 1 mainly referred to *Salmonella* sp. in food of animal origin (meat and poultry—see Figure 1b, eggs and milk). The products usually originated from EU countries, which indirectly indicated effectiveness of border controls. On the other hand, it is worrying that there was a possibility of spreading hazards (multi-country outbreaks) due to the free food flow within the internal market. However, in many cases, the notifying country was the country from which the notified product originated. It proved responsibility of the national surveillance authorities in ensuring safety for European consumers. It worth noting, however, that according to the data from Eurostat within the Broad Economic Categories (BEC) the intra food (and beverage) flow (including all 28 countries) increased almost twice in the period 2000–2017 and in 2017 it was three times higher than the extra food flow [36]. Therefore, there is a necessity to increase efficiency of work of the bodies supervising food safety as well as cooperation between them.

The occurrence of outbreaks in Europe or other regions caused by the discussed microorganisms in similar products was also noticed by other authors, presenting summaries covering multiannual periods. Yoon et al. drew attention to global outbreaks related to *Salmonella* sp., *Listeria monocytogenes* and *Escherichia coli* in raw (in 1983–2010) or pasteurised milk cheese (1994–2014) [37]. Similarly, Verraes et al. presented outbreaks in Europe, United States and Canada in 1982–2010 referring to *Salmonella* sp., *Listeria monocytogenes* and *Escherichia coli* due to consumption of dairy products made from raw milk [38]. In turn, outbreaks caused by these microorganisms in fresh products were noticed by Alegbeleye et al. (in period 1997–2017) [39], Mukhopadhyay and Ukuku (2011–2013) [40] and Olaimat and Holley (2005–2011) [41]. Whereas, Zweifel and Stephan pointed out to selected *Salmonella*-related outbreaks caused by spices and herbs in 1993–2010 [42]. Therefore, Hoagland et al. proposed measures to reduce the number of outbreaks associated with horticultural products, e.g., installing barriers, appropriate managing manure, soil, water and seeds, ensuring clean equipment and hygiene of employees, developing new varieties and optimizing technologies [34].

## 5. Conclusions

Notifications regarding pathogenic microorganisms in the RASFF mainly concerned *Salmonella* sp., *Listeria*, *Escherichia* and *Vibrio* (in food) and *Salmonella* sp. (in feed). In the case of non-pathogenic microorganisms notifications related to unspecified microorganisms (mainly moulds), Enterobacteriaceae, *Salmonella* sp. and Coliforms (in food) and also unspecified microorganisms (in feed). These were mostly products of animal origin, i.e., meat, poultry meat, milk, seafood (fish, crustaceans and molluscs), but also non-animal origin: fruits, vegetables, herbs, spices and nuts. The number of notifications decreased in 2005–2006 after introducing hygiene package and microbiological criteria, but has significantly increased since then. Notifications were reported usually on the basis of official controls, border controls and company’s own checks. The notifying countries were mainly: Italy, France, United Kingdom, Germany and Netherlands and notified products originated mostly from Brazil, East Asian countries (India, Thailand and Vietnam) and EU countries (Germany, France, Poland, Italy, Spain and Netherlands). The largest number of notifications were alerts, followed by information notifications and border rejections. Distribution status of notified products was often not specified, however, distribution was also indicated as possible, restricted to the notifying country, the product could be also not placed on the market or not distributed. The decision on risk was usually not made. The notified products were re-dispatched, withdrawn from the market, import was not authorised, products were also destroyed or recalled from the market. Although the role of the RASFF seems to be only reactive, it significantly contributes to ensuring public health related to microorganisms on the common, European market with the free flow of products. The analysis of trends in notifications can be helpful in shaping law binding in all EU countries. Besides, the obligation to notify hazards to the European Commission allows not only minimizing their effects, but also limiting a possibility of spread of potential outbreaks. The activity of individual members in the RASFF can depend on the size of the domestic market, as well as the structure and experience of supervisors. Nevertheless, cooperation between EU countries and the European Commission within notifications concerning microorganisms should be based on mutual trust. However, it is also important to pay attention to proper reporting of notifications in the RASFF, because some data in the database is missing or it is incomplete or inconclusive.

## Figures and Tables

**Table 1:** Selected cases of food poisoning notified in the RASFF in 2008–2017.

Year	Microorganism	Product	Notifying Country	Origin Country	Persons Affected
2008	*Salmonella enteritidis*	frozen whole hens	Estonia	Lithuania	83
	*Salmonella agona*	cooked beef steak	Ireland	Ireland	n/a (large outbreak)
	*Salmonella kedougou*	infant formula	Spain	Spain	23 (outbreak)
	*Salmonella enteritidis*	eggs	France	Spain	6 (outbreak)
2009	*Salmonella bovismorbificans*	alfalfa seeds	Finland	Italy	20 (outbreak)
	*Salmonella enteritidis*	eggs	France	Spain	30 (outbreak)
	*Salmonella enteritidis, Salmonella typhimurium*	eggs	France	Spain	8
	*Salmonella enteritidis*	eggs	Great Britain	Spain	n/a (large outbreak)
2010	*Listeria monocytogenes*	cheese	Austria	Austria	24
	*Salmonella* spp.	salami	Italy	Italy	n/a
	*Escherichia coli*	cheese	Romania	Bulgaria	7
	*Salmonella typhimurium*	dried sausages	France	France	88 (outbreak)
	*Salmonella typhimurium*	frozen beef burgers	France	Italy	554
	*Listeria monocytogenes*	ham sausages	Czech Republic	Czech Republic	2
2011	*Escherichia coli* (shigatoxin)	organic sprouts	Germany	Germany	n/a (large outbreak)
	*Salmonella*	frozen seafood mix	Sweden	Vietnam	58
	*Escherichia coli* (verotoxin)	fenugreek seeds	France	Egypt	n/a (large outbreak)
	*Escherichia coli*	sugar peas	Denmark	Kenya	87 (outbreak)
	*Salmonella enteritidis*	frozen duck breasts	Iceland	Netherlands	8
	*Salmonella Strathcona*	datterino tomatoes	Denmark	Italy	40 (outbreak)
	*Salmonella monophasic serovar*	dried sausages	France	France	n/a (outbreak)
2012	*Salmonella Oranienburg*	dried milk formula	Belgium	Belgium	16 (outbreak)
	*Salmonella* spp.	chilled stuffed meat	Italy	Romania	3
	*Salmonella Dublin*	raw milk cheese	France	France	n/a (large outbreak)
	*Salmonella Bredeney*	peanut products	Commission services	United States	41 (outbreak)
2013	*Escherichia coli* (shigatoxin)	frozen hamburgers	Sweden	Sweden	2
	*Salmonella enteritidis*	eggs	France	Spain	49 (outbreak)
2014	*Salmonella kedougou*	raw milk cheese	France	France	25
	*Salmonella enteritidis*	frozen pork	Slovakia	Hungary	164 (outbreak)
	*Salmonella enteritidis*	eggs	France	Germany	9 (outbreak)
	*Salmonella enteritidis*	eggs	Austria	Germany	20 (outbreak)
	*Salmonella enteritidis*	eggs	France	Germany	3 (outbreak)
	*Listeria monocytogenes*	lamb-roll sausages	Denmark	Denmark	20 (outbreak)
	*Salmonella* spp.	liquid egg whites	United Kingdom	France	2
2015	*Salmonella enteritidis*	frozen minced beef	France	Poland	49
	*Salmonella enteritidis*	frozen minced beef	France	Poland	22
	*Salmonella Rissen*	frozen minces meat	France	Italy	11
	*Escherichia coli* (shigatoxin)	raw milk cheese	Ireland	Ireland	2
	*Salmonella enteritidis*	raw milk	France	France	15
	*Salmonella typhimurium*	beef	Netherlands	Netherlands	44
2016	*Escherichia coli* (shigatoxin)	fermented cheese	Italy	Romania	25 (outbreak)
	*Salmonella enteritidis*	eggs	n/a	Poland	n/a (outbreak)
2017	*Salmonella enteritidis*	eggs	n/a	Poland	n/a (outbreak)
	*Salmonella typhimurium*	salami	Sweden	Spain	n/a (outbreak)
	*Salmonella agona*	infant formula	France	France	n/a (outbreak)

Source: own study on the basis of [19].

## Supplementary Materials

The following are available online at http://www.mdpi.com/1660-4601/16/3/477/s1, Figures S1–S9. Similarities of RASFF notifications on pathogenic microorganisms and year (S1); product category (S2); notifying country (S3); origin country (S4); notification basis (S5); notification type (S6); distribution status (S7); risk decision (S8); action taken (S9) within food using joining; Figures S10–S18. Similarities of RASFF notifications on pathogenic microorganisms and year (S10); product category (S11); notifying country (S12); origin country (S13); notification basis (S14); notification type (S15); distribution status (S16); risk decision (S17); action taken (S18) within feed using joining; Figures S19–S27. Similarities of RASFF notifications on pathogenic microorganisms and year (S19); product category (S20); notifying country (S21); origin country (S22); notification basis (S23); notification type (S24); distribution status (S25); risk decision (S26); action taken (S27) within food using two-way joining; Figures S28–S36. Similarities of RASFF notifications on pathogenic microorganisms and year (S28); product category (S29); notifying country (S30); origin country (S31); notification basis (S32); notification type (S33); distribution status (S34); risk decision (S35); action taken (S36) within feed using two-way joining. Figures S37–S45. Similarities of RASFF notifications on non-pathogenic microorganisms and year (S37); product category (S38); notifying country (S39); origin country (S40); notification basis (S41); notification type (S42); distribution status (S43); risk decision (S44); action taken (S45) within food using joining; Figures S46–S54. Similarities of RASFF notifications on non-pathogenic microorganisms and year (S46); product category (S47); notifying country (S48); origin country (S49); notification basis (S50); notification type (S51); distribution status (S52); risk decision (S53); action taken (S54) within feed using joining; Figures S55–S63. Similarities of RASFF notifications on non-pathogenic microorganisms and year (S55); product category (S56); notifying country (S57); origin country (S58); notification basis (S59); notification type (S60); distribution status (S61); risk decision (S62); action taken (S63) within food using two-way joining; Figures S64–S72. Similarities of RASFF notifications on non-pathogenic microorganisms and year (S64); product category (S65); notifying country (S66); origin country (S67); notification basis (S68); notification type (S69); distribution status (S70); risk decision (S71); action taken (S72) within feed using two-way joining.
