# Supplementary material for: Pathogenic and Non-Pathogenic Microorganisms in the Rapid Alert System for Food and Feed
Source: Int J Environ Res Public Health. 2019 Feb 6;16(3):477. doi: 10.3390/ijerph16030477 (PMC6388125; doi:10.3390/ijerph16030477)
Supplement: Supplementary file 1 [file ijerph-16-00477-s001.pdf]

Supplementary Materials: Pathogenic and non-pathogenic microorganisms in the Rapid Alert System for Food and Feed
(pathogenic microorganisms)

Marcin Pigłowski

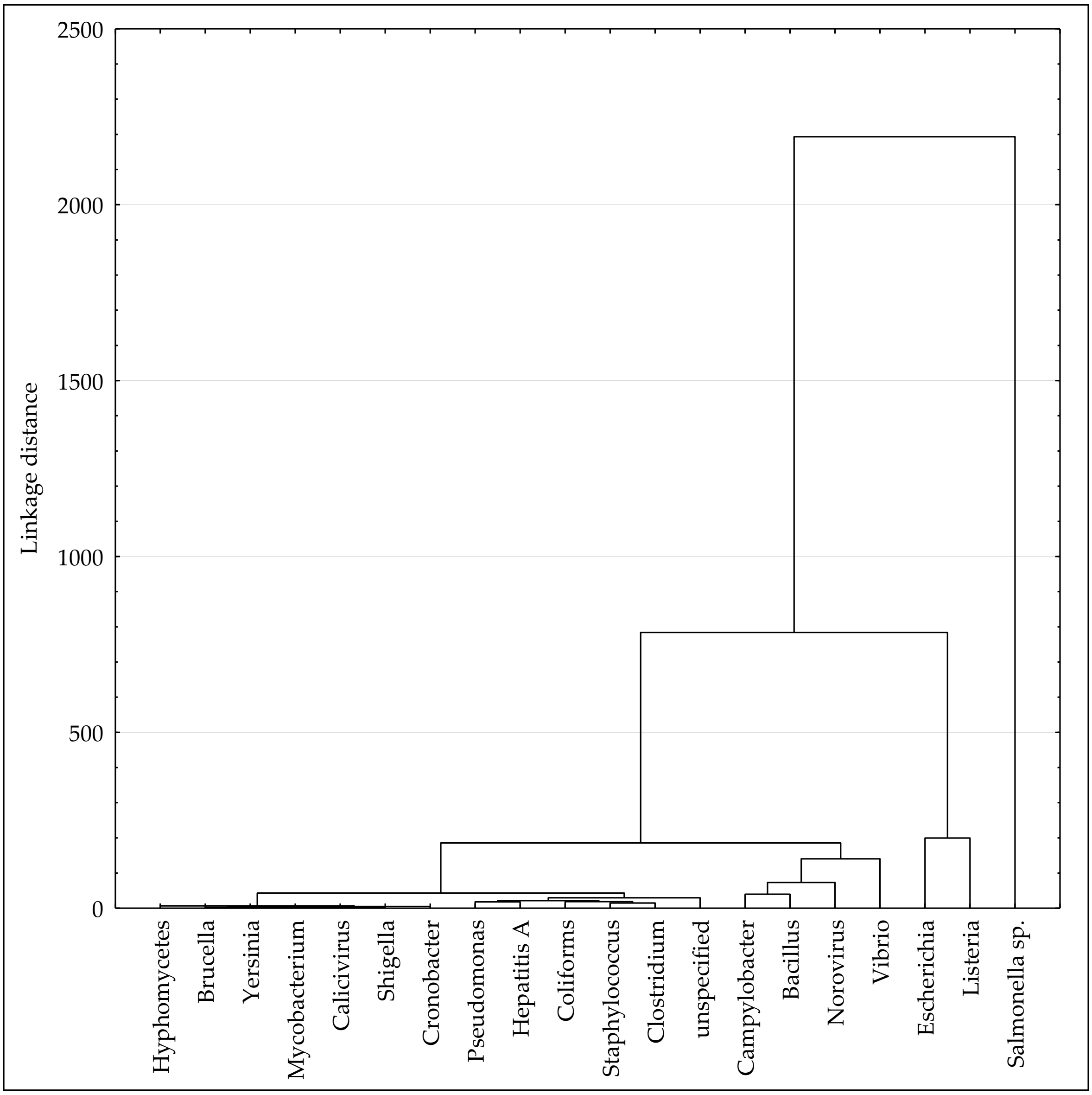

Figure S1. Similarities of RASFF notifications on pathogenic microorganisms and year within food using joining.

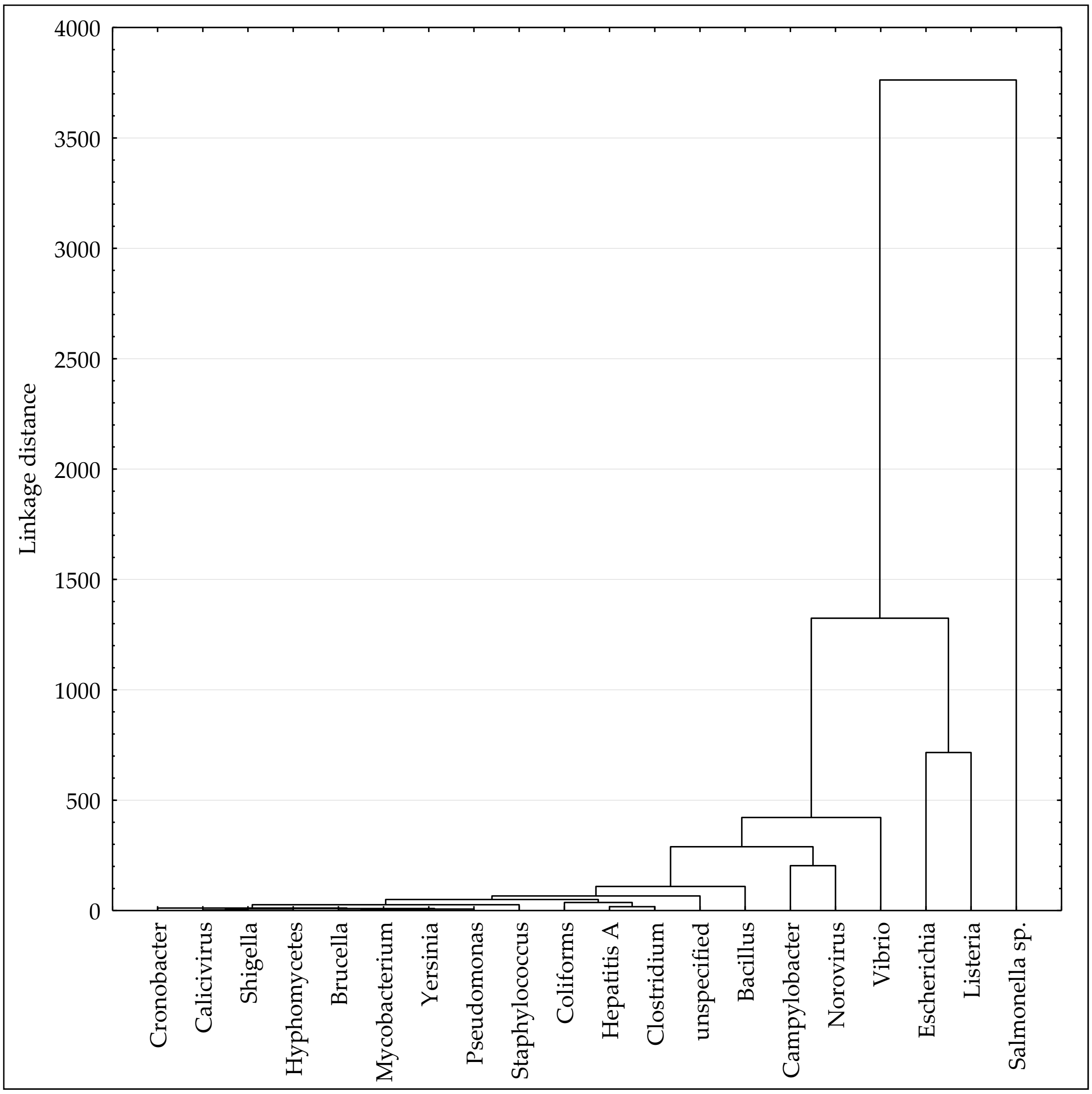

Figure S2. Similarities of RASFF notifications on pathogenic microorganisms and product category within food using joining.

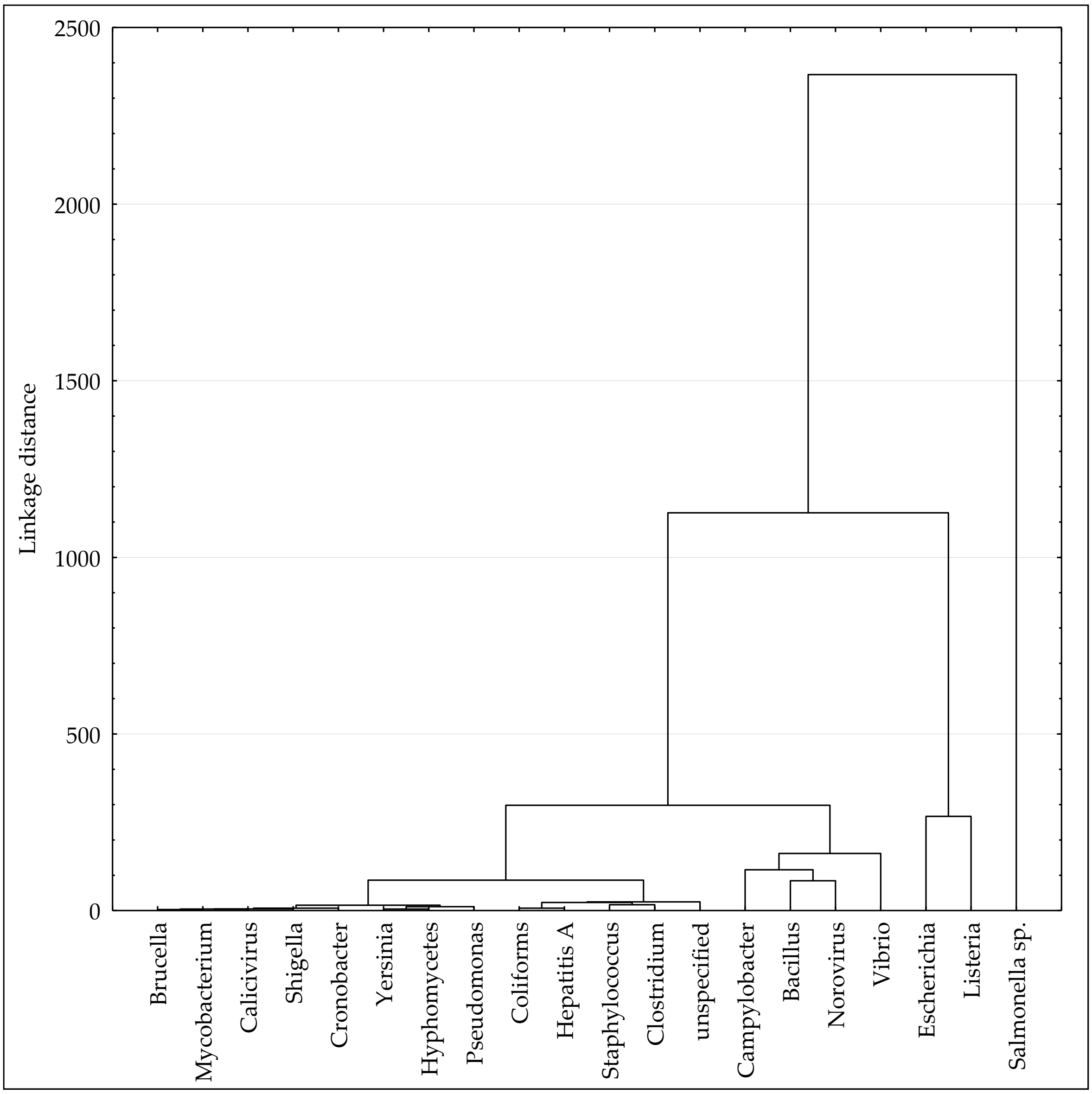

Figure S3. Similarities of RASFF notifications on pathogenic microorganisms and notifying country within food using joining.

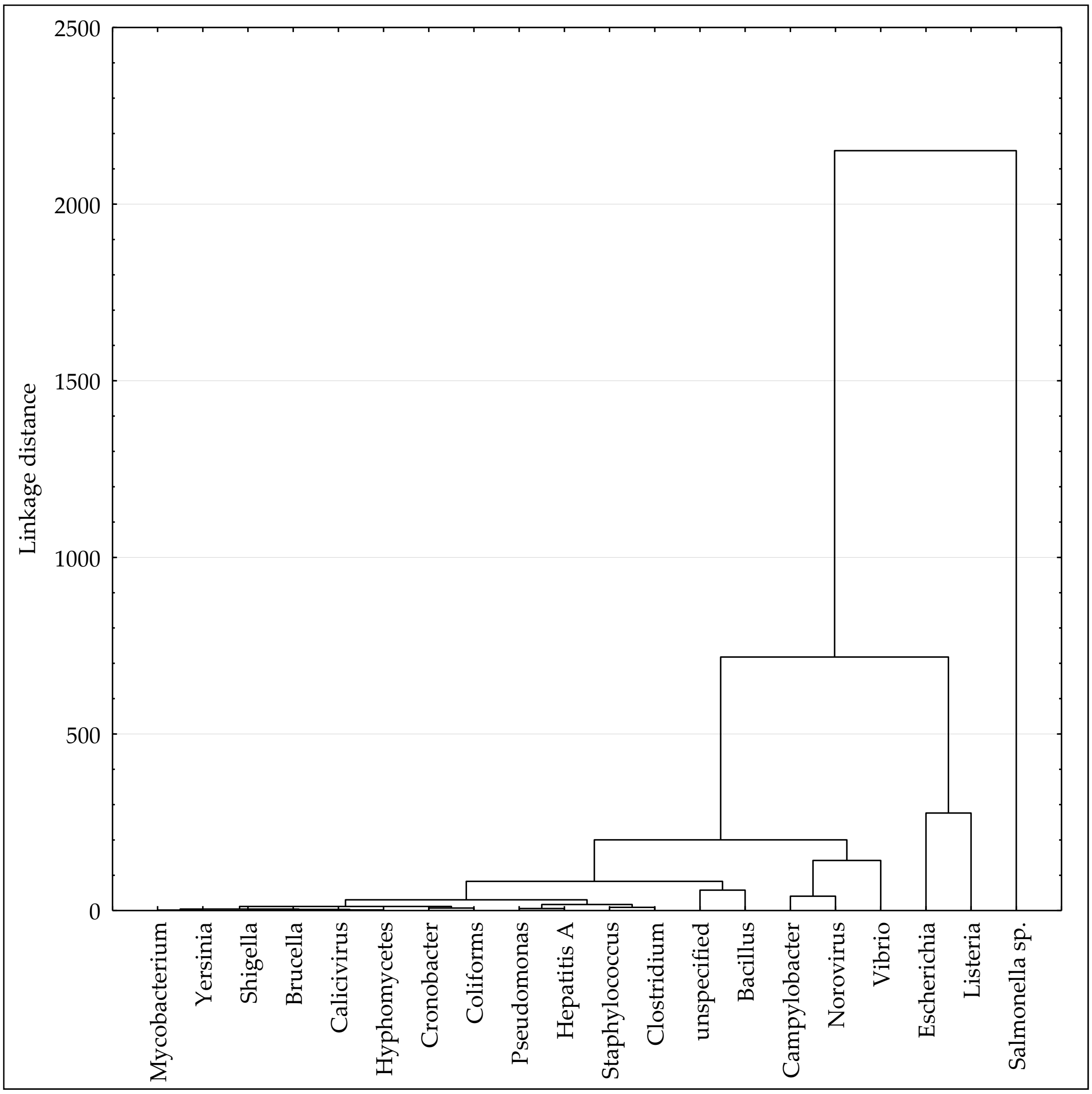

Figure S4. Similarities of RASFF notifications on pathogenic microorganisms and origin country within food using joining.

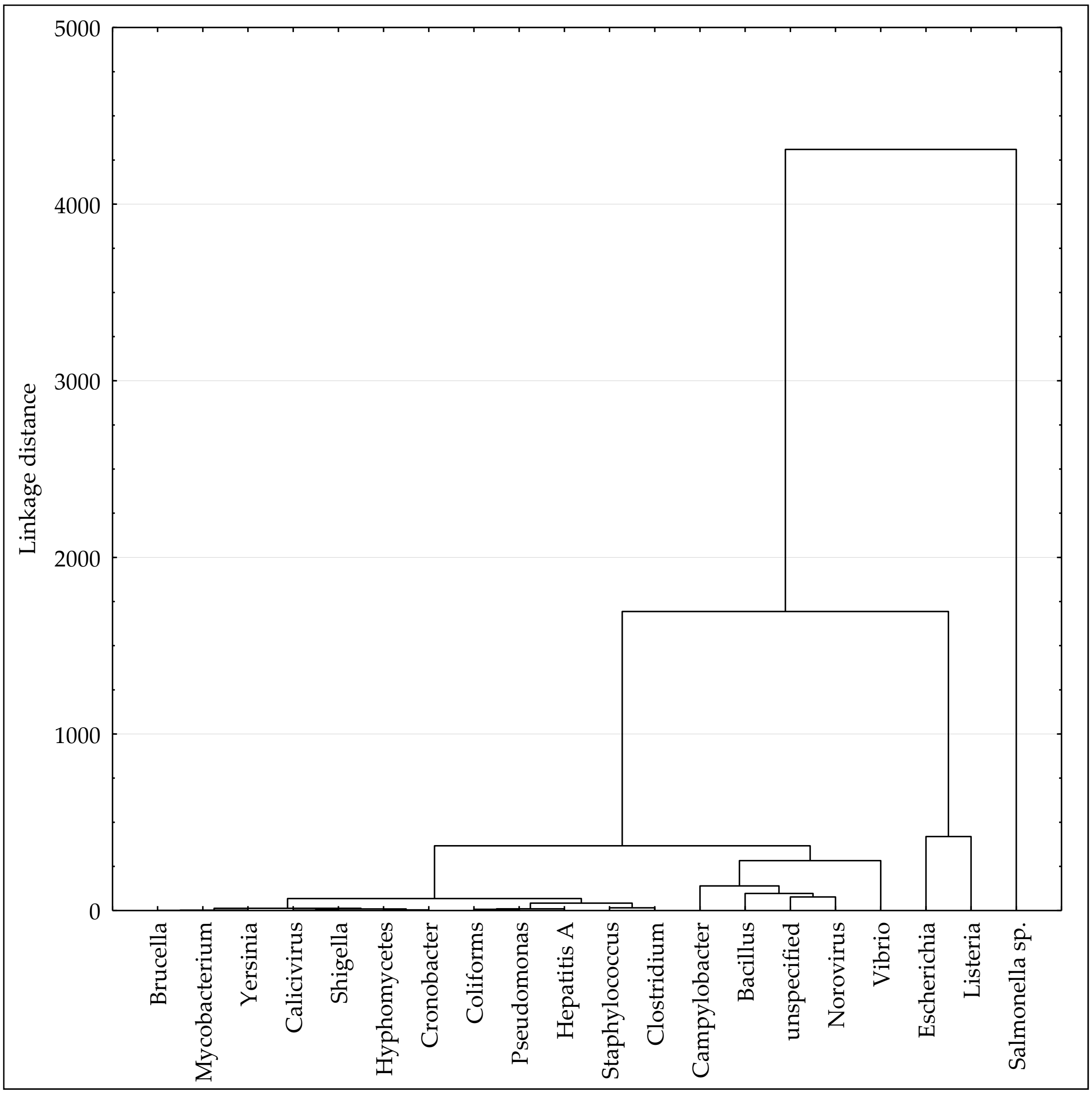

Figure S5. Similarities of RASFF notifications on pathogenic microorganisms and notification basis within food using joining.

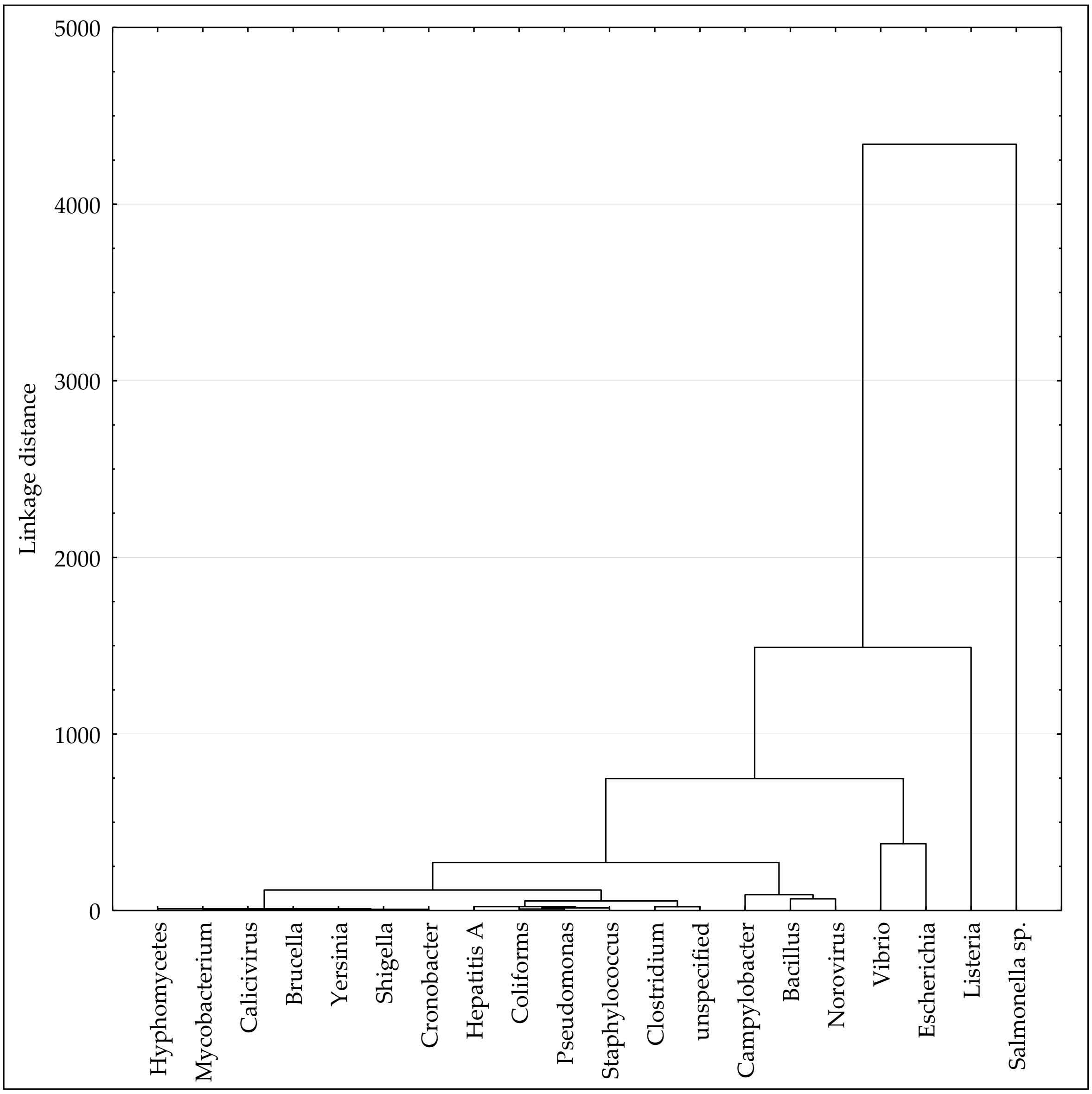

Figure S6. Similarities of RASFF notifications on pathogenic microorganisms and notification type within food using joining.

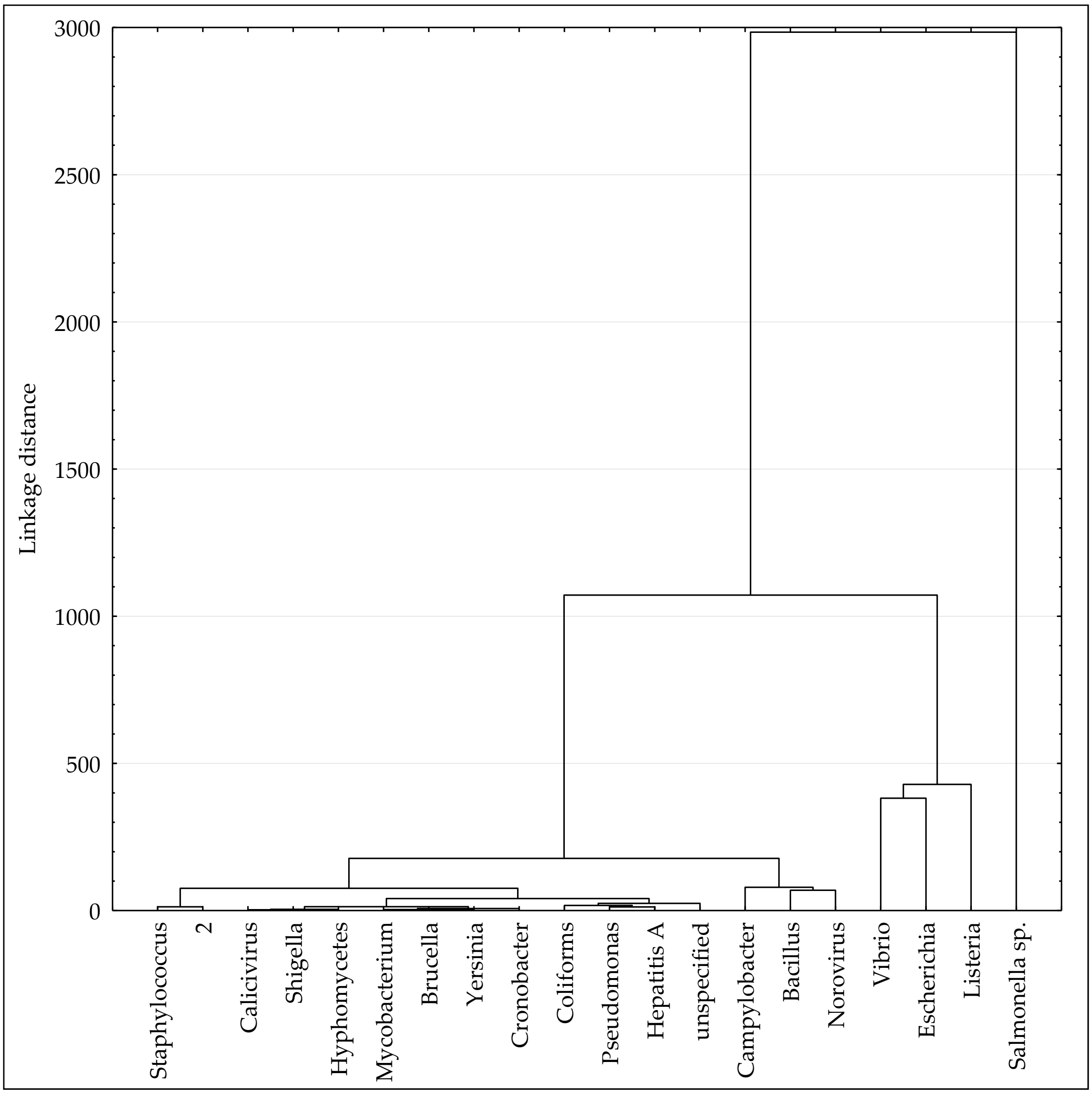

Figure S7. Similarities of RASFF notifications on pathogenic microorganisms and distribution status within food using joining.

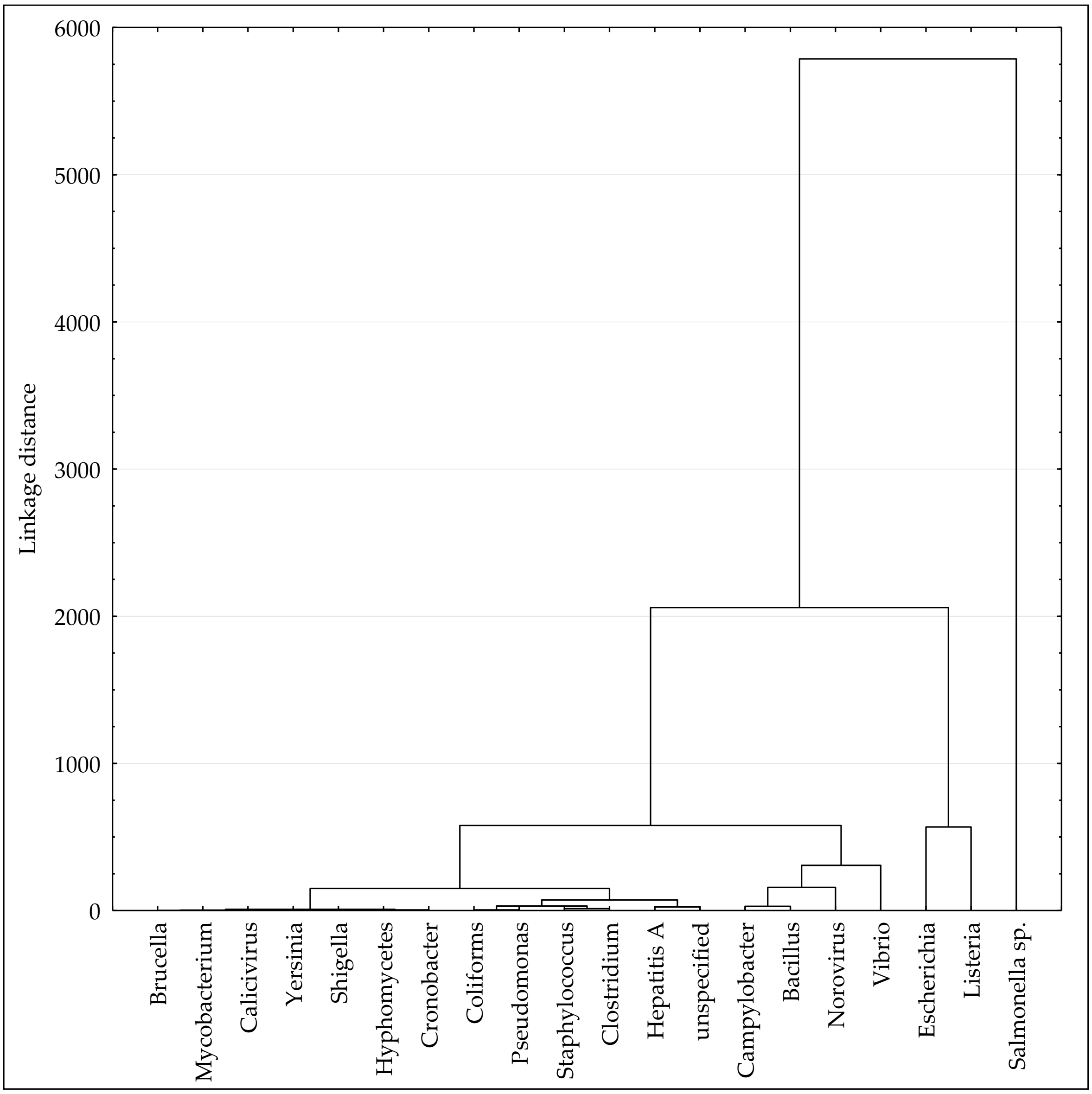

Figure S8. Similarities of RASFF notifications on pathogenic microorganisms and risk decision within food using joining.

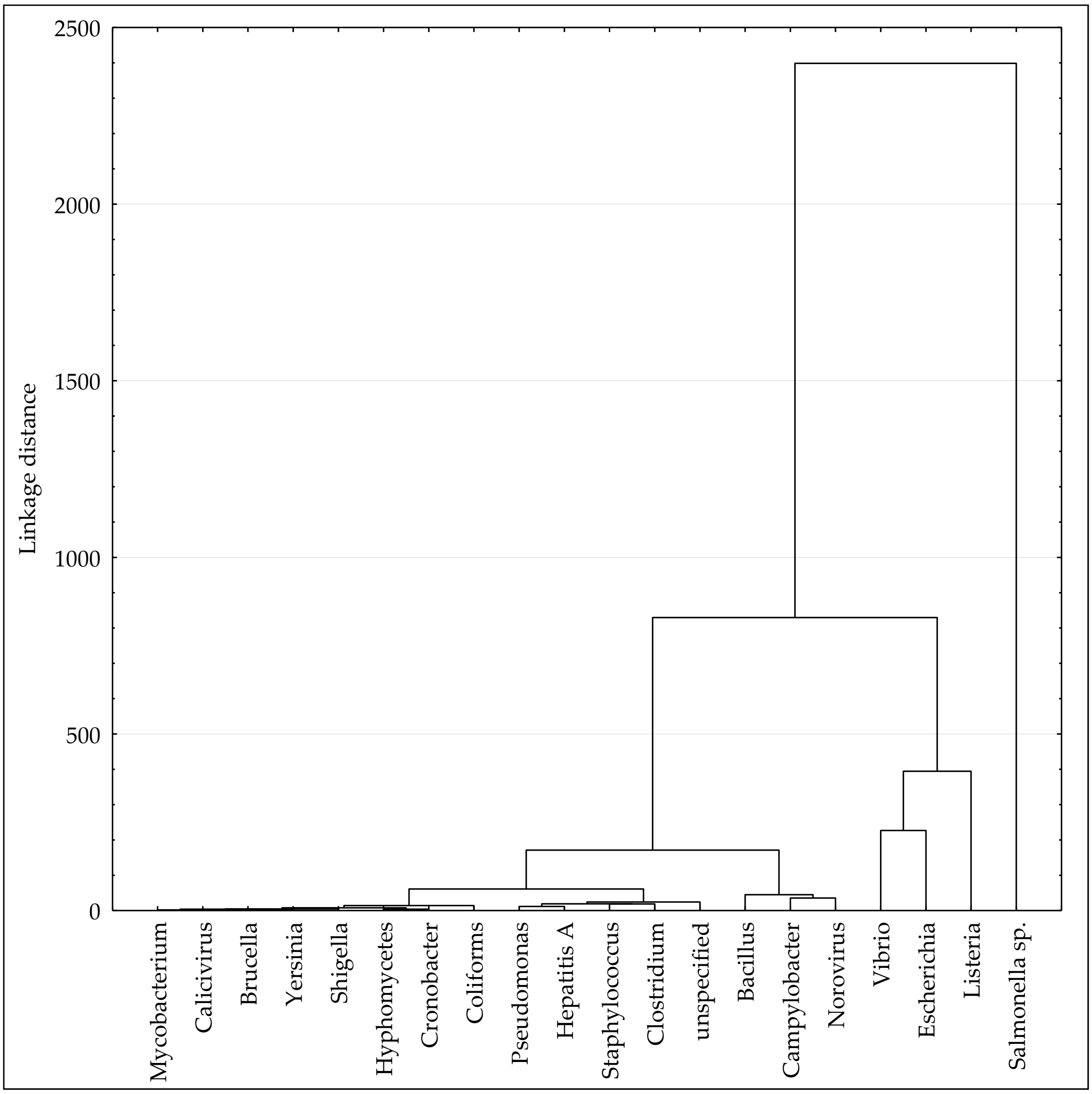

Figure S9. Similarities of RASFF notifications on pathogenic microorganisms and action taken within food using joining.

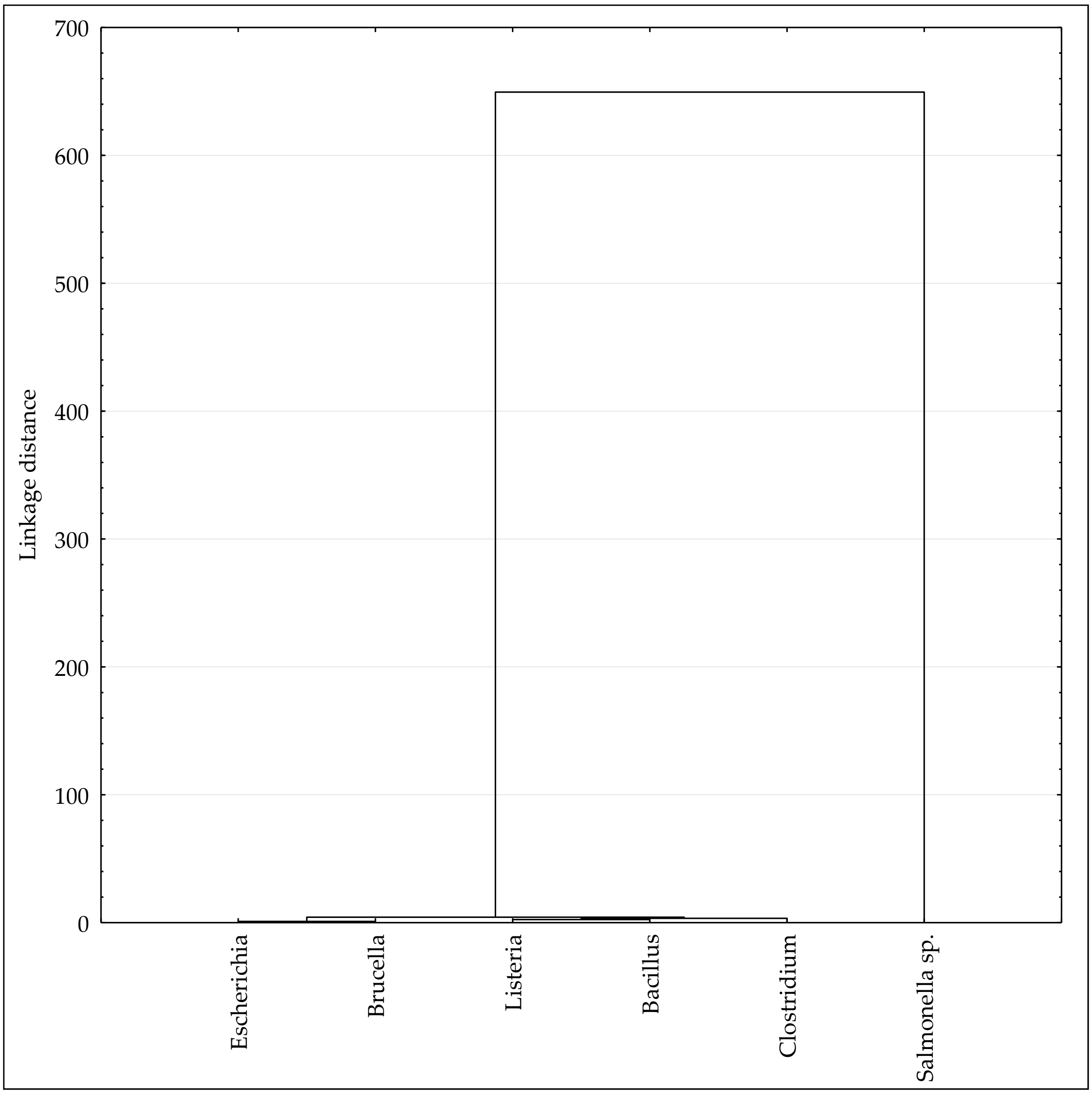

Figure S10. Similarities of RASFF notifications on pathogenic microorganisms and year within feed using joining.

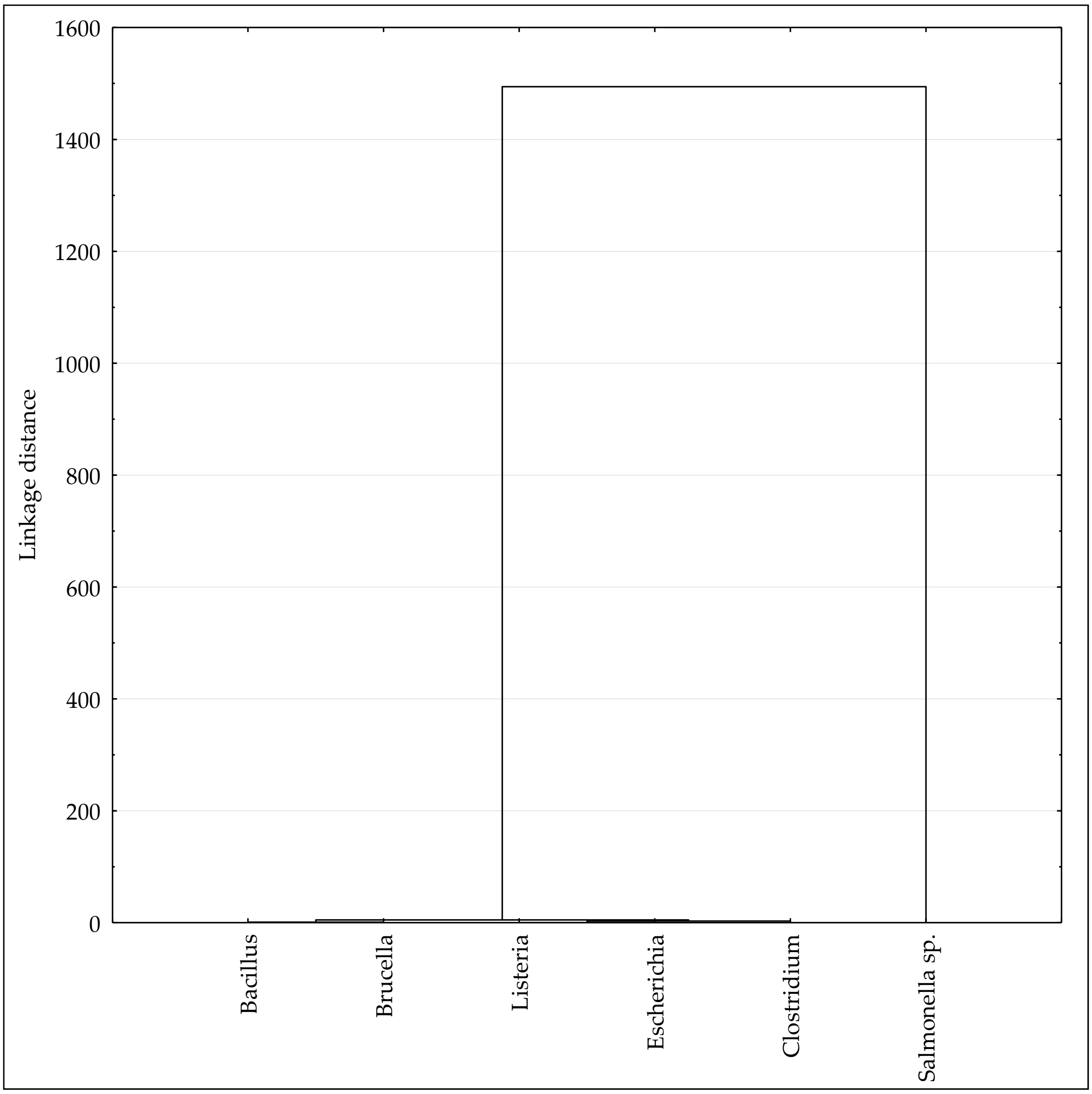

Figure S11. Similarities of RASFF notifications on pathogenic microorganisms and product category within feed using joining.

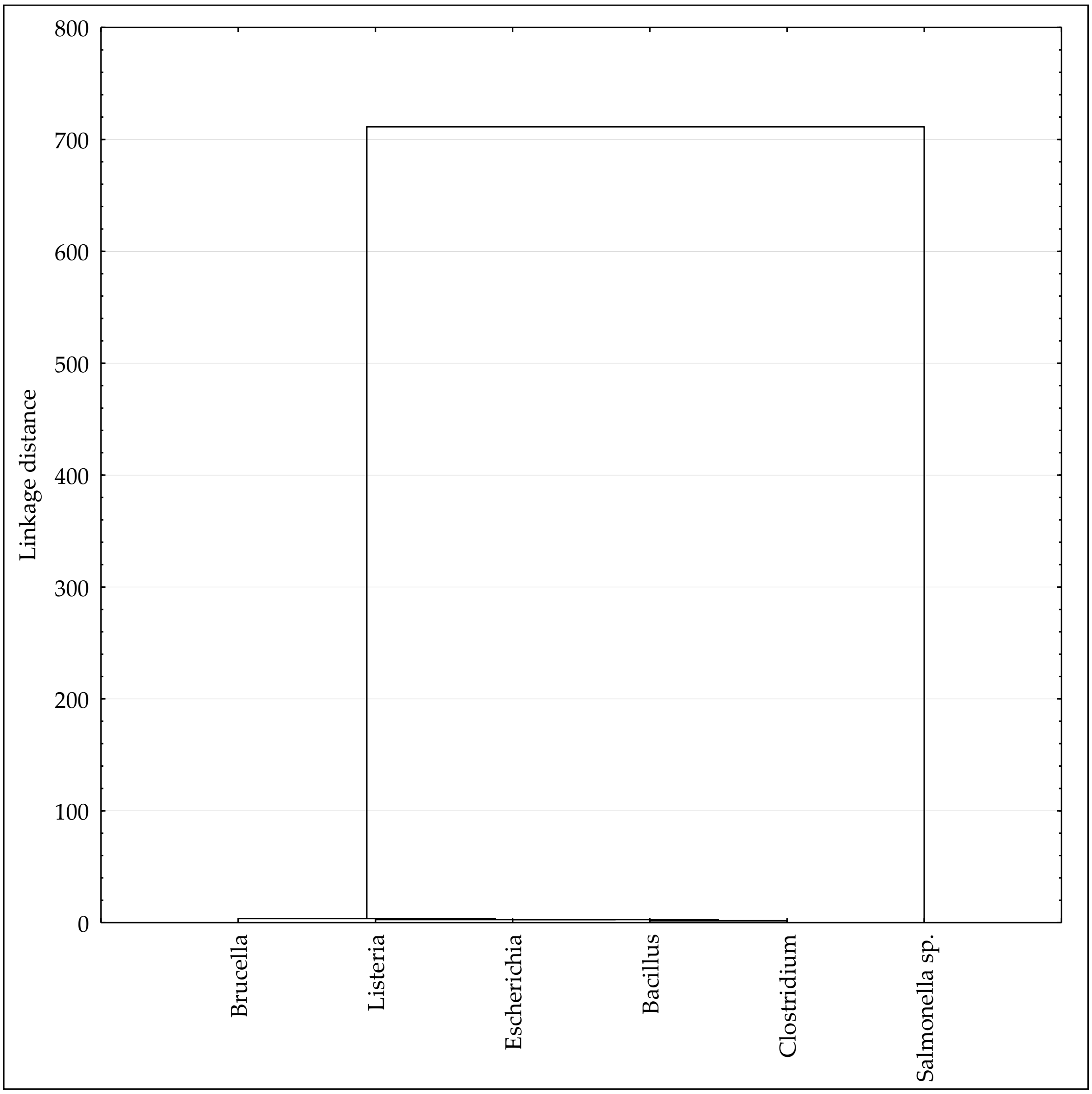

Figure S12. Similarities of RASFF notifications on pathogenic microorganisms and notifying country within feed using joining.

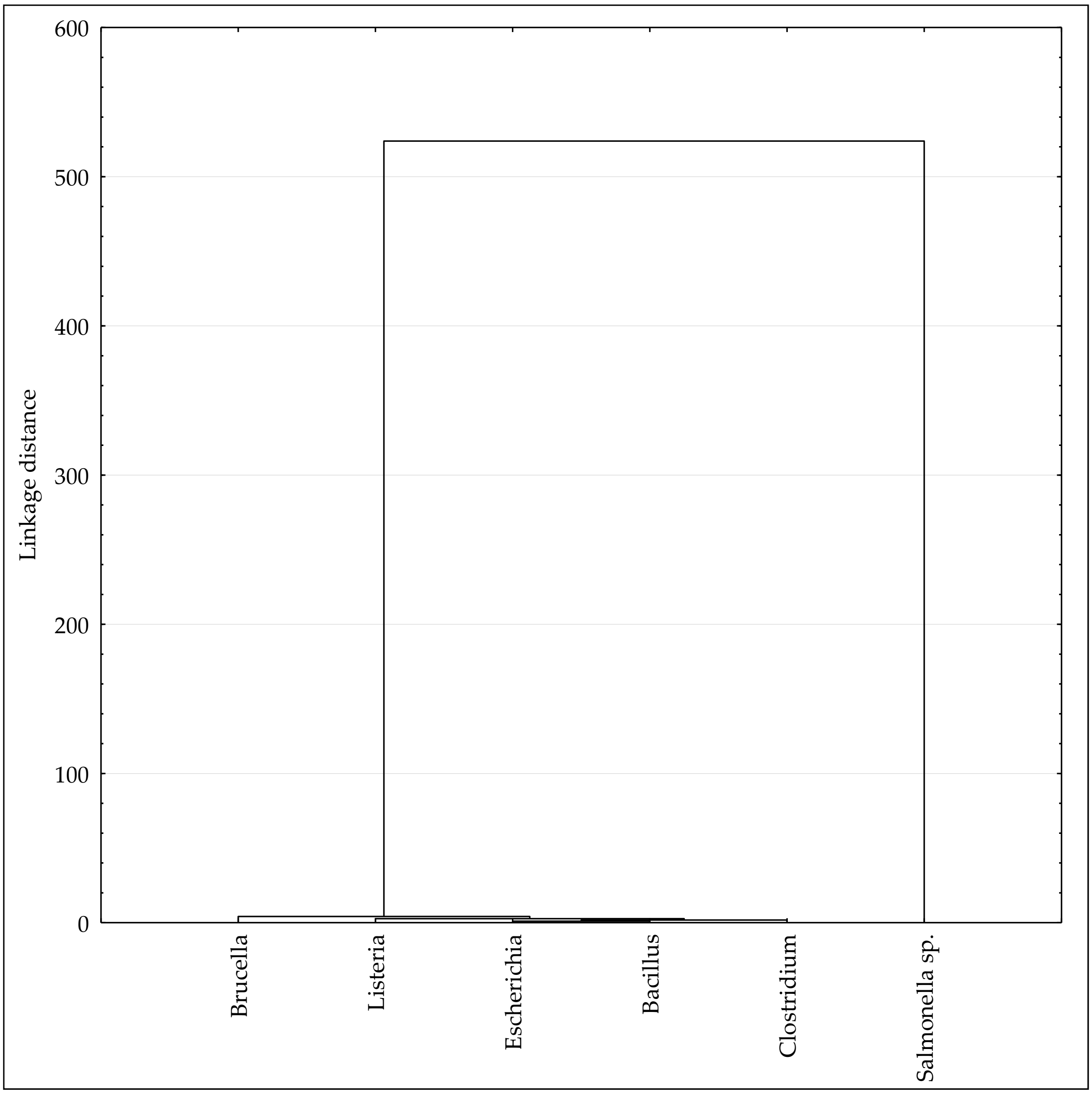

Figure S13. Similarities of RASFF notifications on pathogenic microorganisms and origin country within feed using joining.

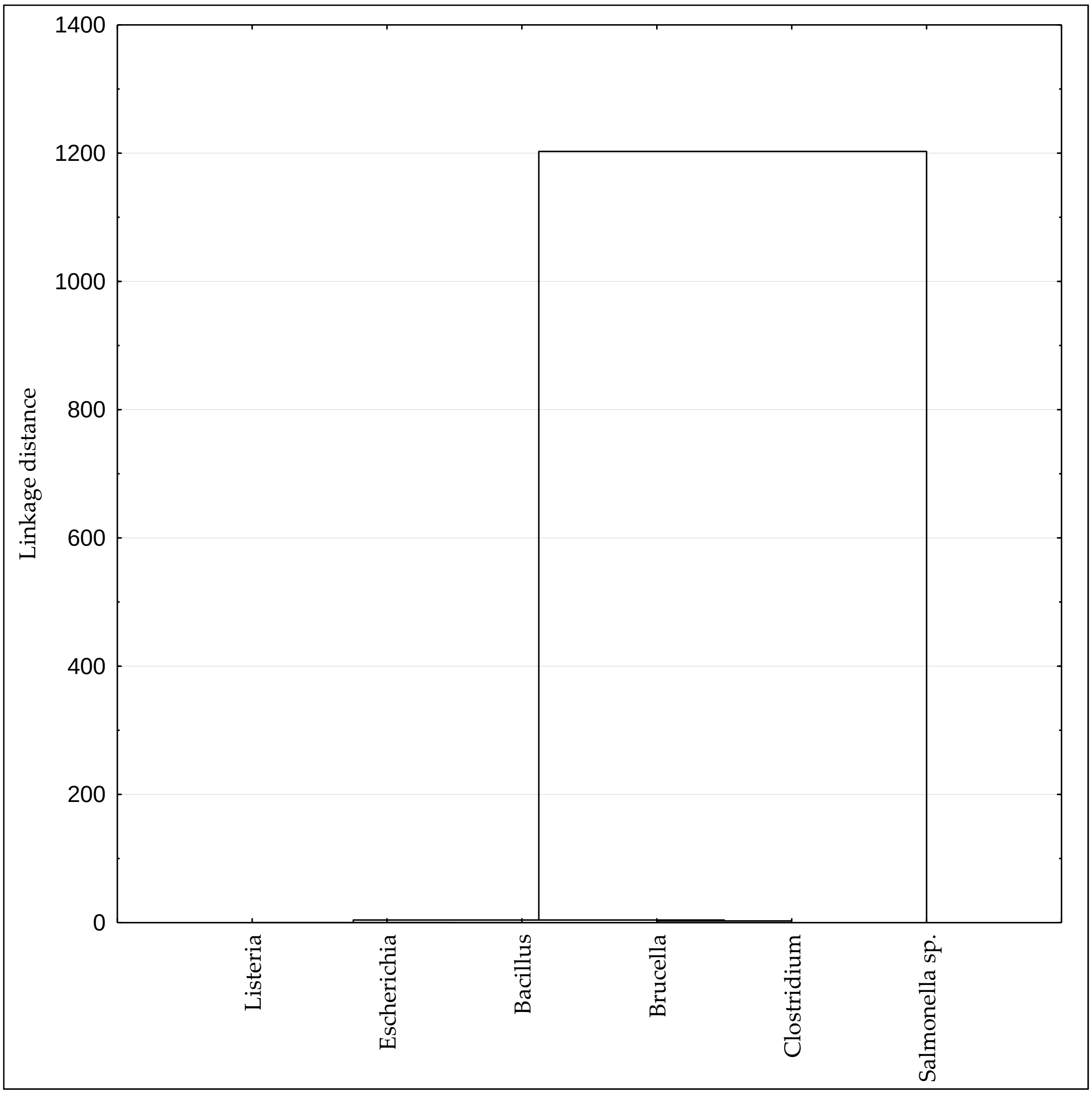

Figure S14. Similarities of RASFF notifications on pathogenic microorganisms and notification basis within feed using joining.

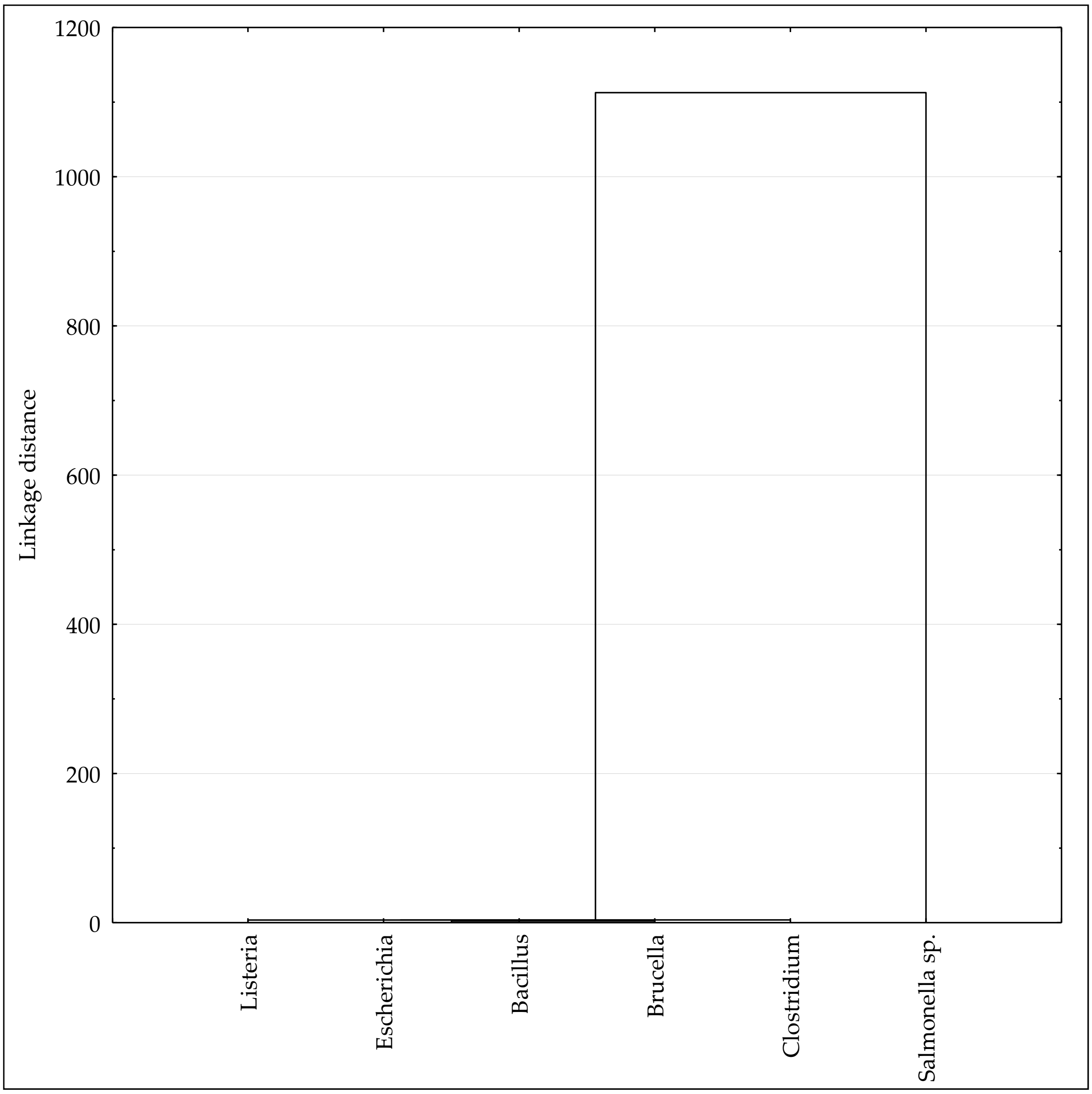

Figure S15. Similarities of RASFF notifications on pathogenic microorganisms and notification type within feed using joining.

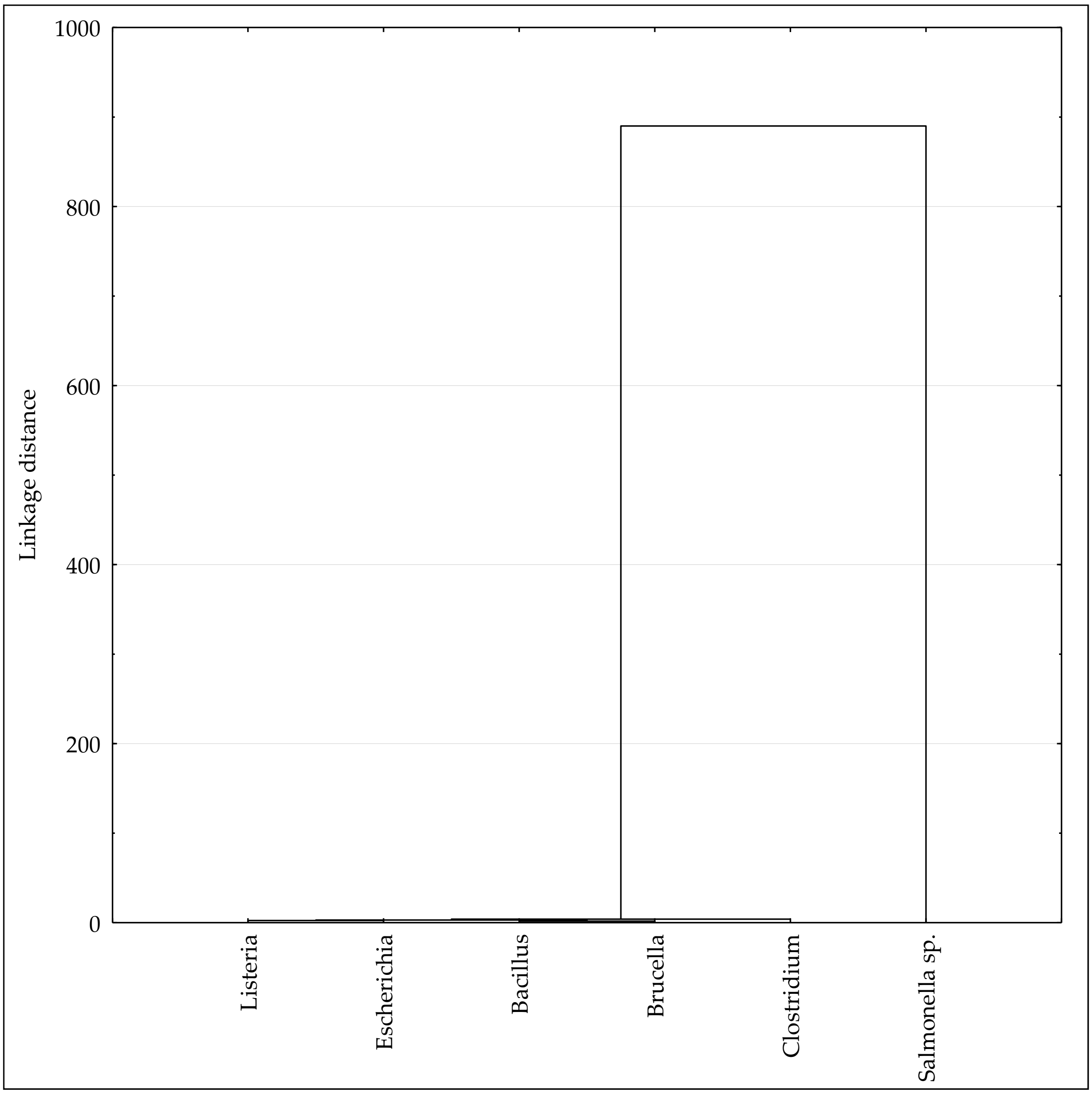

Figure S16. Similarities of RASFF notifications on pathogenic microorganisms and distribution status within feed using joining.

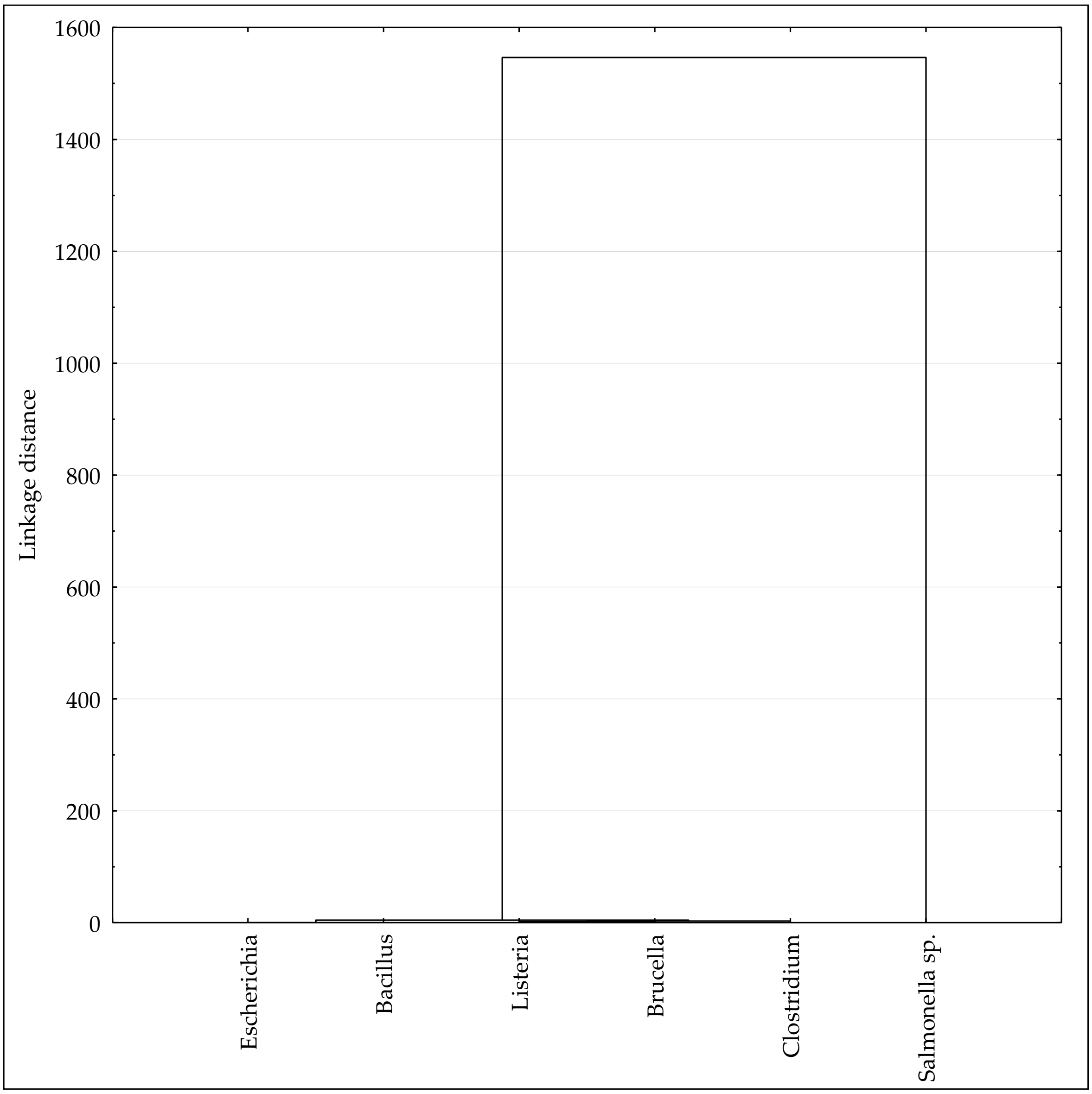

Figure S17. Similarities of RASFF notifications on pathogenic microorganisms and risk decision within feed using joining.

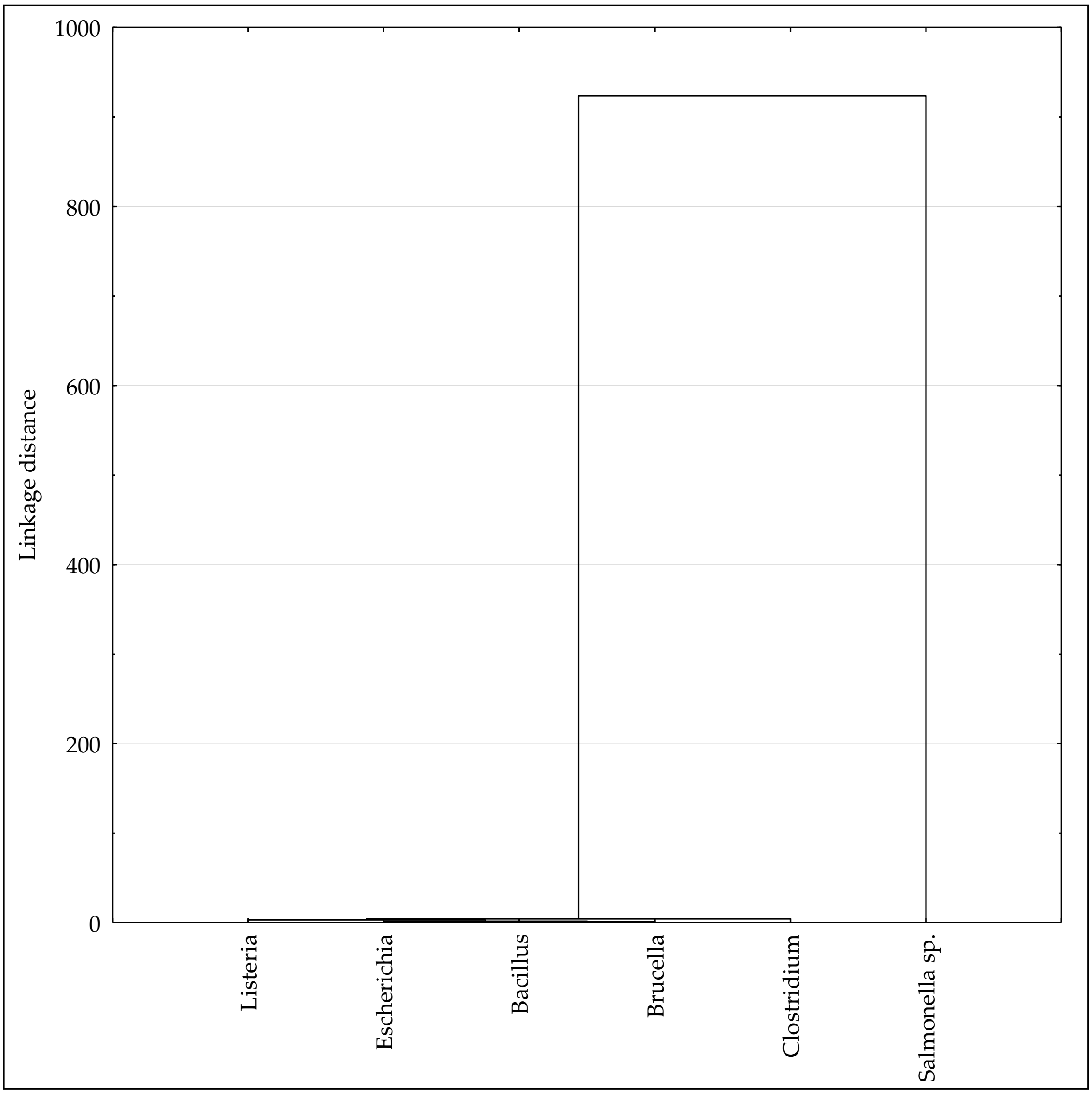

Figure S18. Similarities of RASFF notifications on pathogenic microorganisms and action taken within feed using joining.

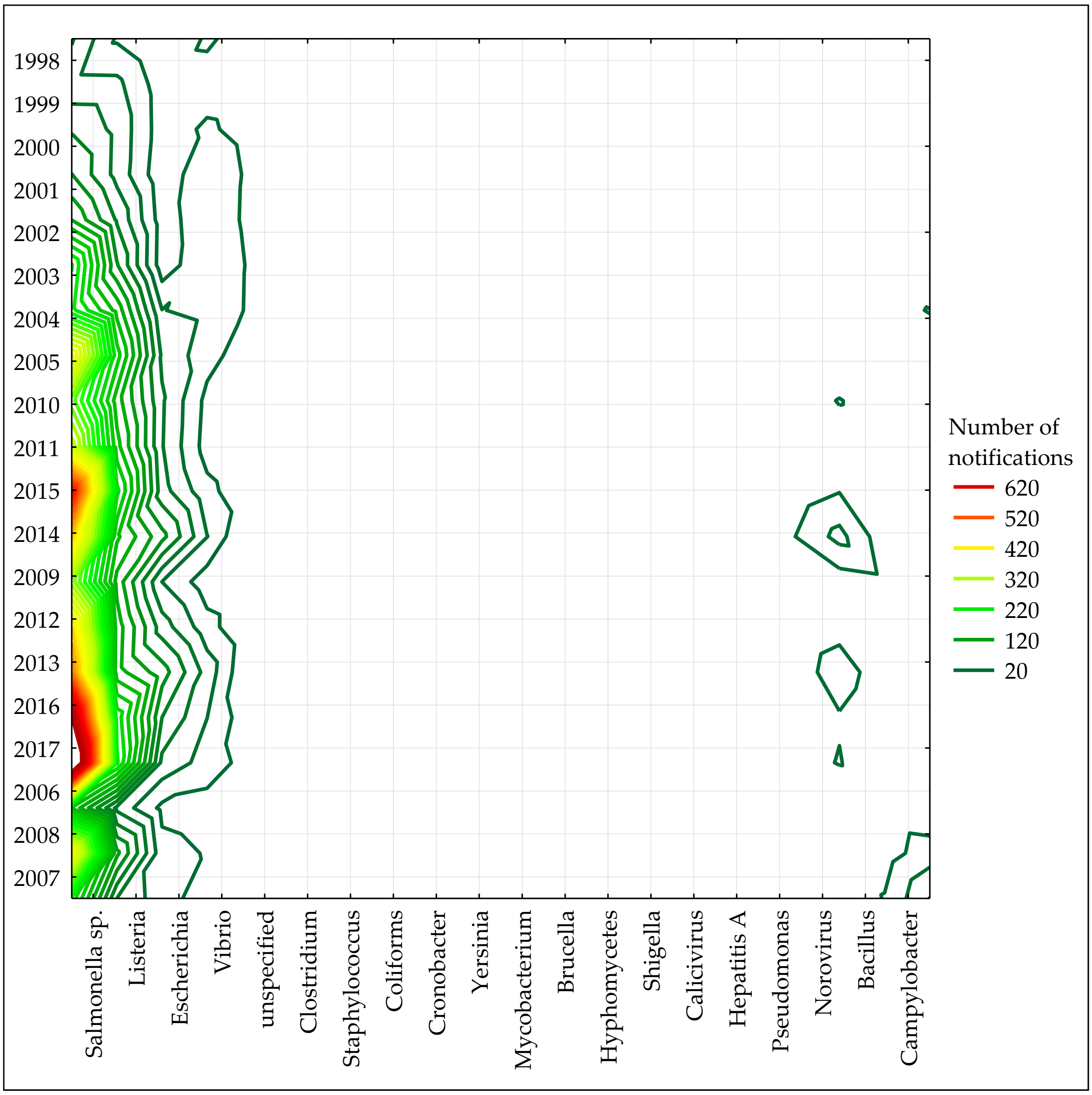

Figure S19. Similarities of RASFF notifications on pathogenic microorganisms and year within food using two-way joining.

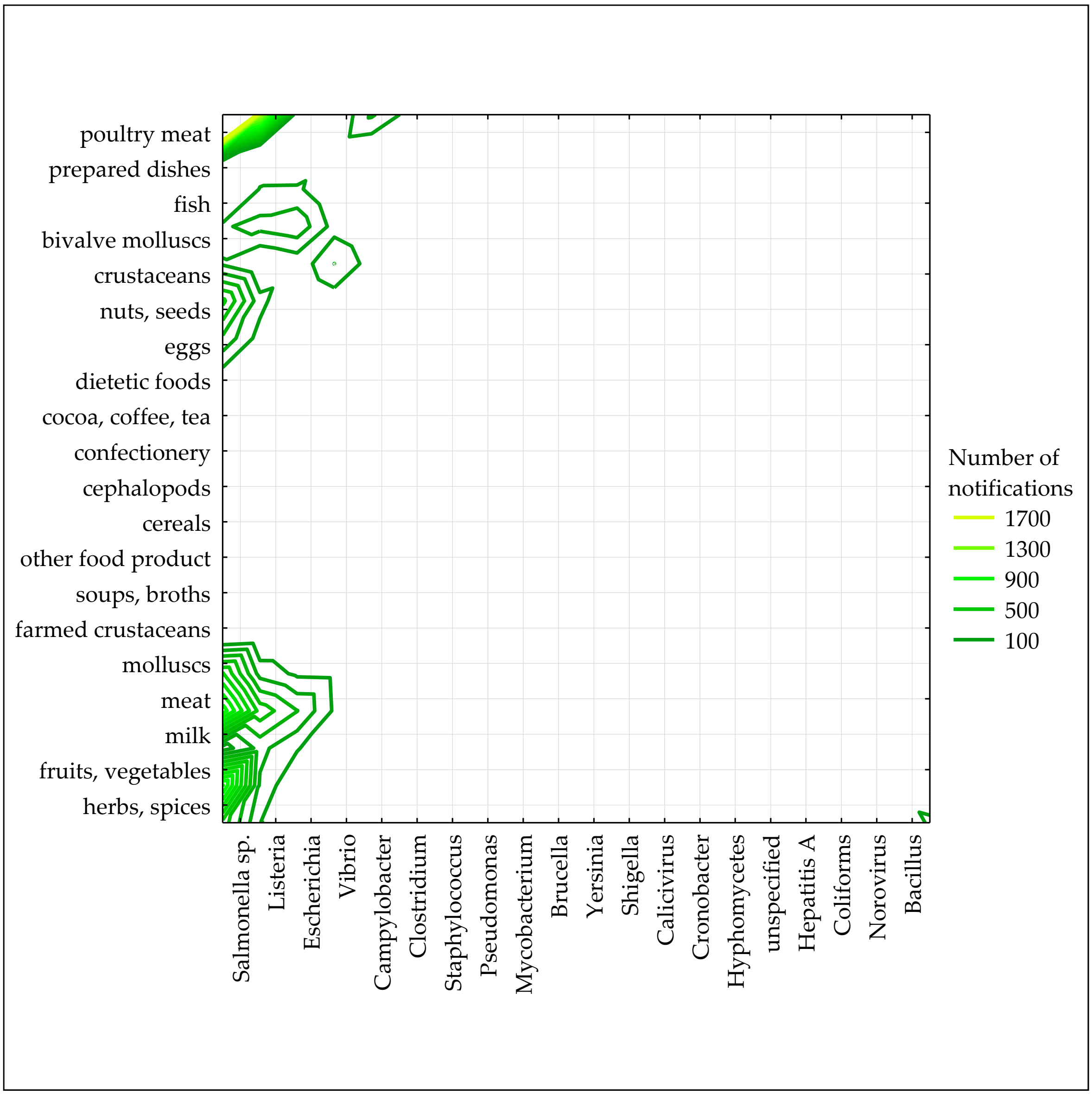

Figure S20. Similarities of RASFF notifications on pathogenic microorganisms and product category within food using two-way joining.

bivalve molluscs – bivalve molluscs and products thereof
cephalopods – cephalopods and products thereof
cereals – cereals and bakery products
cocoa, coffee, tea – cocoa and cocoa preparations, coffee and tea
crustaceans – crustaceans and products thereof
dietetic foods – dietetic foods, food supplements, fortified foods
eggs – eggs and egg products
farmed crustaceans – farmed crustaceans and products thereof - (obsolete)
fish – fish and fish products
fruits, vegetables – fruits and vegetables
herbs, spices – herbs and spices
meat – meat and meat products (other than poultry)
milk – milk and milk products
molluscs – molluscs and products thereof - (obsolete)
nuts, seeds – nuts, nut products and seeds
other food product – other food product / mixed
poultry meat – poultry meat and poultry meat products
prepared dishes – prepared dishes and snacks
soups, broths – soups, broths, sauces and condiments

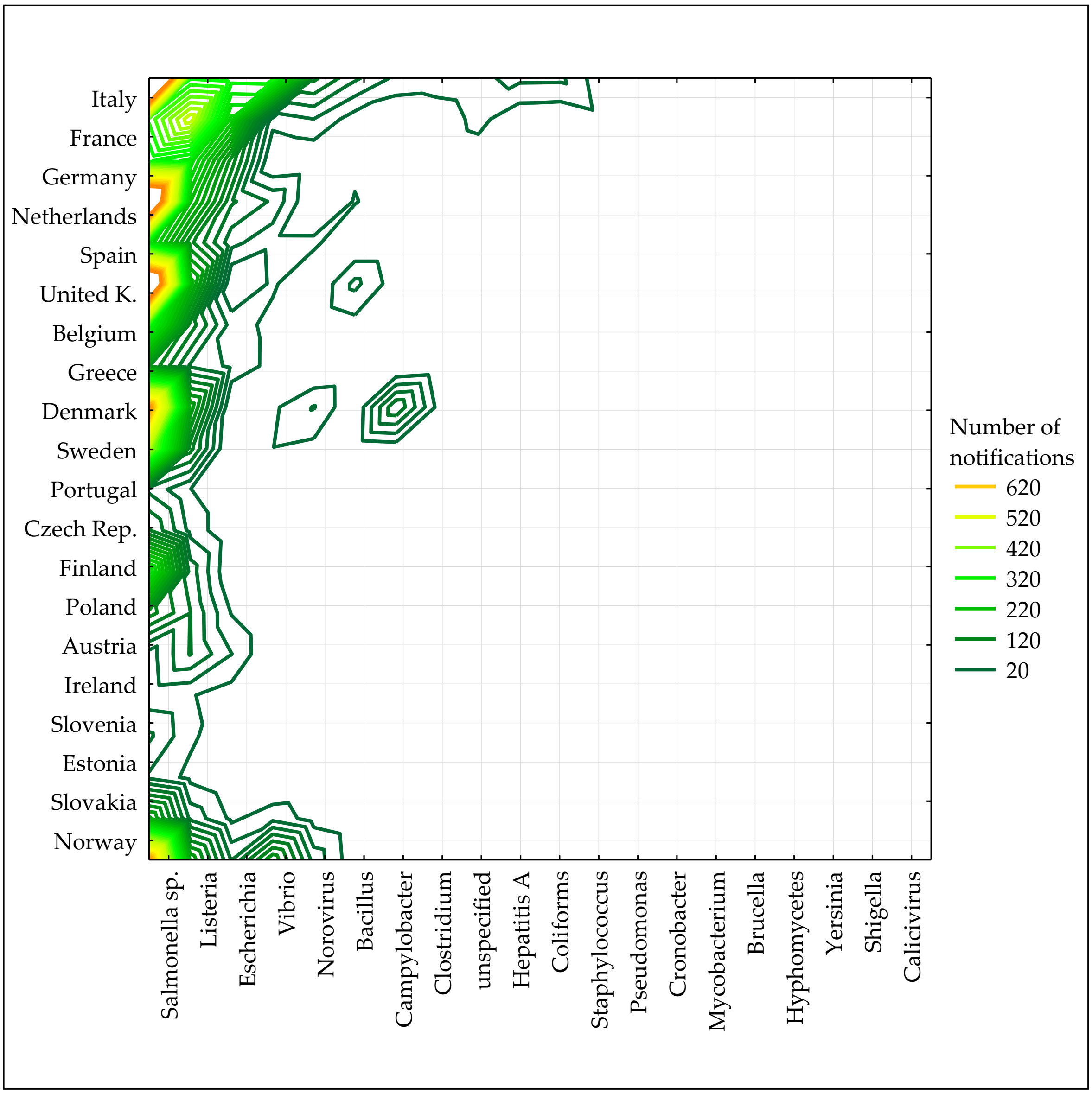

Figure S21. Similarities of RASFF notifications on pathogenic microorganisms and notifying country within food using two-way joining.

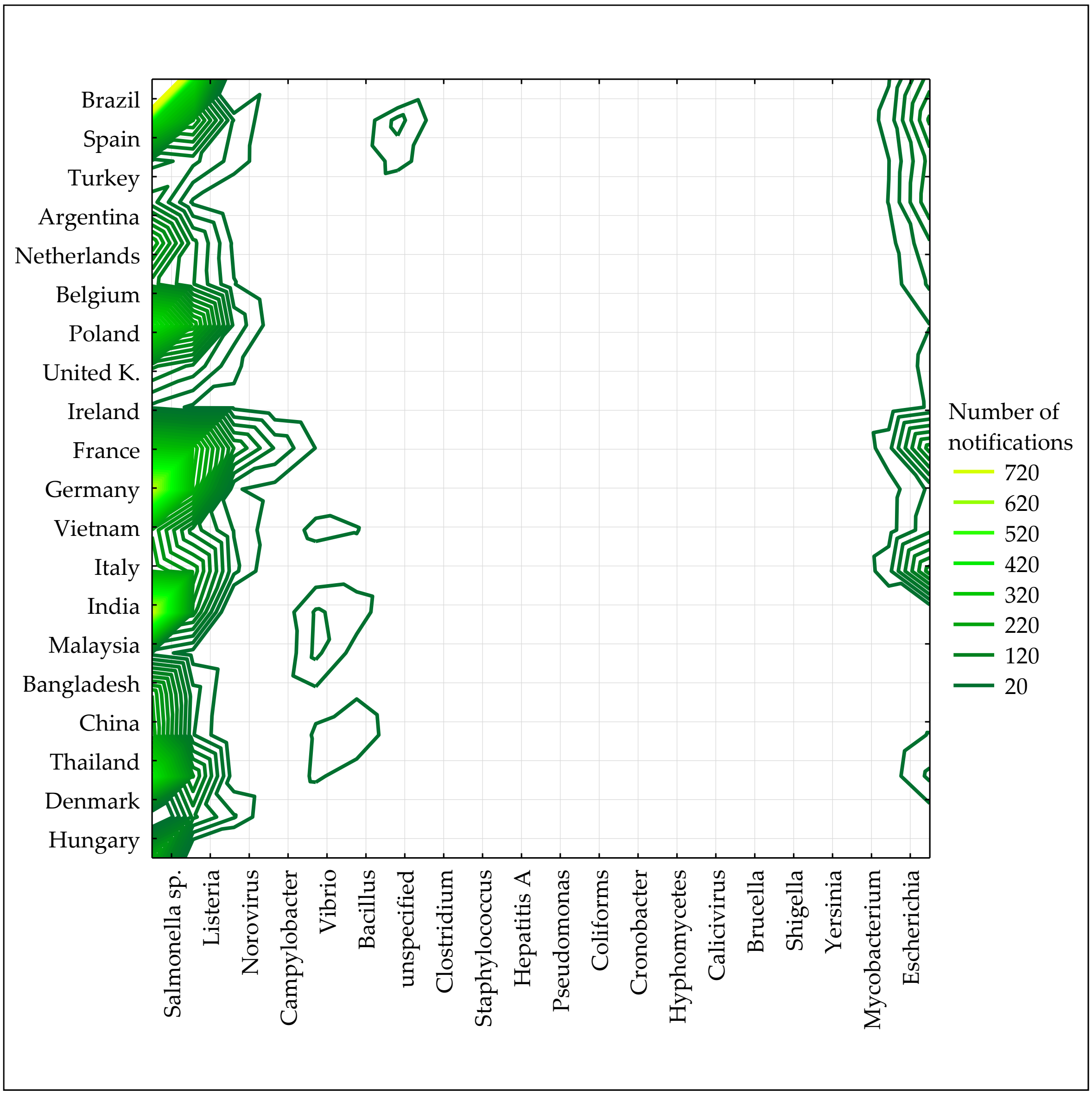

Figure S22. Similarities of RASFF notifications on pathogenic microorganisms and origin country within food using two-way joining.

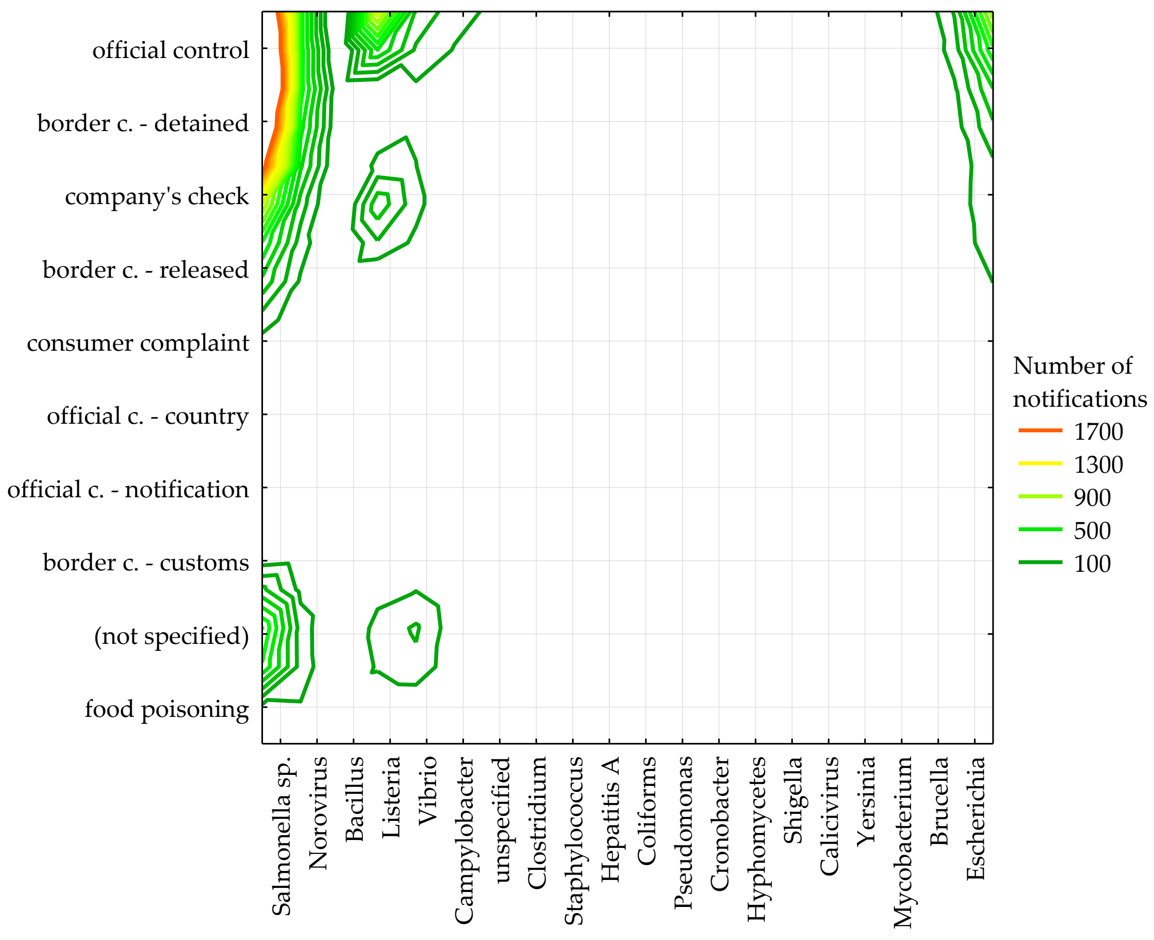

Figure S23. Similarities of RASFF notifications on pathogenic microorganisms and notification basis within food using two-way joining.

border c. - customs – border control - consignment under customs
border c. - detained – border control - consignment detained
border c. - released – border control - consignment released
company's check – company's own check
official c. - country – official control in non-member country
official c. - notification – official control following RASFF notification
official control – official control on the market

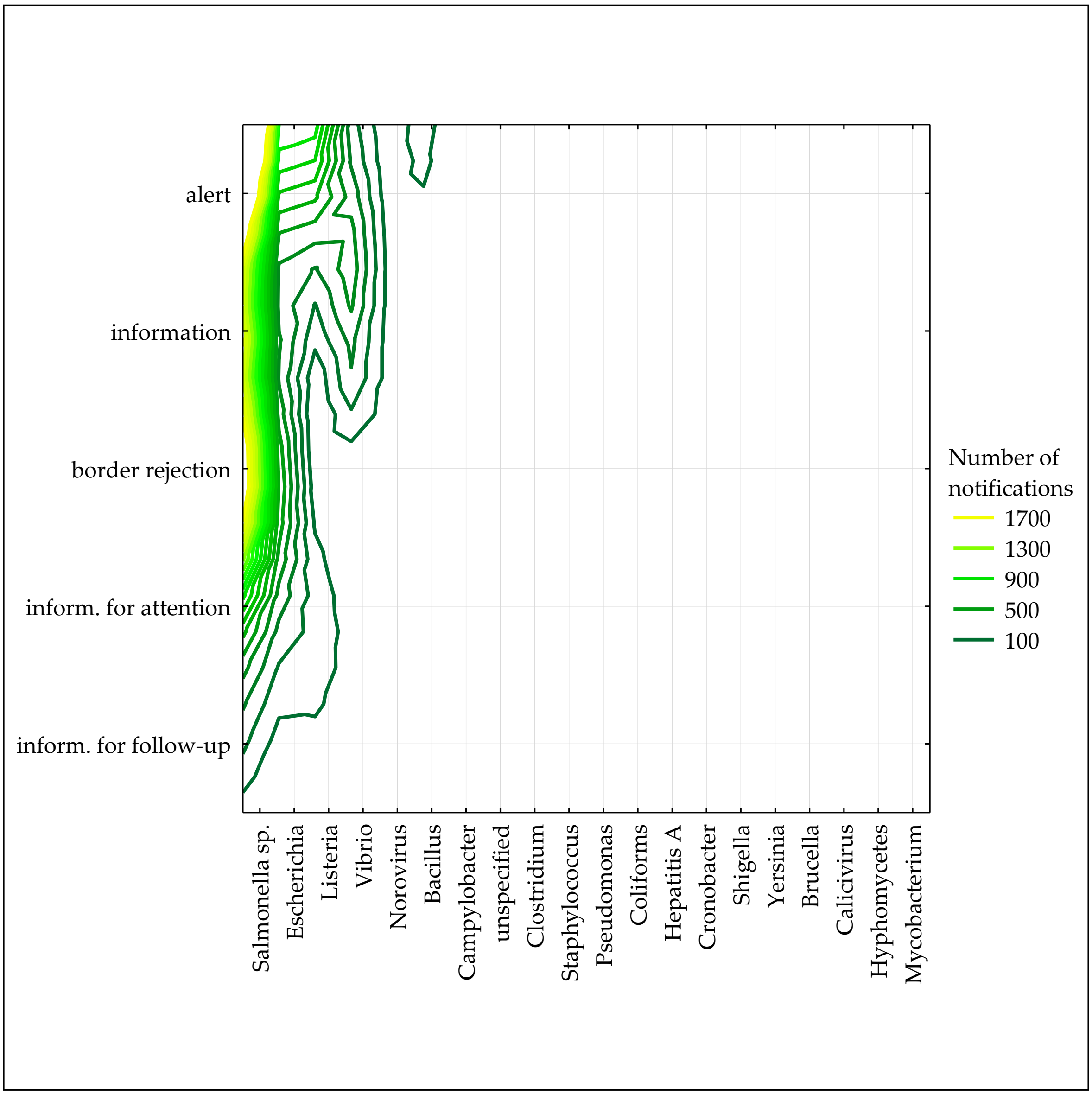

Figure S24. Similarities of RASFF notifications on pathogenic microorganisms and notification type within food using two-way joining.

inform. for attention – information for attention
inform. for follow-up – information for follow-up

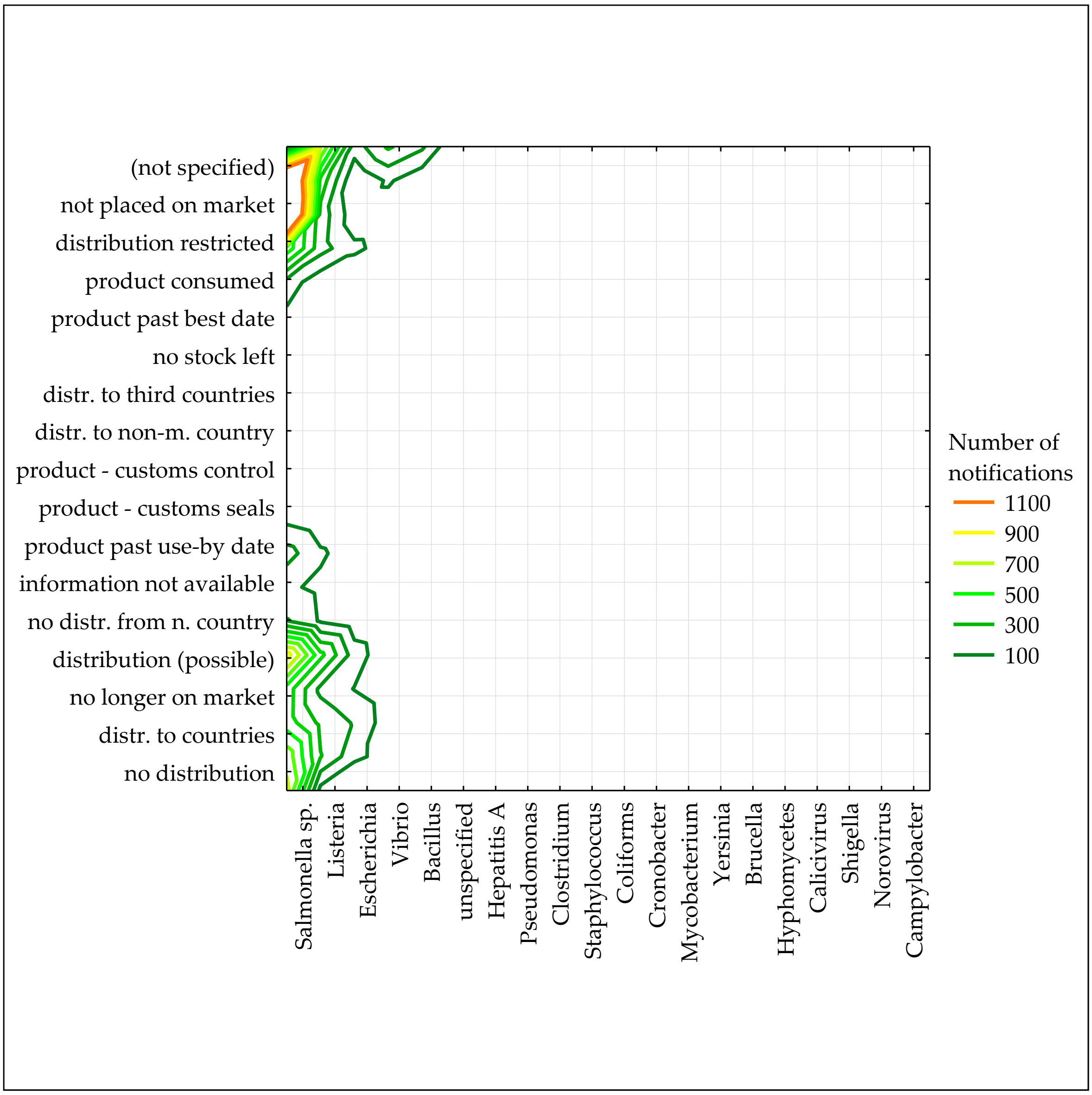

Figure S25. Similarities of RASFF notifications on pathogenic microorganisms and distribution status within food using two-way joining.

distr. to non-m. countries – distribution to non-member countries
distr. to countries – distribution to other member countries
distr. to third countries – distribution to third countries
distribution (possible) – distribution on the market (possible)
distribution restricted – distribution restricted to notifying country
information not available – information on distribution not (yet) available
no distr. from n. country – no distribution from notifying country
no longer on market – product (presumably) no longer on the market
not placed on market – product not (yet) placed on the market
product - customs control – product under customs control
product - customs seals – product allowed to travel to destination under customs seals
product consumed – product already consumed
product past best date – product past best before date

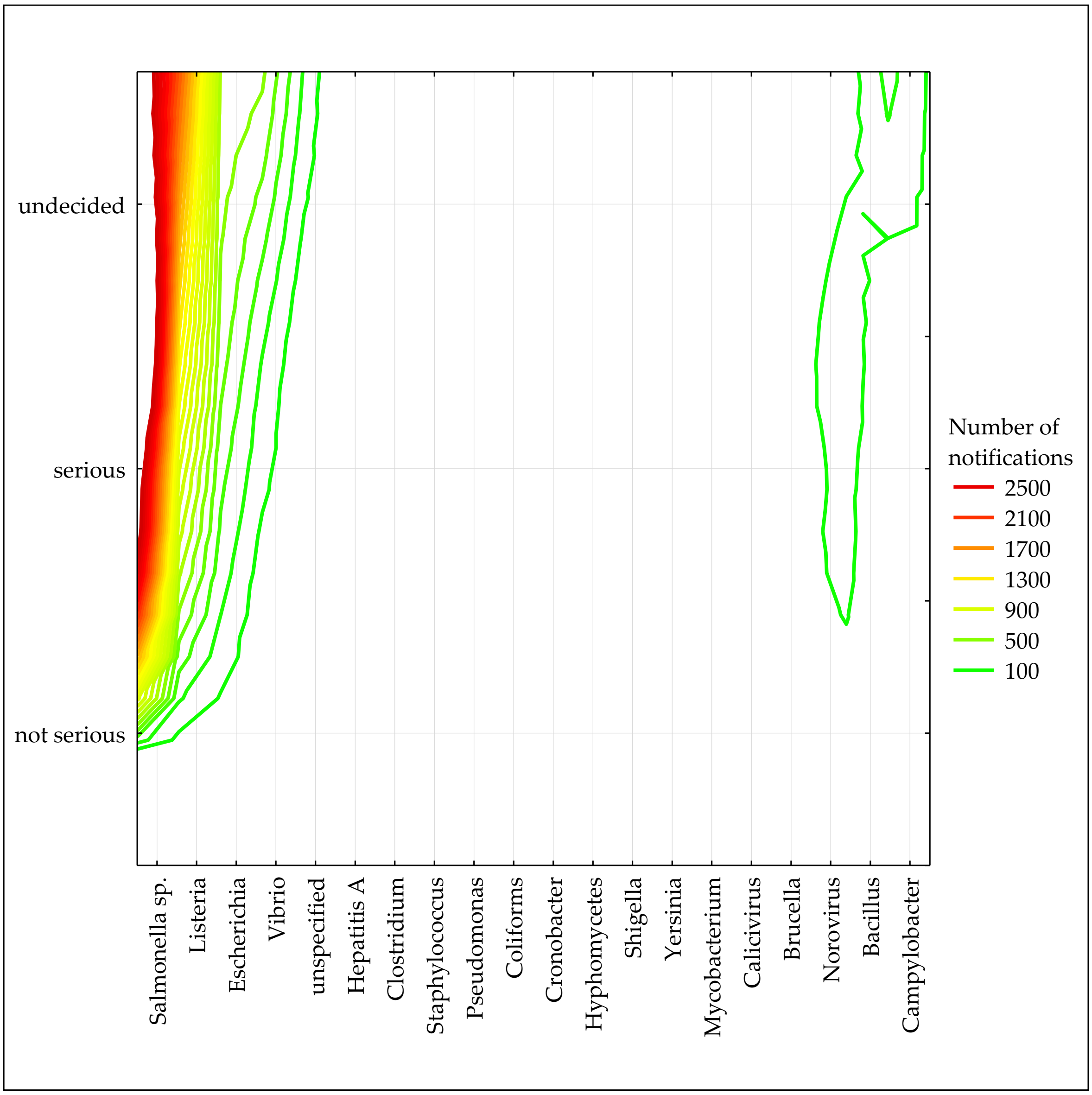

Figure S26. Similarities of RASFF notifications on pathogenic microorganisms and risk decision within food using two-way joining.

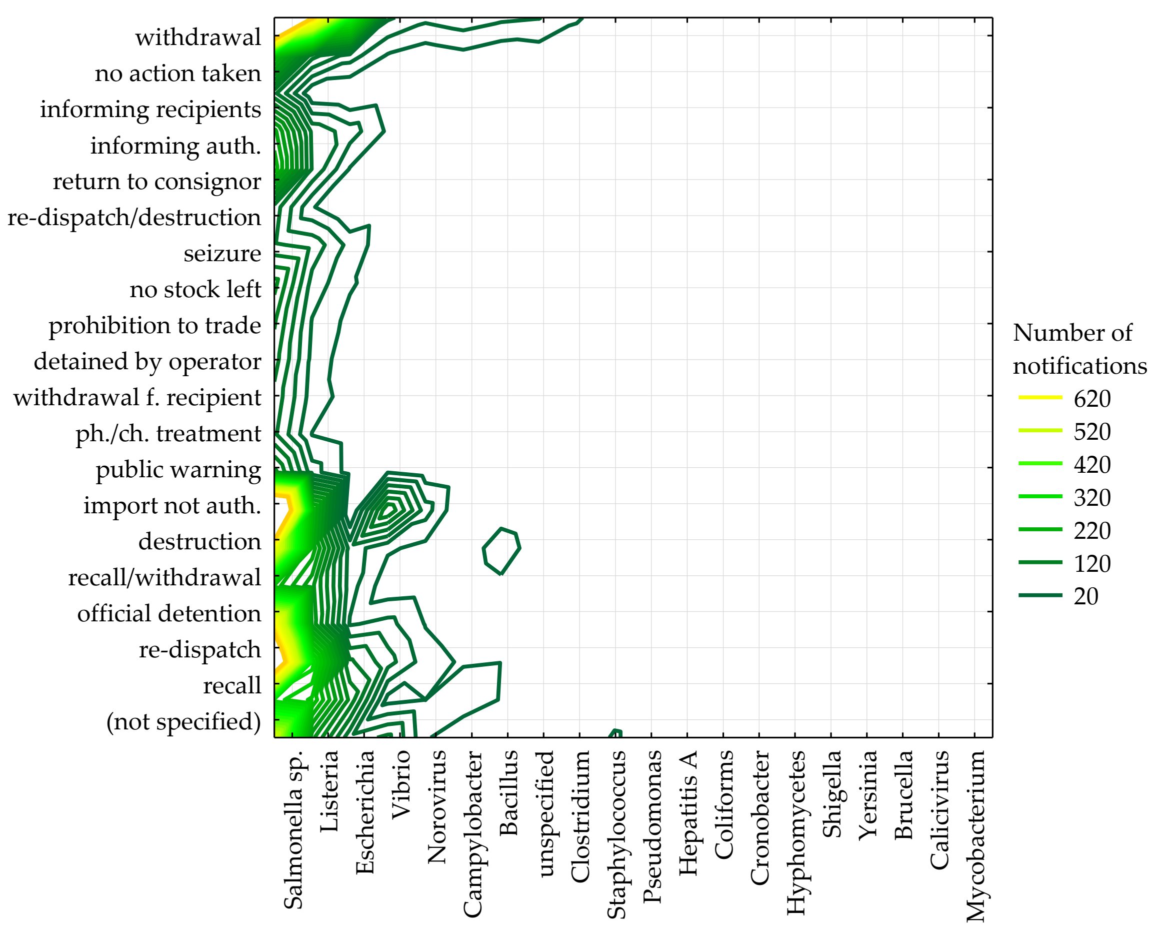

Figure S27. Similarities of RASFF notifications on pathogenic microorganisms and action taken within food using two-way joining.

import not auth. – import not authorised
informing auth. – informing authorities
ph./ch. treatment – physical/chemical treatment
prohibition to trade – prohibition to trade - sales ban
public warning – public warning - press release
recall – recall from consumers
recall/withdrawal – product recall or withdrawal
re-dispatch/destruction – re-dispatch or destruction
withdrawal – withdrawal from the market
withdrawal f. recipient – withdrawal from recipient(s)

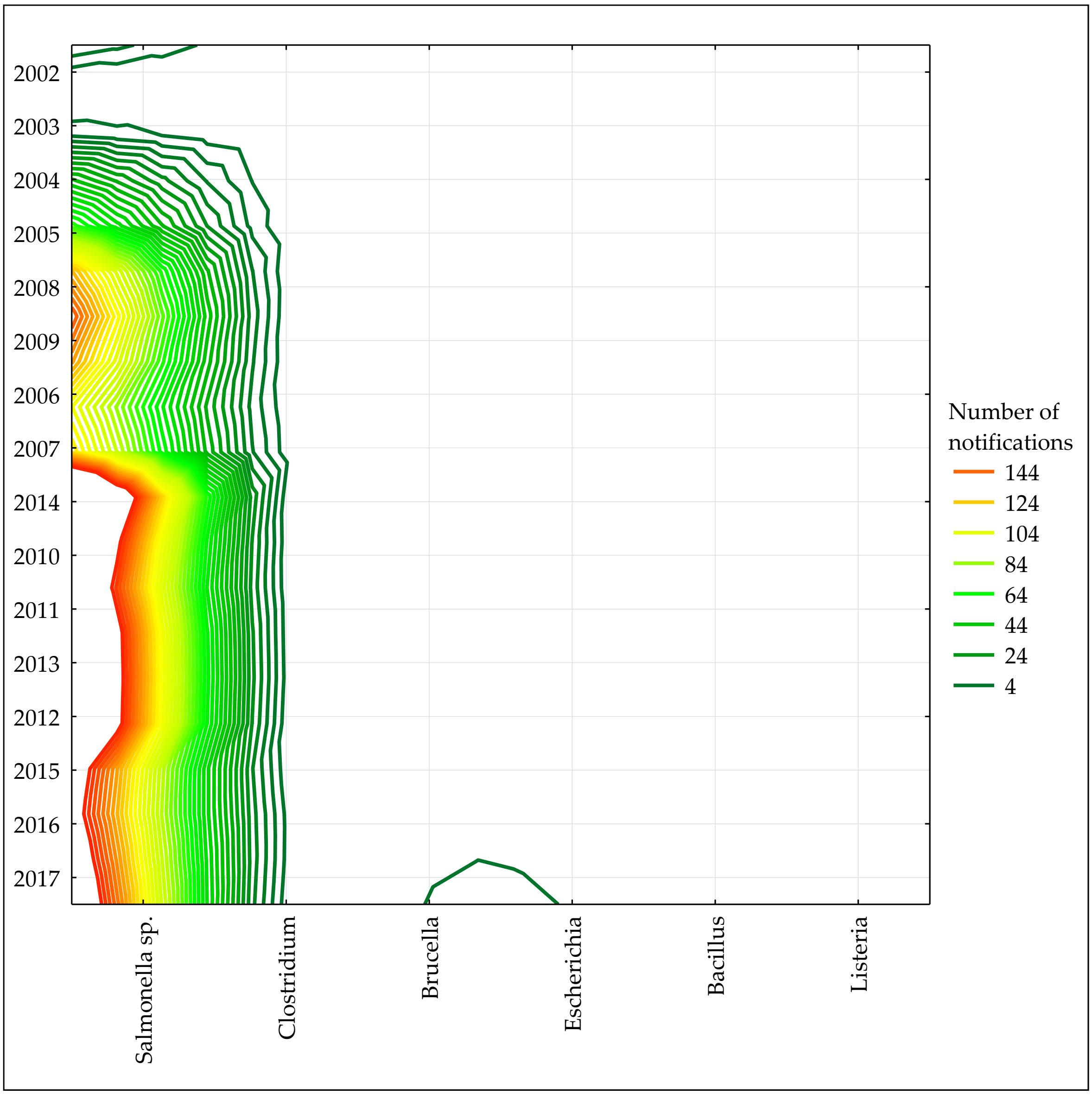

Figure S28. Similarities of RASFF notifications on pathogenic microorganisms and year within feed using two-way joining.

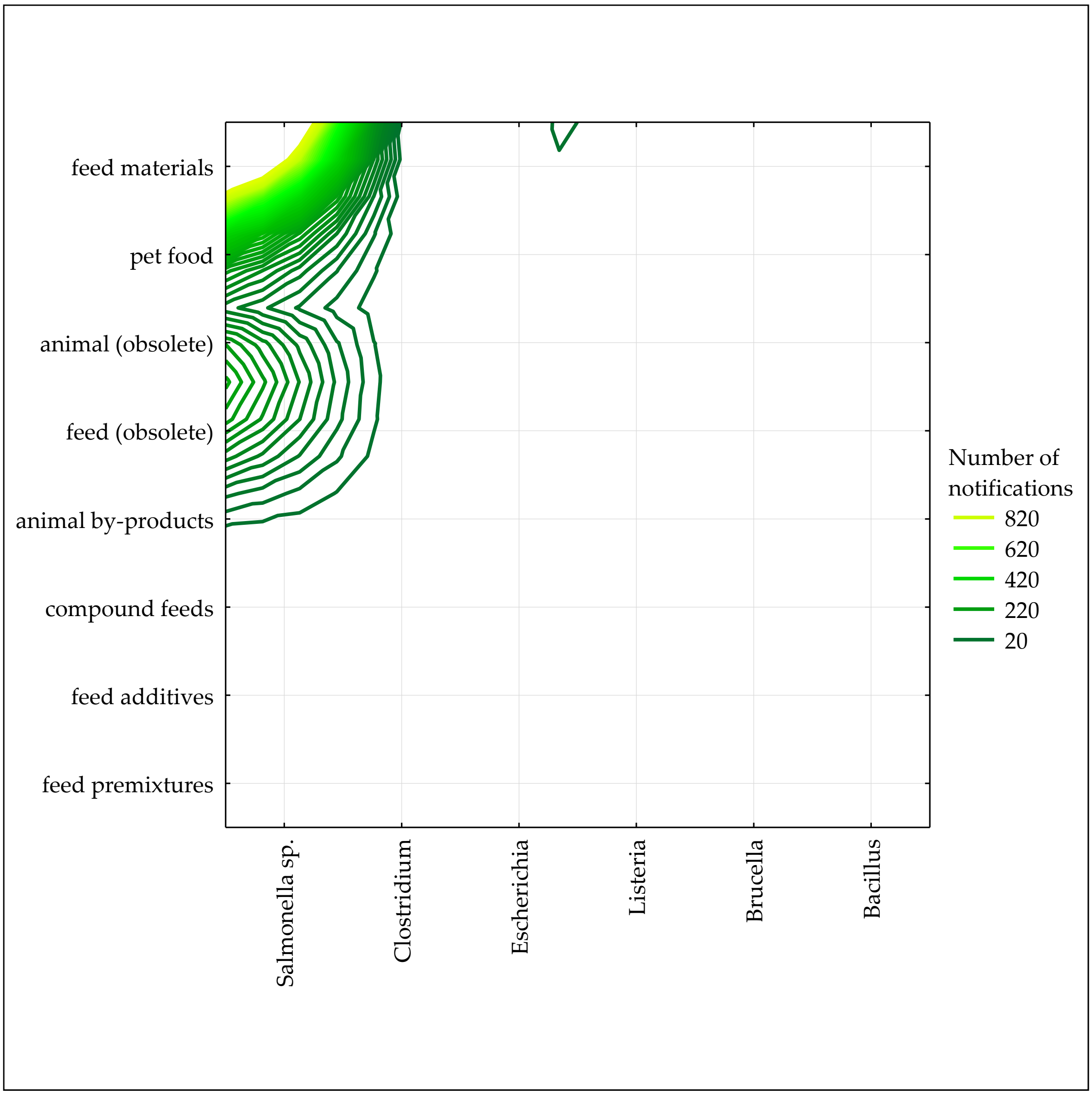

Figure S29. Similarities of RASFF notifications on pathogenic microorganisms and product category within feed using two-way joining.

animal (obsolete) – animal nutrition - (obsolete)
feed (obsolete) – feed for food-producing animals - (obsolete)

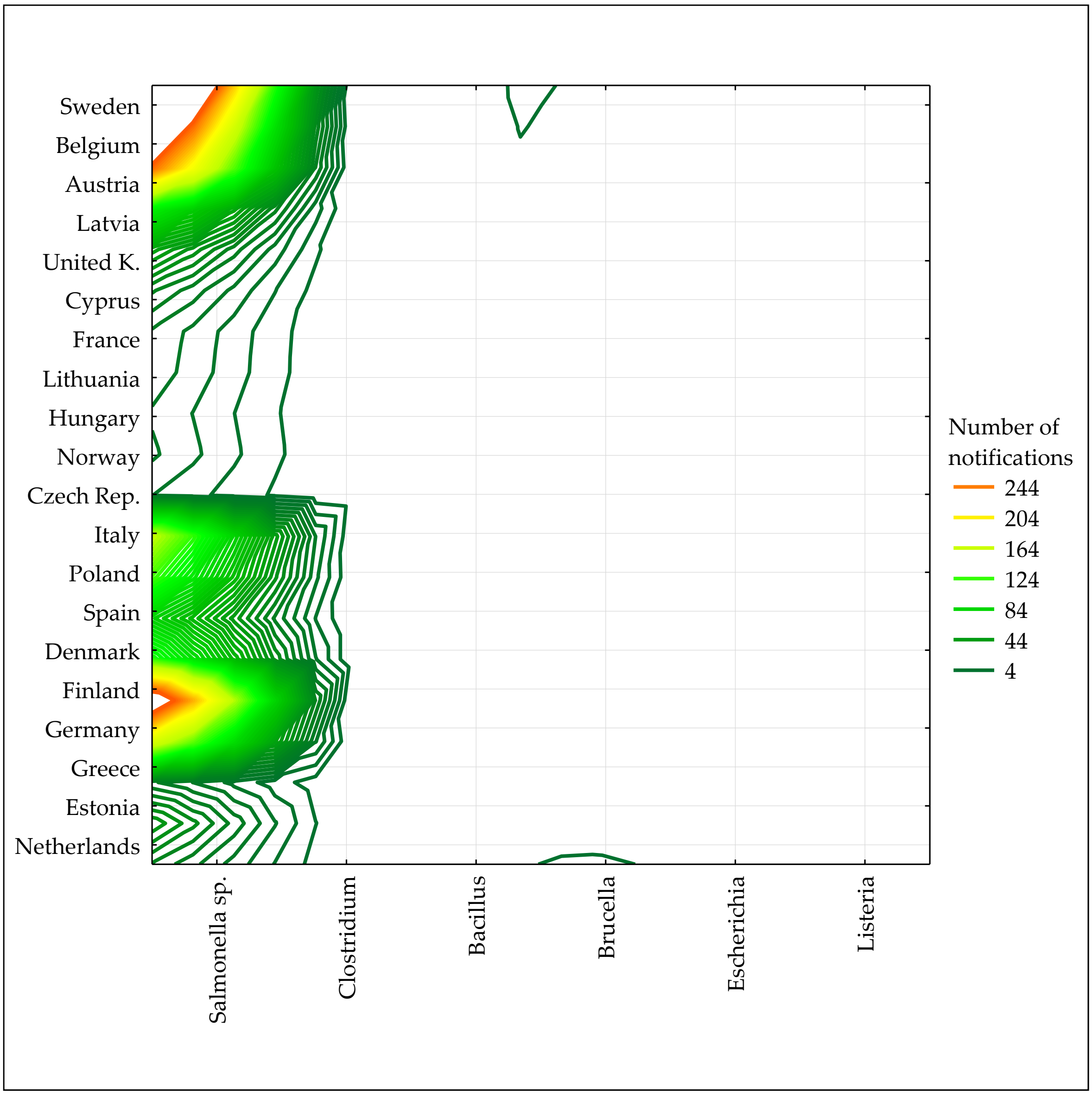

Figure S30. Similarities of RASFF notifications on pathogenic microorganisms and notifying country within feed using two-way joining.

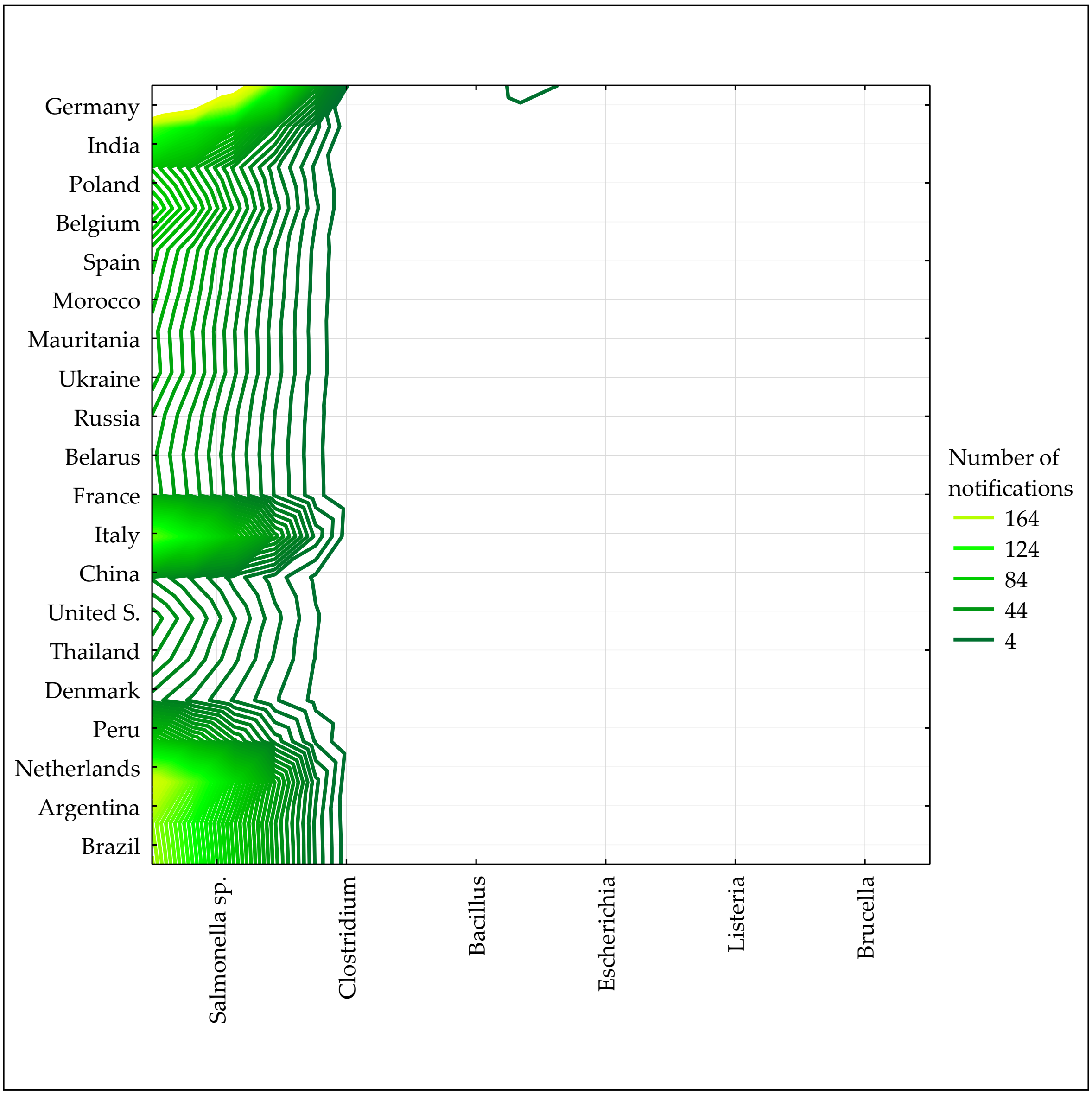

Figure S31. Similarities of RASFF notifications on pathogenic microorganisms and origin country within feed using two-way joining.

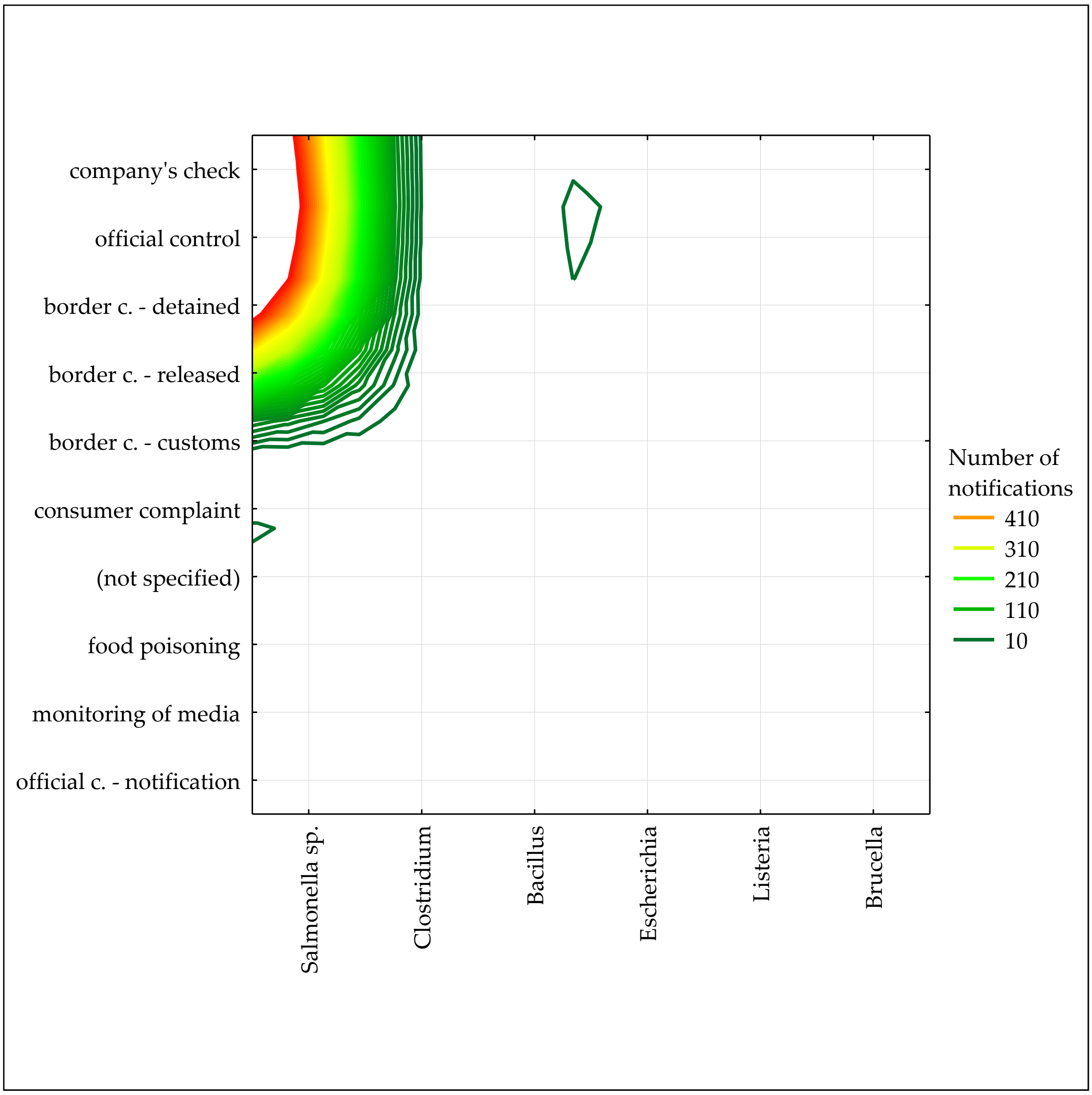

Figure S32. Similarities of RASFF notifications on pathogenic microorganisms and notification basis within feed using two-way joining.

border c. - customs – border control - consignment under customs
border c. - detained – border control - consignment detained
border c. - released – border control - consignment released
company's check – company's own check
official c. - notification – official control following RASFF notification
official control – official control on the market

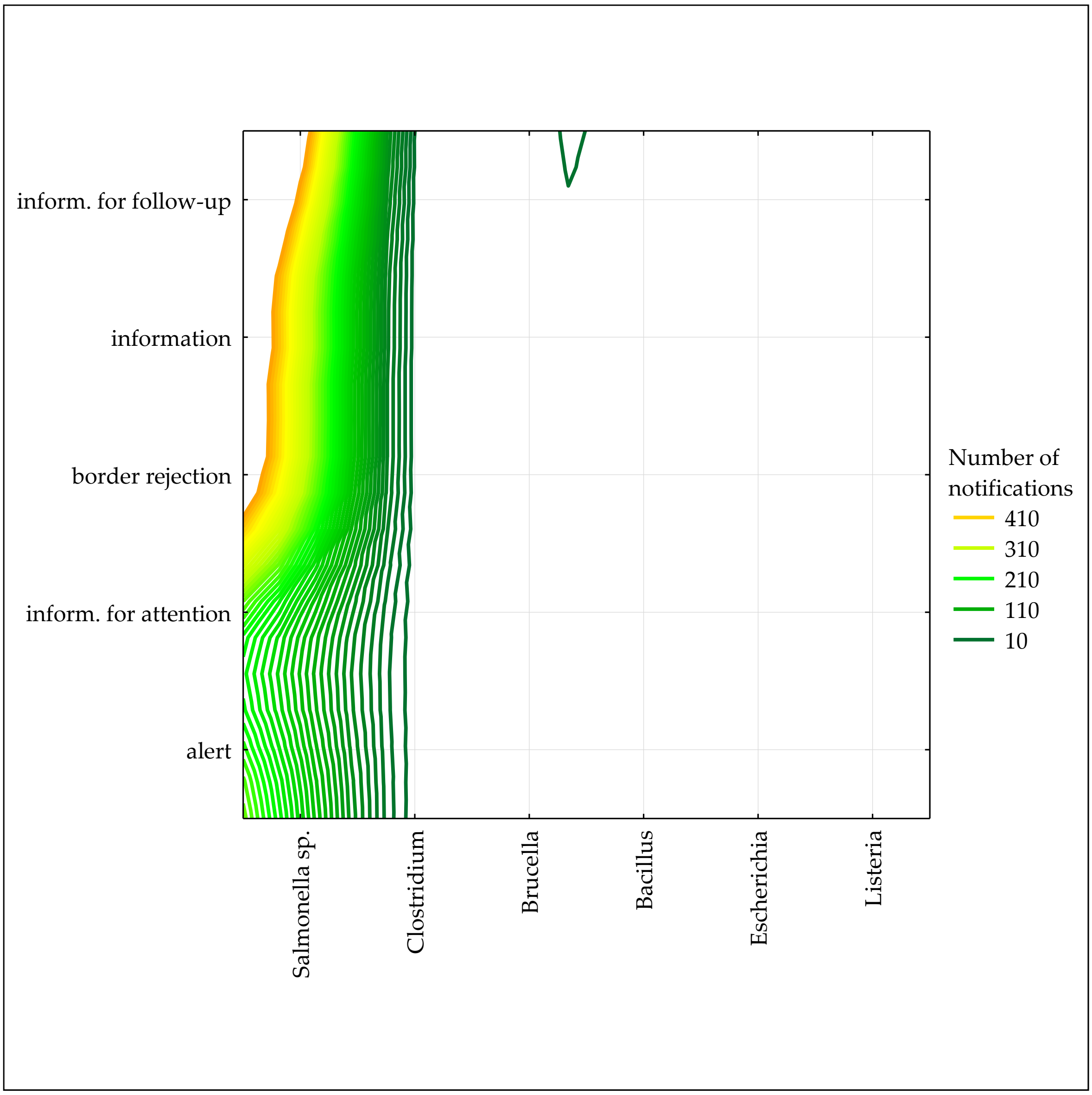

Figure S33. Similarities of RASFF notifications on pathogenic microorganisms and notification type within feed using two-way joining.

inform. for attention – information for attention
inform. for follow-up – information for follow-up

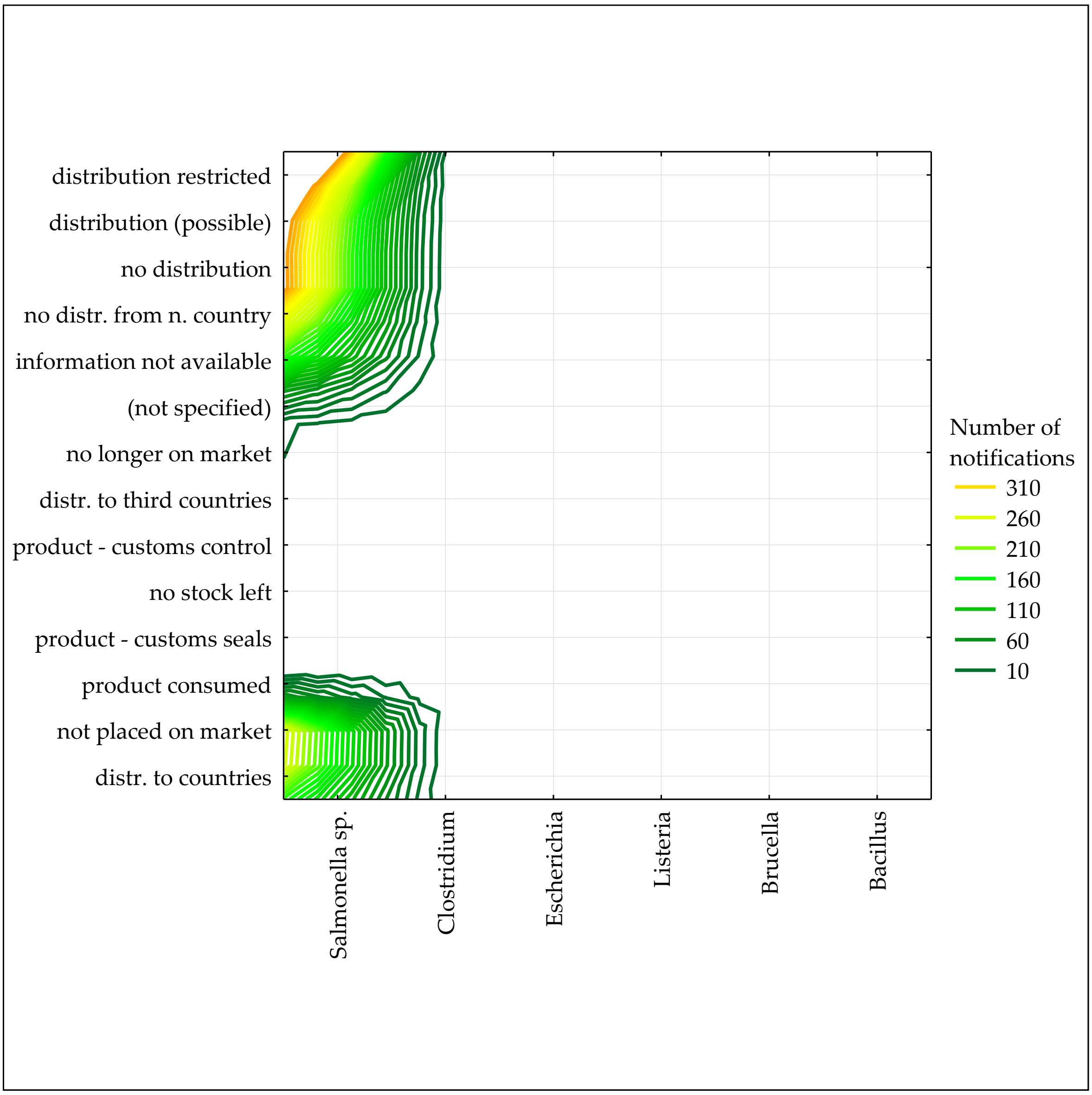

Figure S34. Similarities of RASFF notifications on pathogenic microorganisms and distribution status within feed using two-way joining.

distr. to countries – distribution to other member countries
distr. to third countries – distribution to third countries
distribution (possible) – distribution on the market (possible)
distribution restricted – distribution restricted to notifying country
information not available – information on distribution not (yet) available
no distr. from n. country – no distribution from notifying country
no longer on market – product (presumably) no longer on the market
not placed on market – product not (yet) placed on the market
product - customs control – product under customs control
product - customs seals – product allowed to travel to destination under customs seals
product consumed – product already consumed

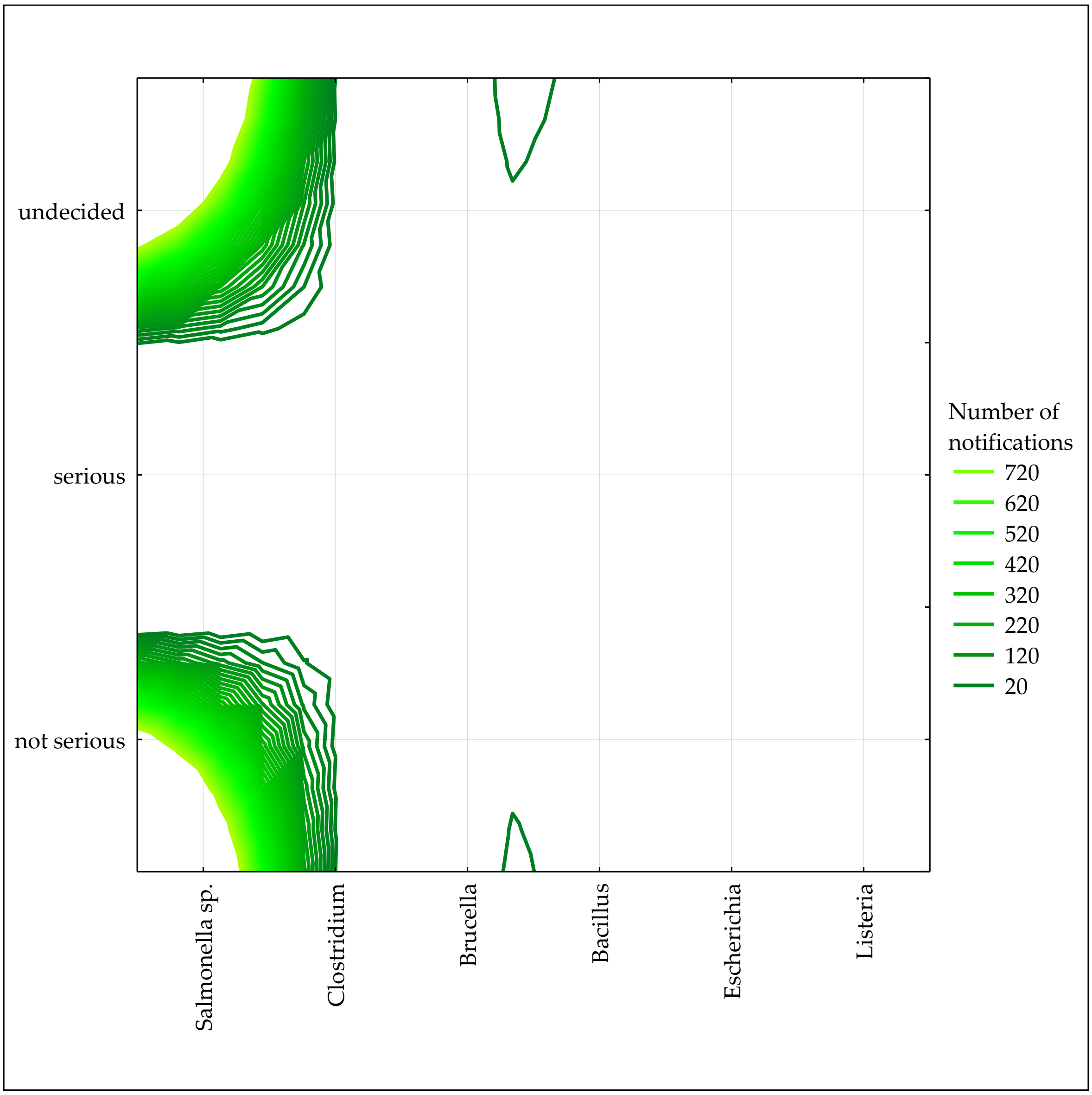

Figure S35. Similarities of RASFF notifications on pathogenic microorganisms and risk decision within feed using two-way joining.

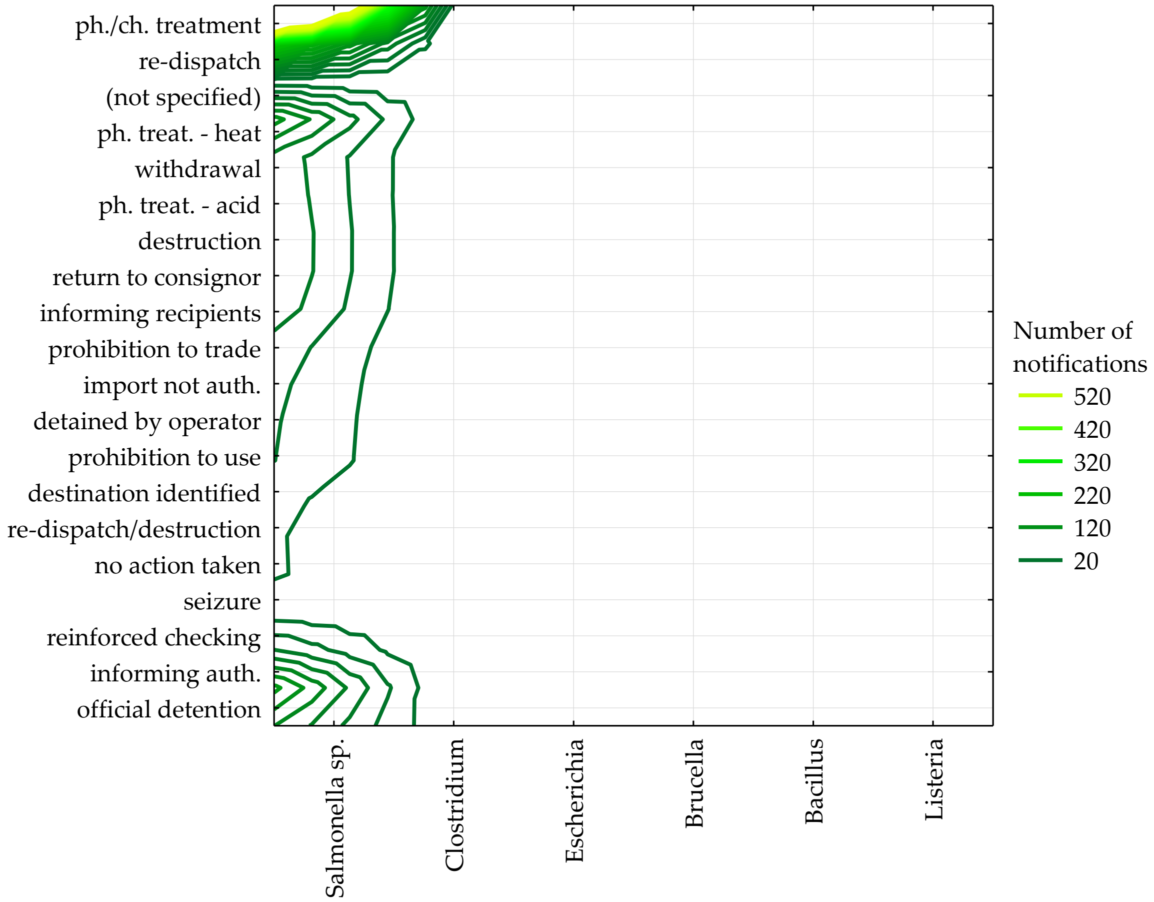

Figure S36. Similarities of RASFF notifications on pathogenic microorganisms and action taken within feed using two-way joining.

destination identified – destination of the product identified
import not auth. – import not authorised
informing auth. – informing authorities
ph. treat. - acid – physical treatment - acid treatment
ph. treat. - heat – physical treatment - heat treatment
ph./ch. treatment – physical/chemical treatment
prohibition to trade – prohibition to trade - sales ban
re-dispatch/destruction – re-dispatch or destruction
withdrawal – withdrawal from the market

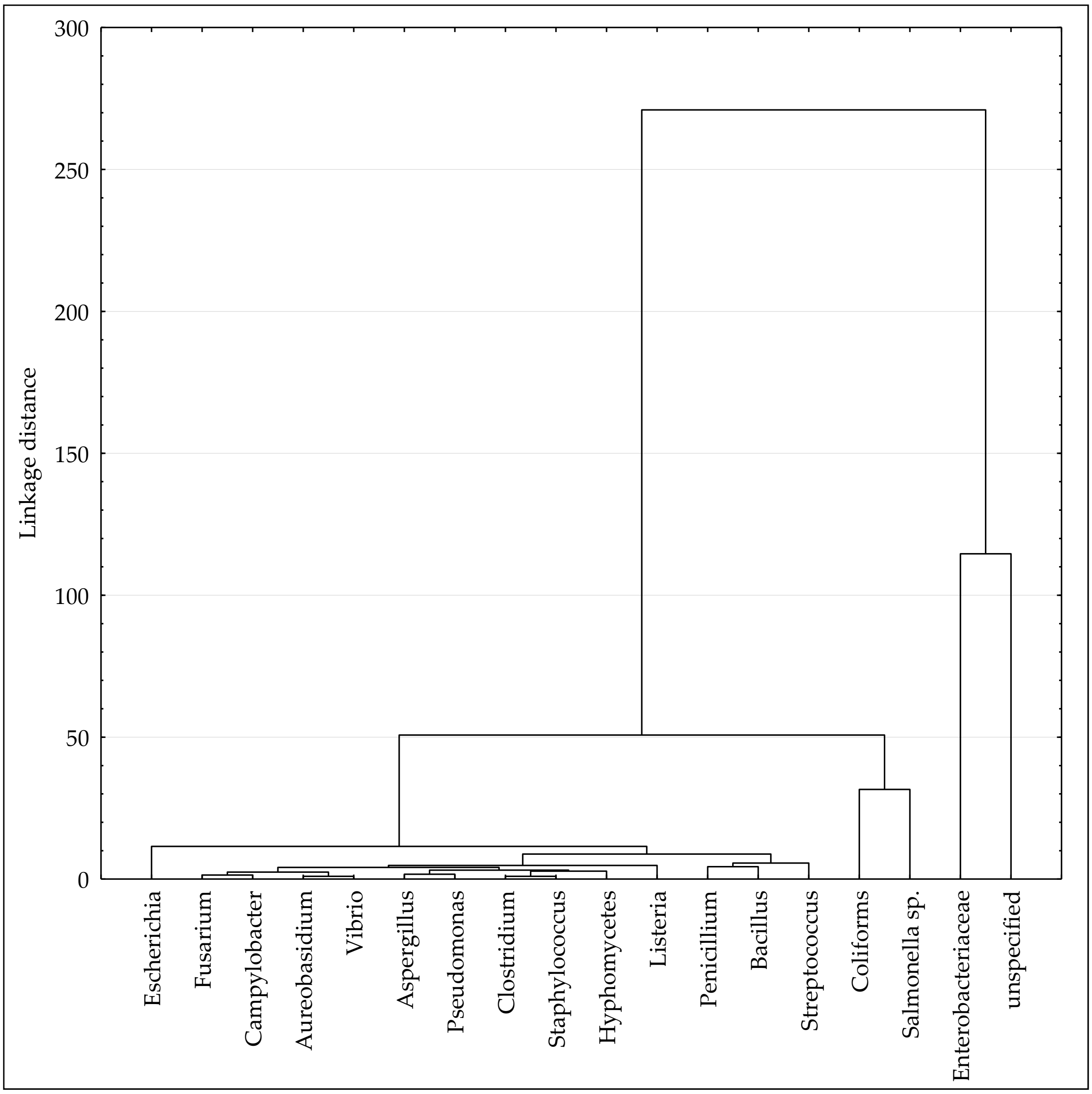

Figure S37. Similarities of RASFF notifications on non-pathogenic microorganisms and year within food using joining.

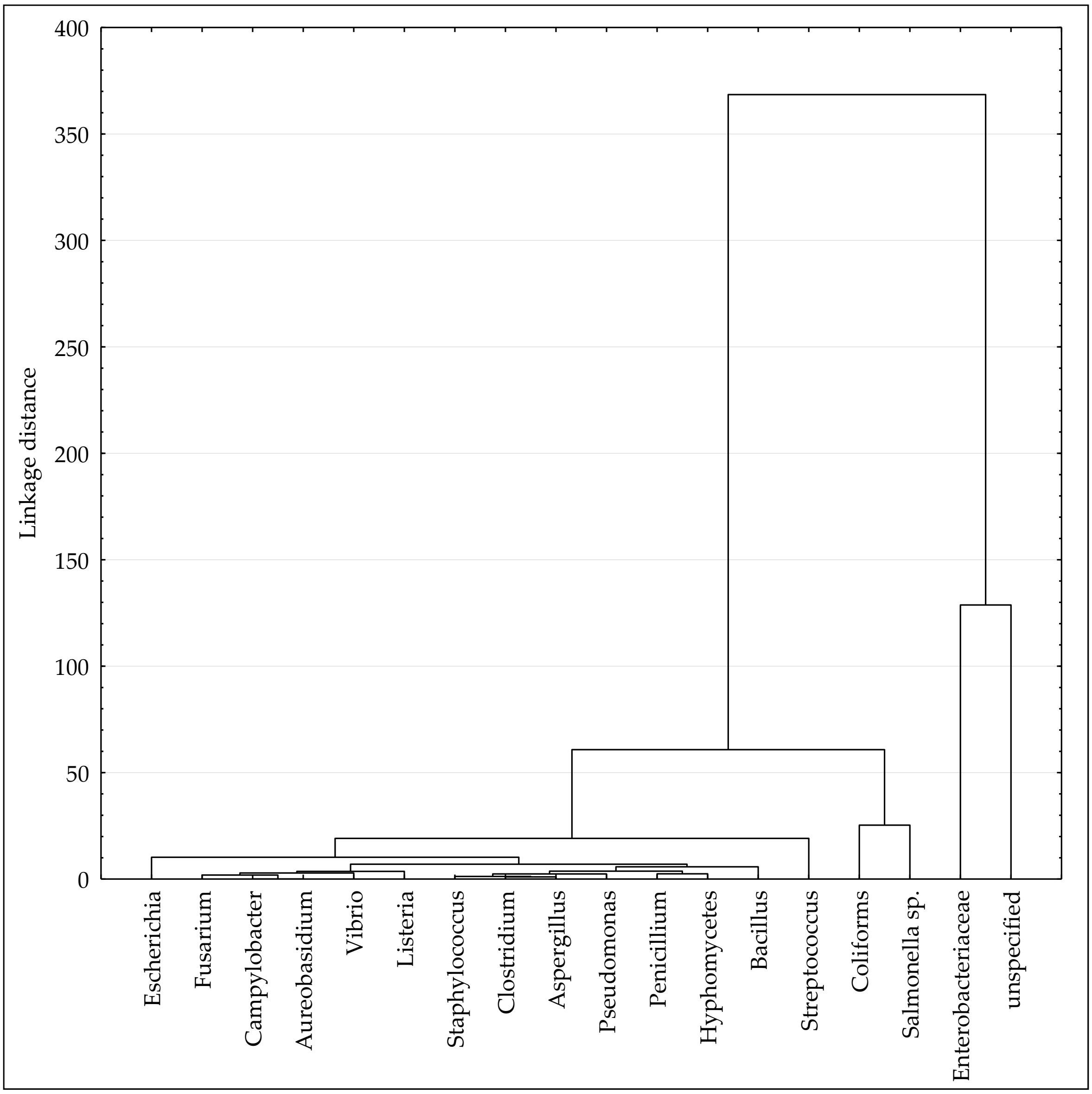

Figure S38. Similarities of RASFF notifications on non-pathogenic microorganisms and product category within food using joining.

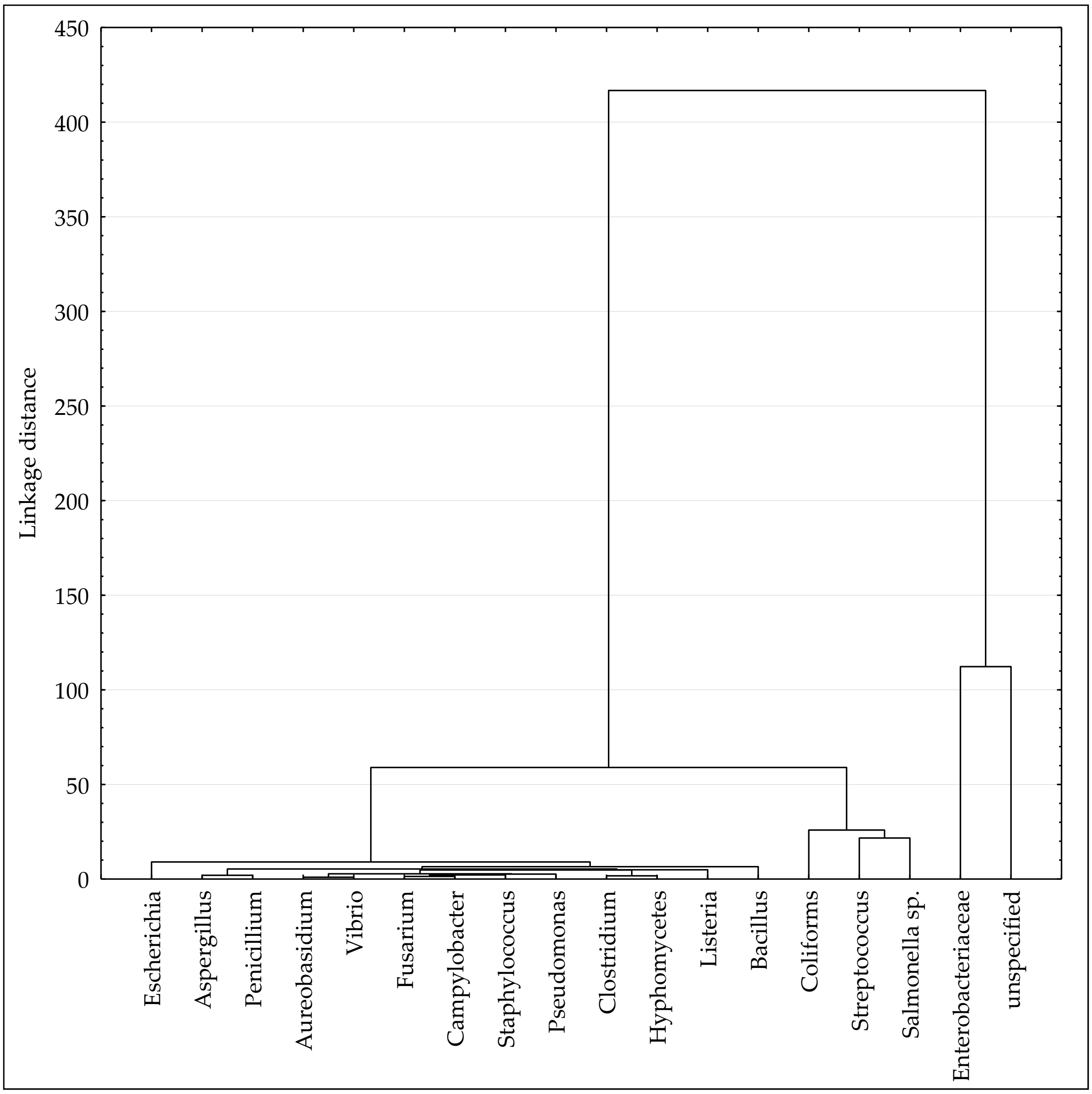

Figure S39. Similarities of RASFF notifications on non-pathogenic microorganisms and notifying country within food using joining.

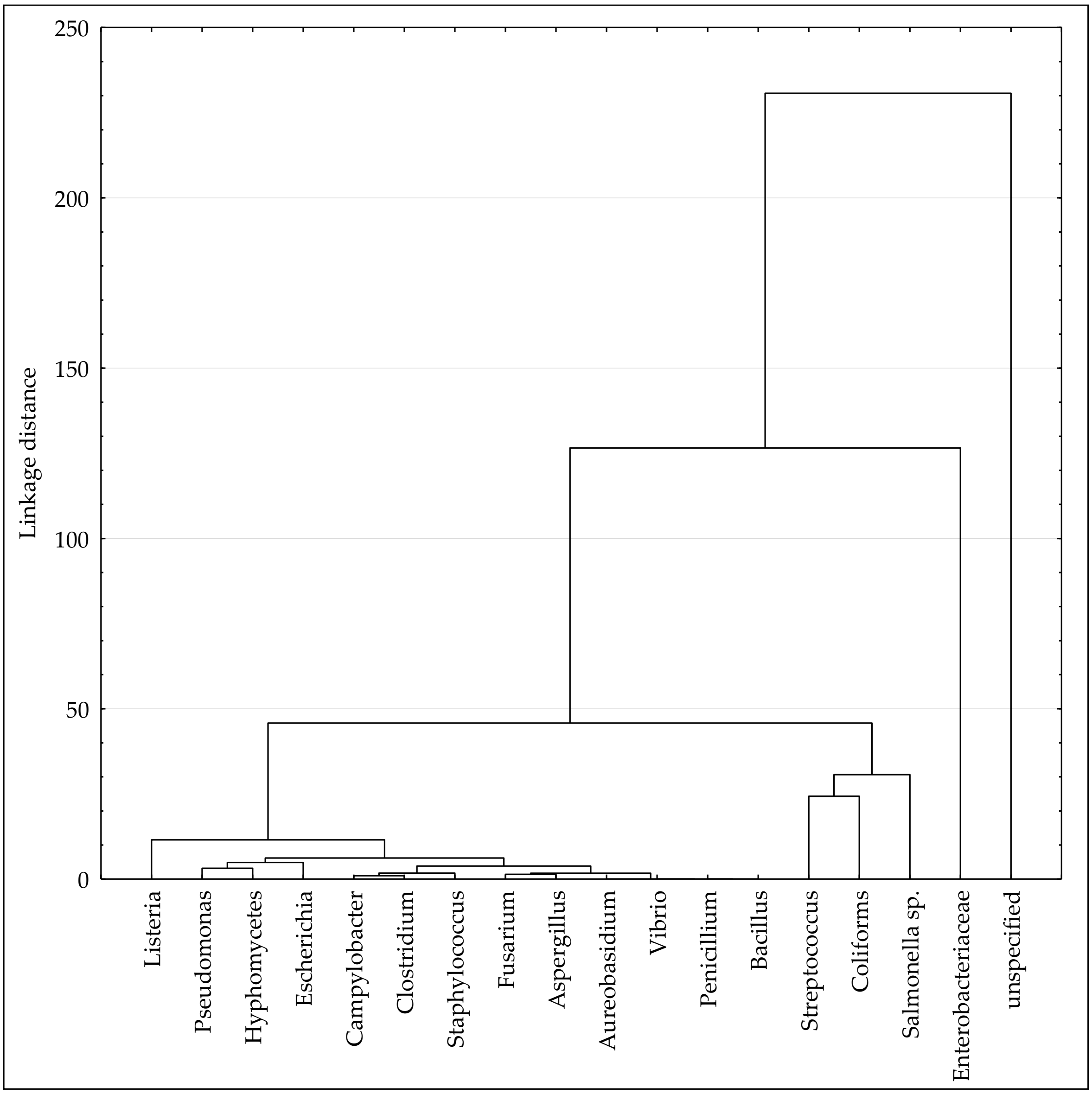

Figure S40. Similarities of RASFF notifications on non-pathogenic microorganisms and origin country within food using joining.

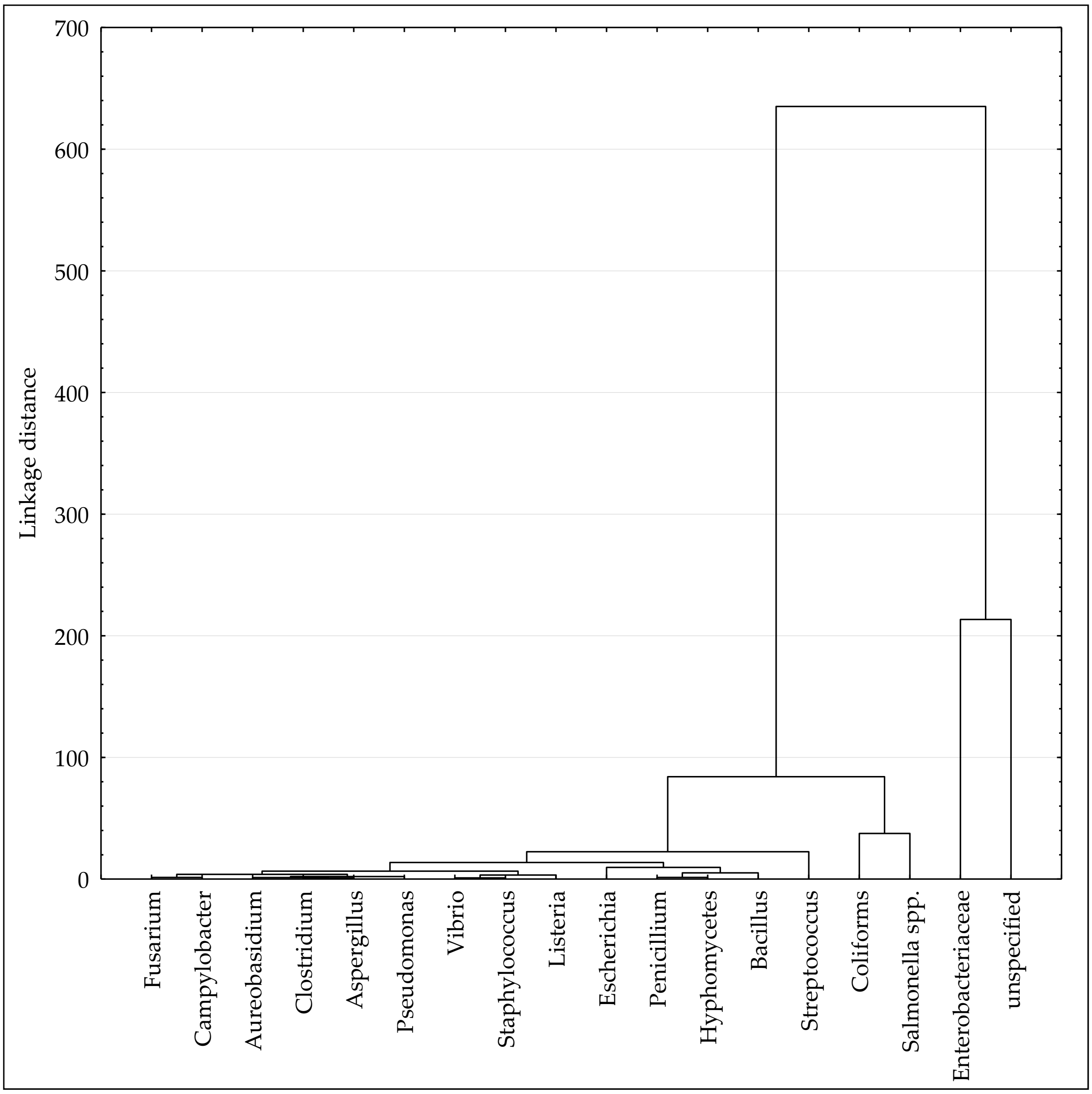

Figure S41. Similarities of RASFF notifications on non-pathogenic microorganisms and notification basis within food using joining.

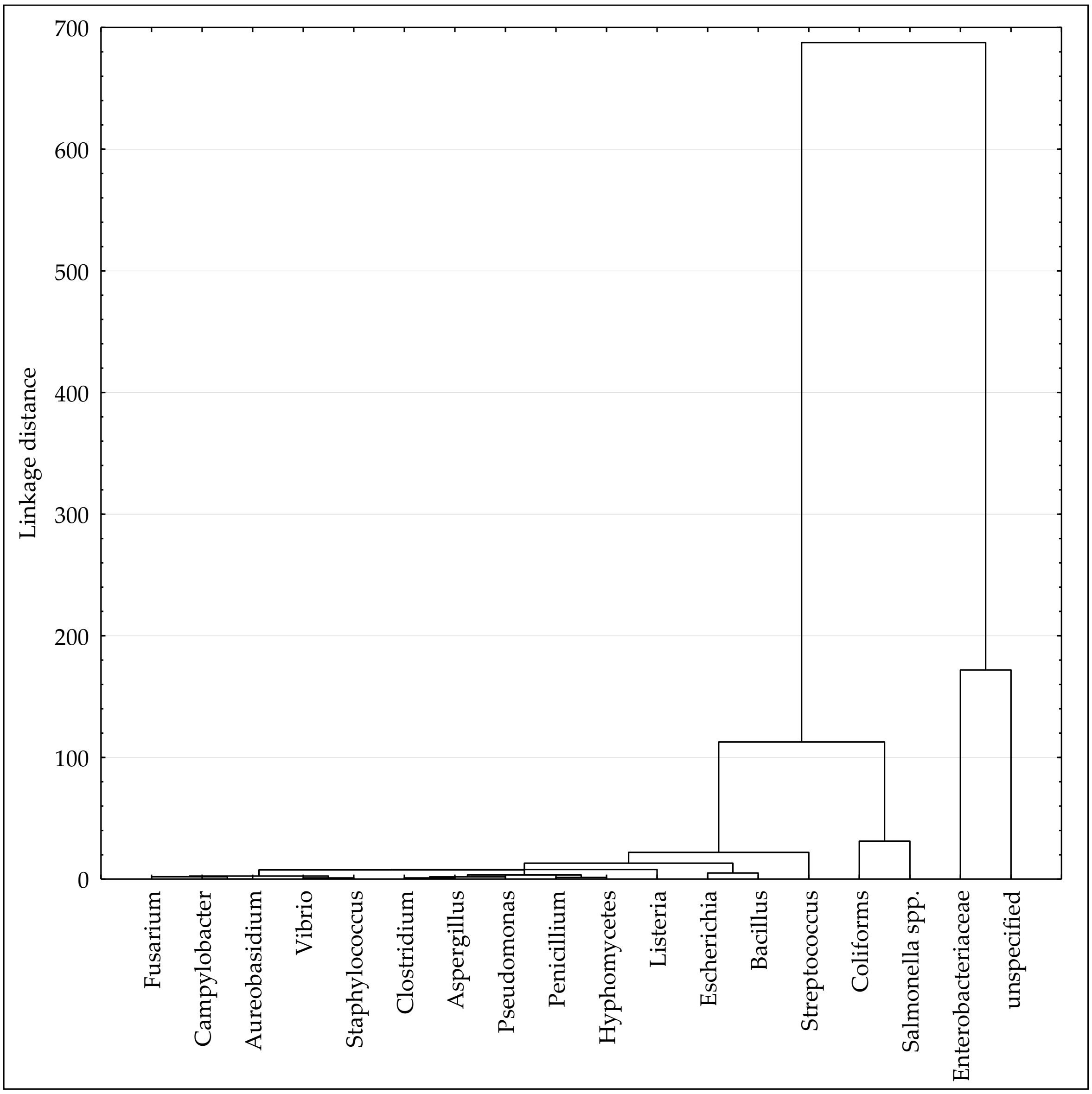

Figure S42. Similarities of RASFF notifications on non-pathogenic microorganisms and notification type within food using joining.

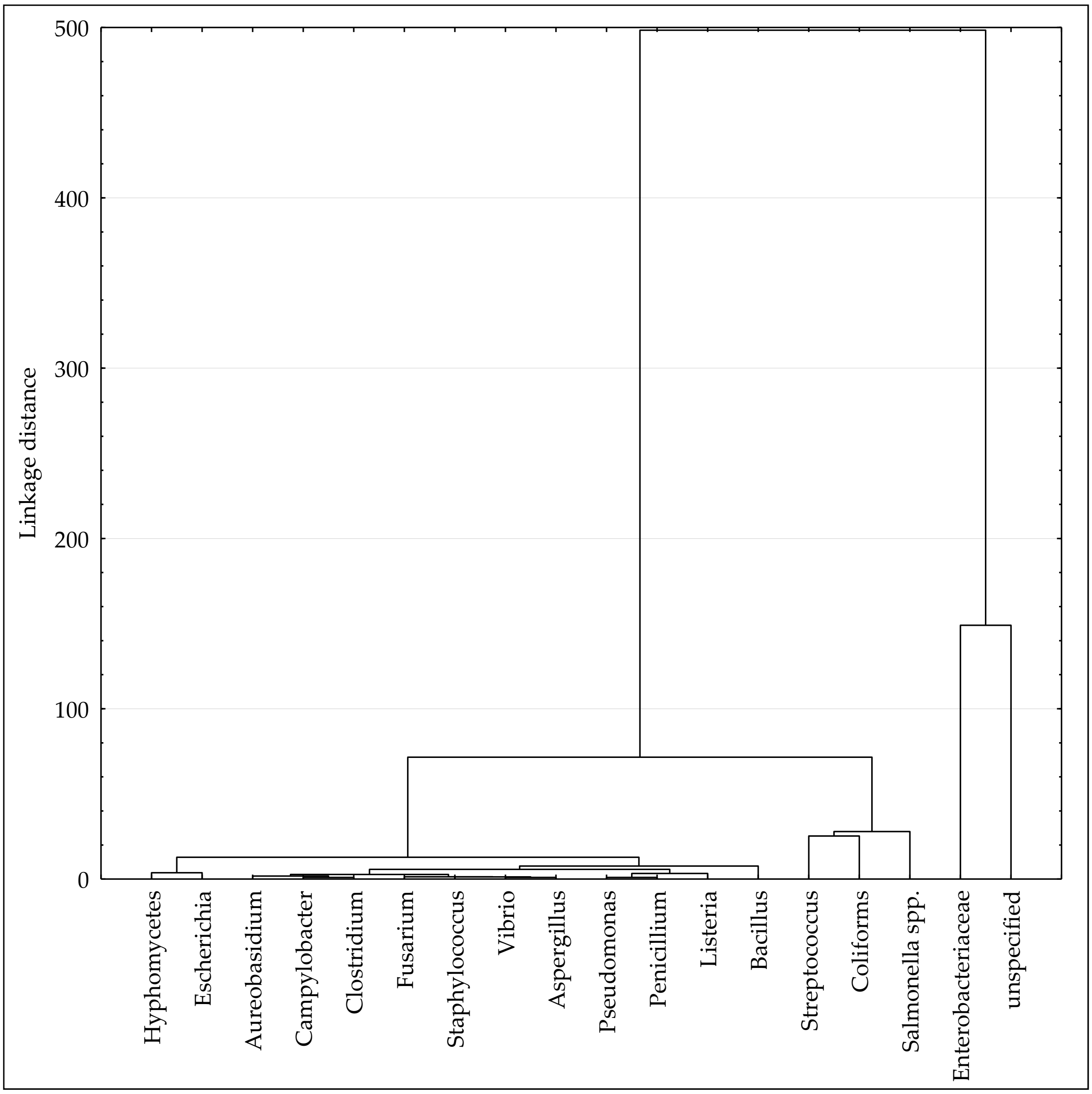

Figure S43. Similarities of RASFF notifications on non-pathogenic microorganisms and distribution status within food using joining.

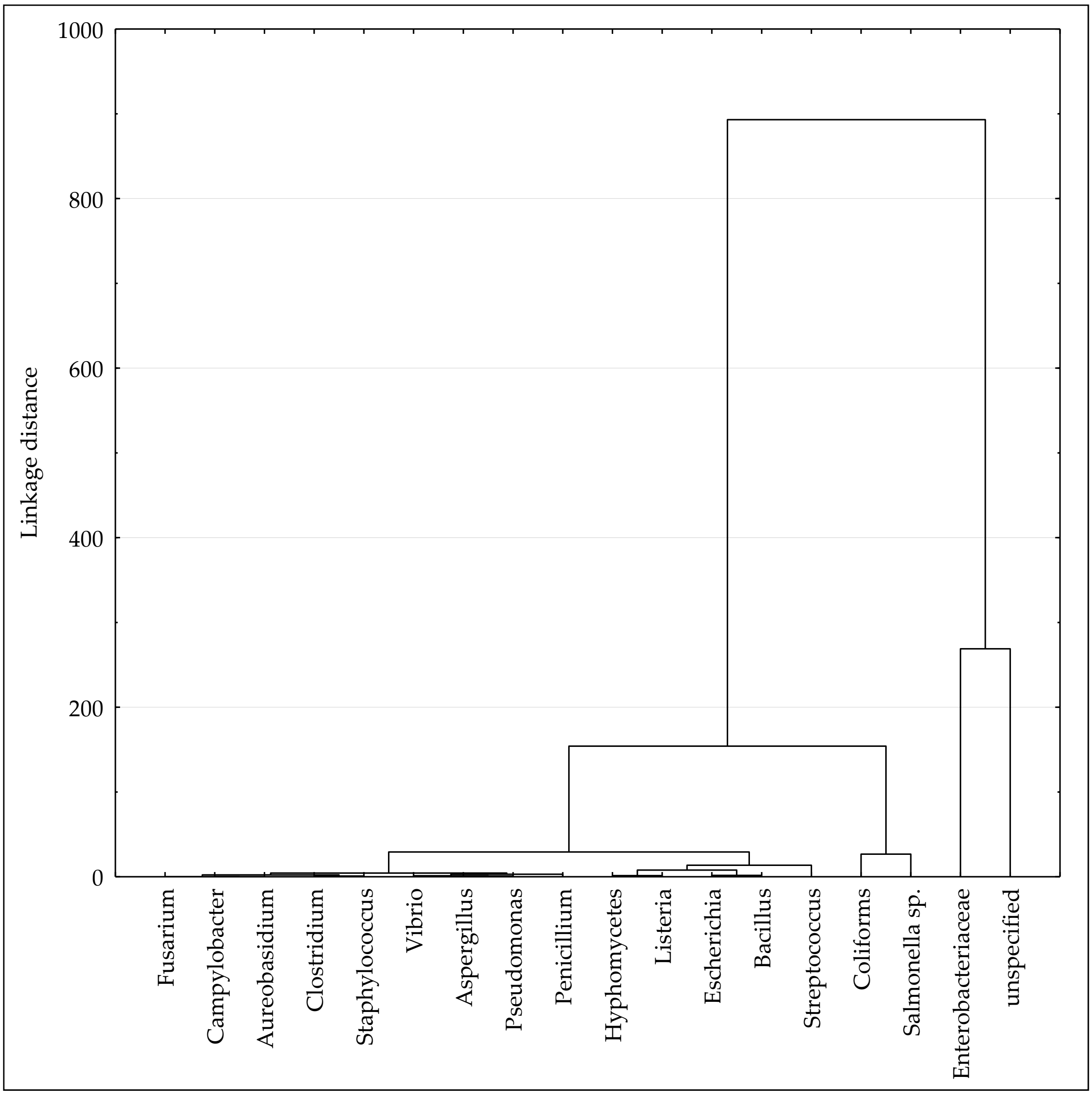

Figure S44. Similarities of RASFF notifications on non-pathogenic microorganisms and risk decision within food using joining.

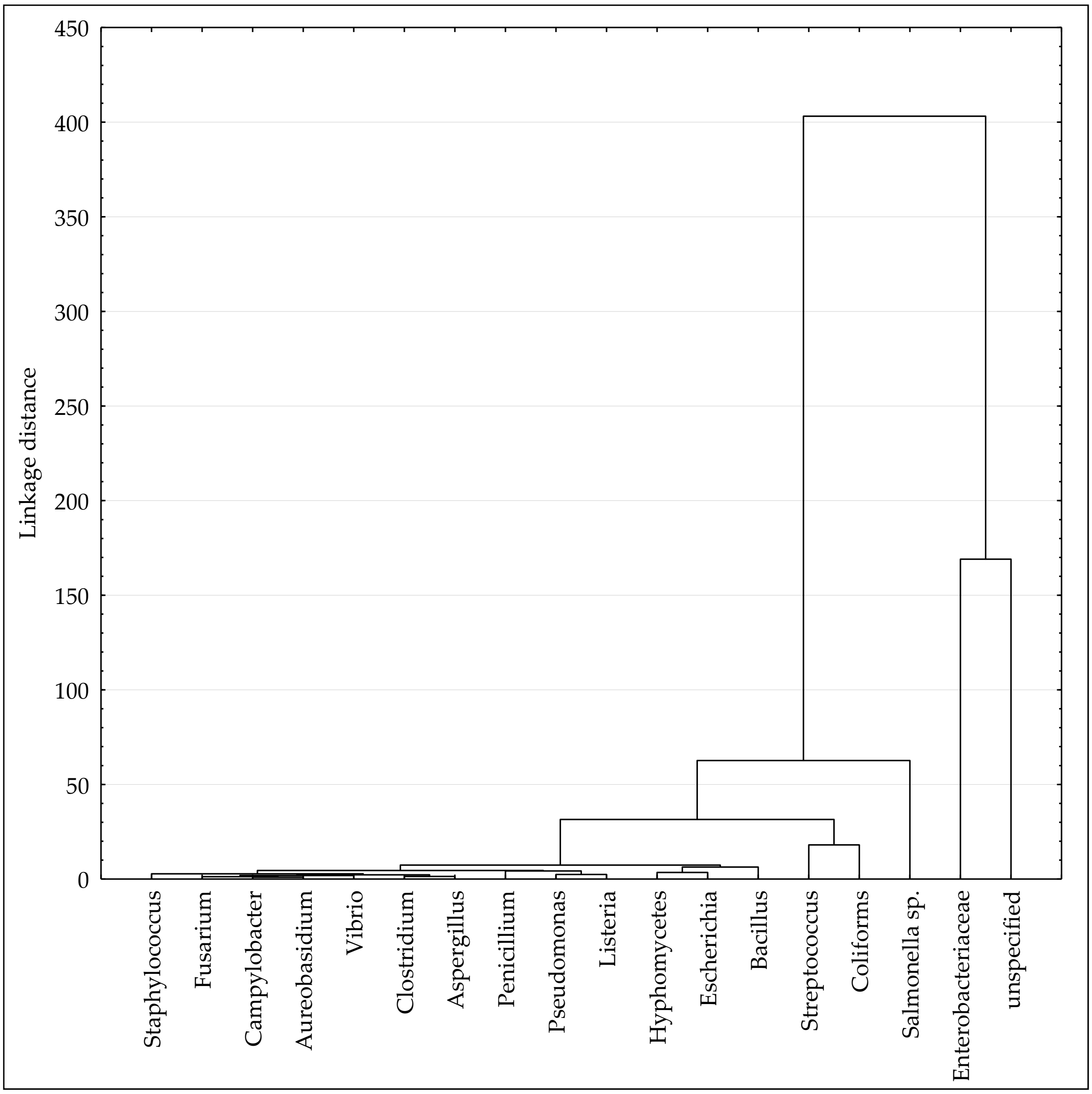

Figure S45. Similarities of RASFF notifications on non-pathogenic microorganisms and action taken within food using joining.

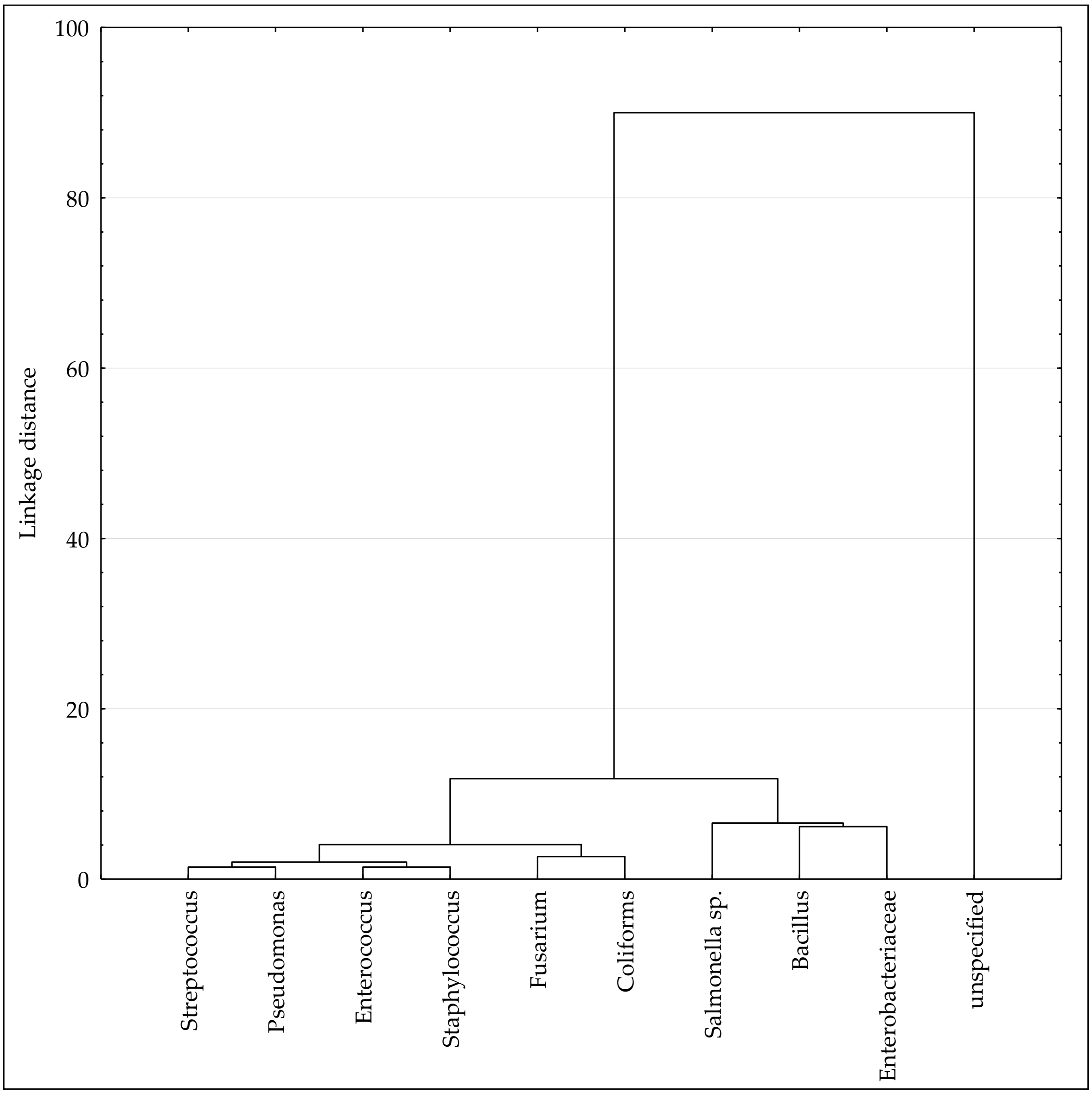

Figure S46. Similarities of RASFF notifications on non-pathogenic microorganisms and year within feed using joining.

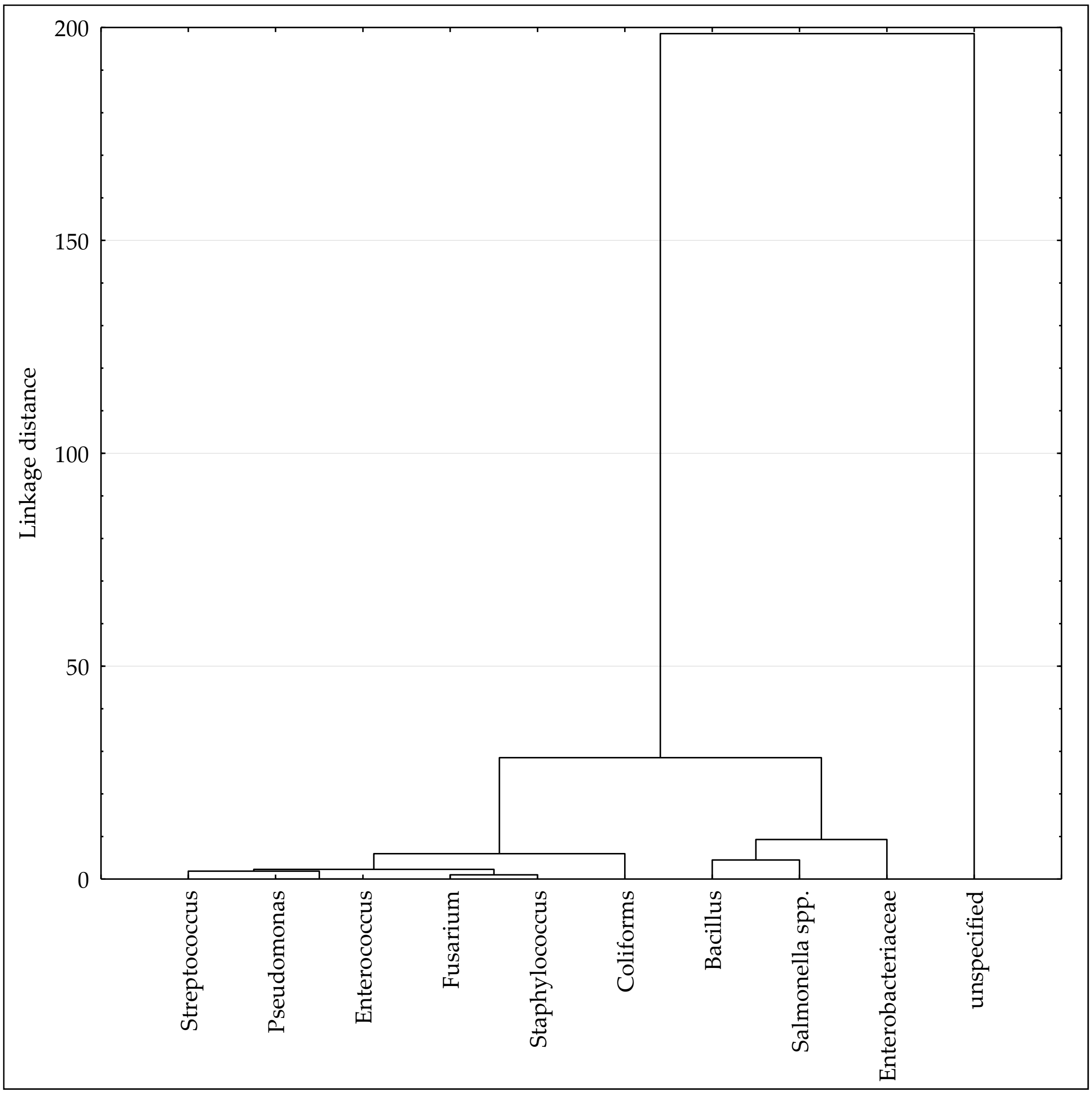

Figure S47. Similarities of RASFF notifications on non-pathogenic microorganisms and product category within feed using joining.

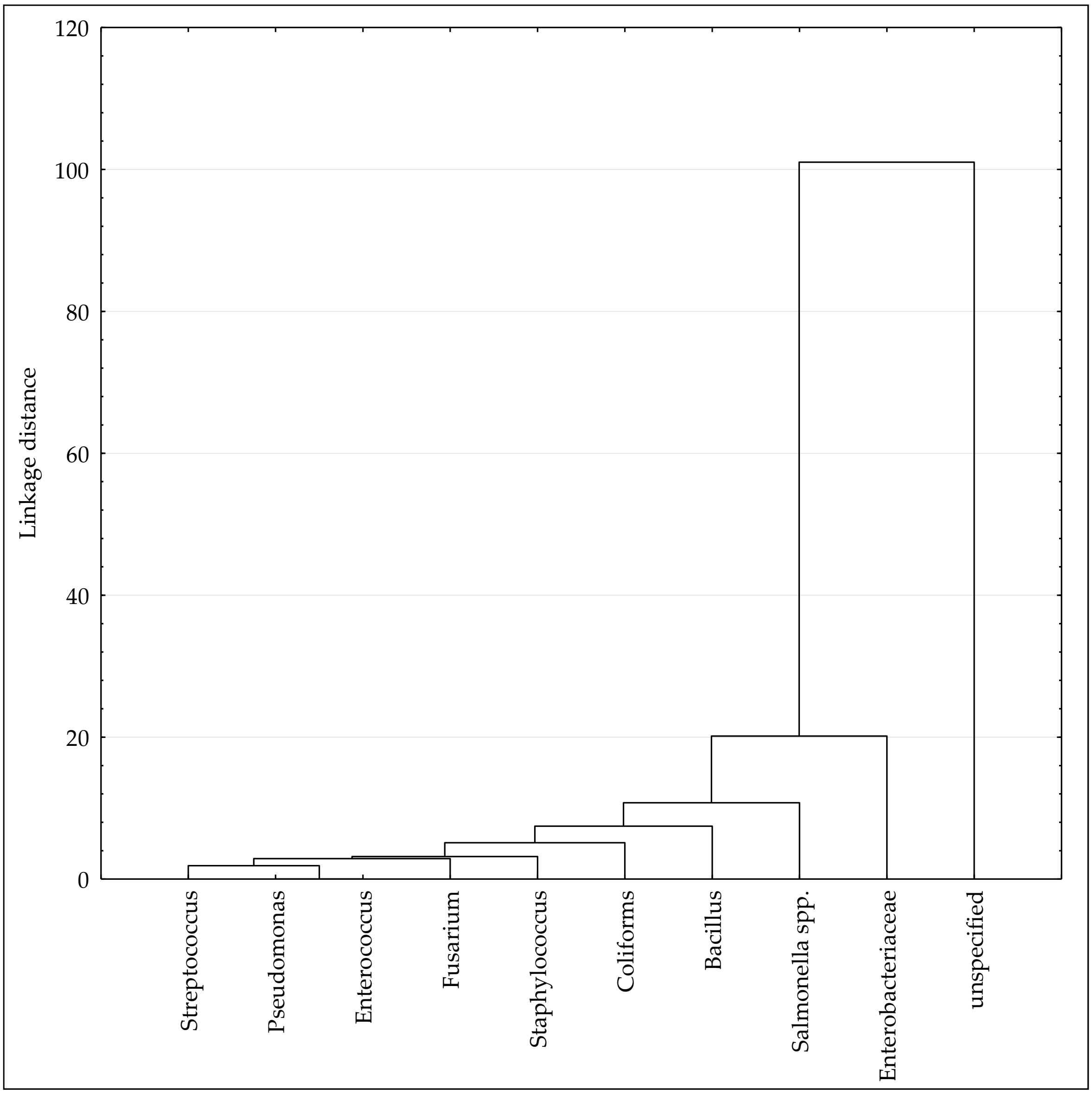

Figure S48. Similarities of RASFF notifications on non-pathogenic microorganisms and notifying country within feed using joining.

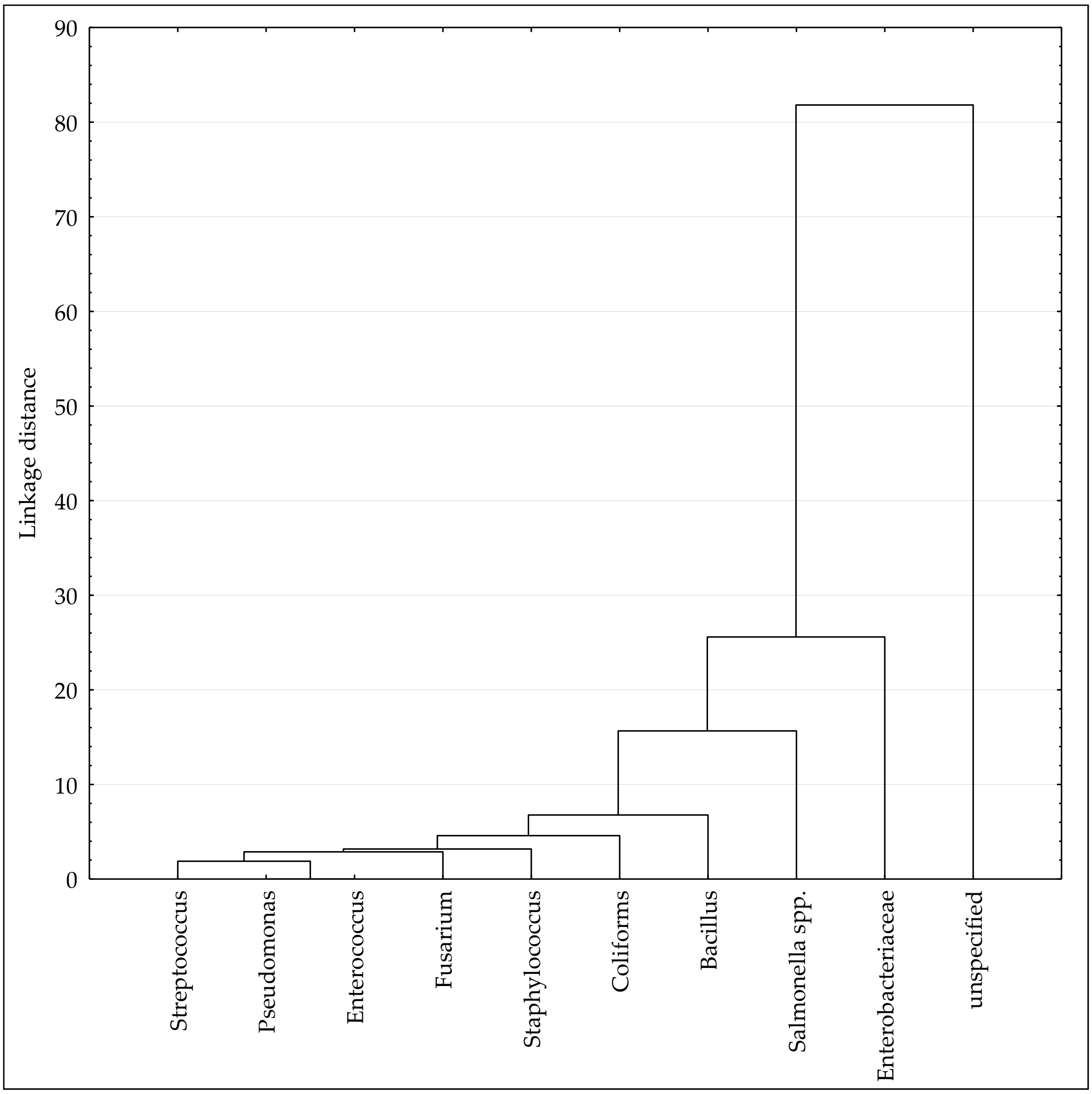

Figure S49. Similarities of RASFF notifications on non-pathogenic microorganisms and origin country within feed using joining.

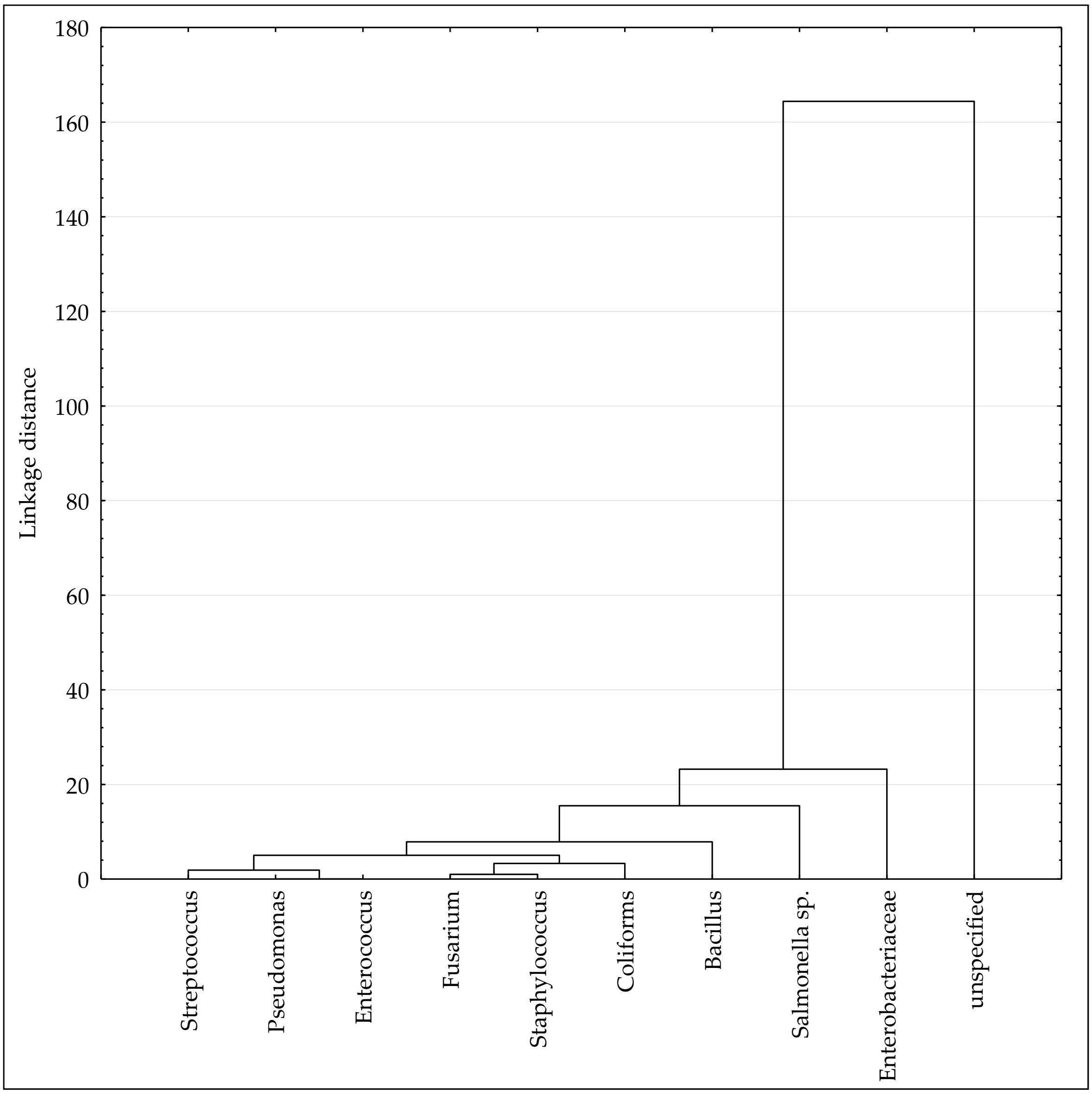

Figure S50. Similarities of RASFF notifications on non-pathogenic microorganisms and notification basis within feed using joining.

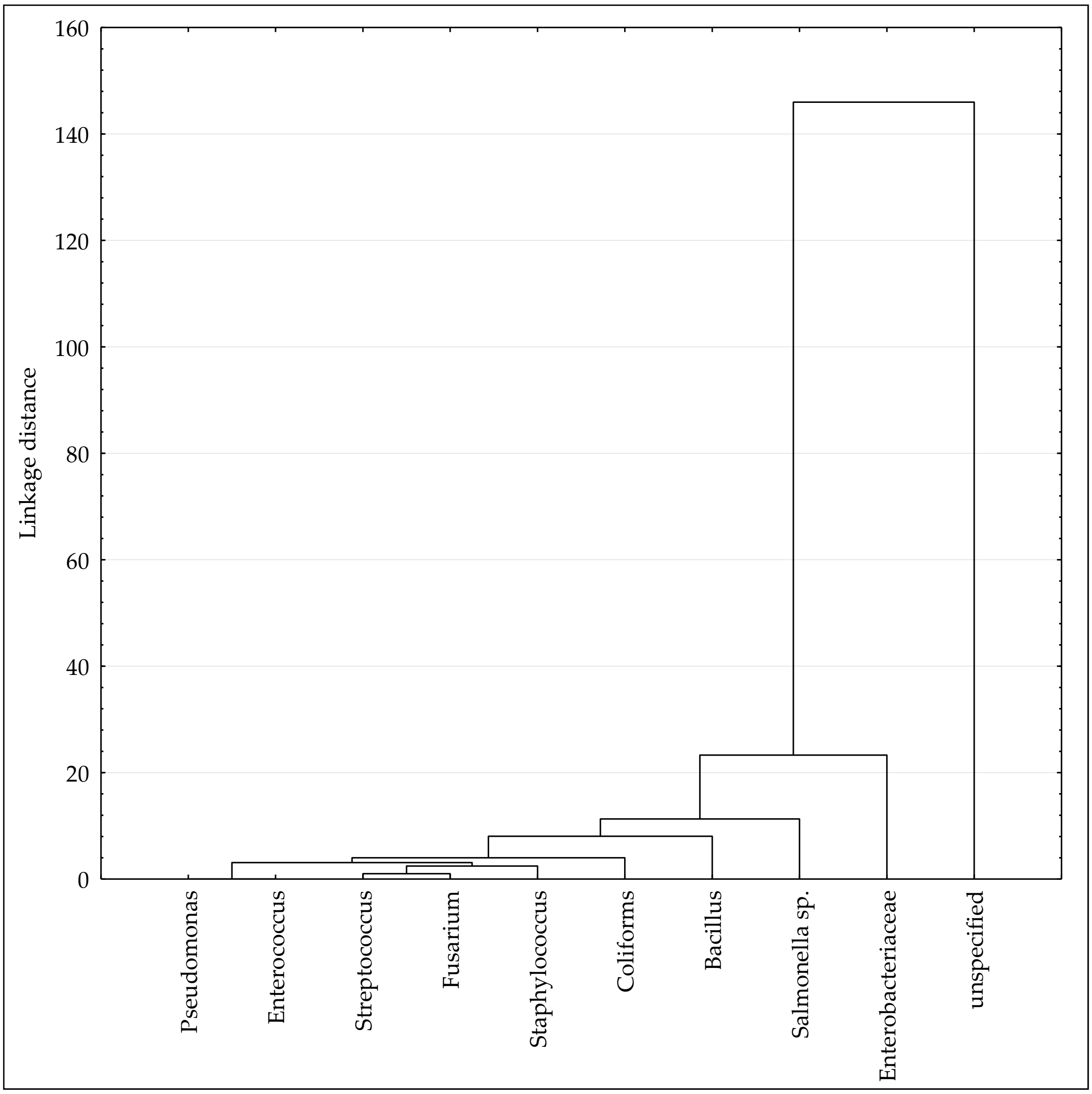

Figure S51. Similarities of RASFF notifications on non-pathogenic microorganisms and notification type within feed using joining.

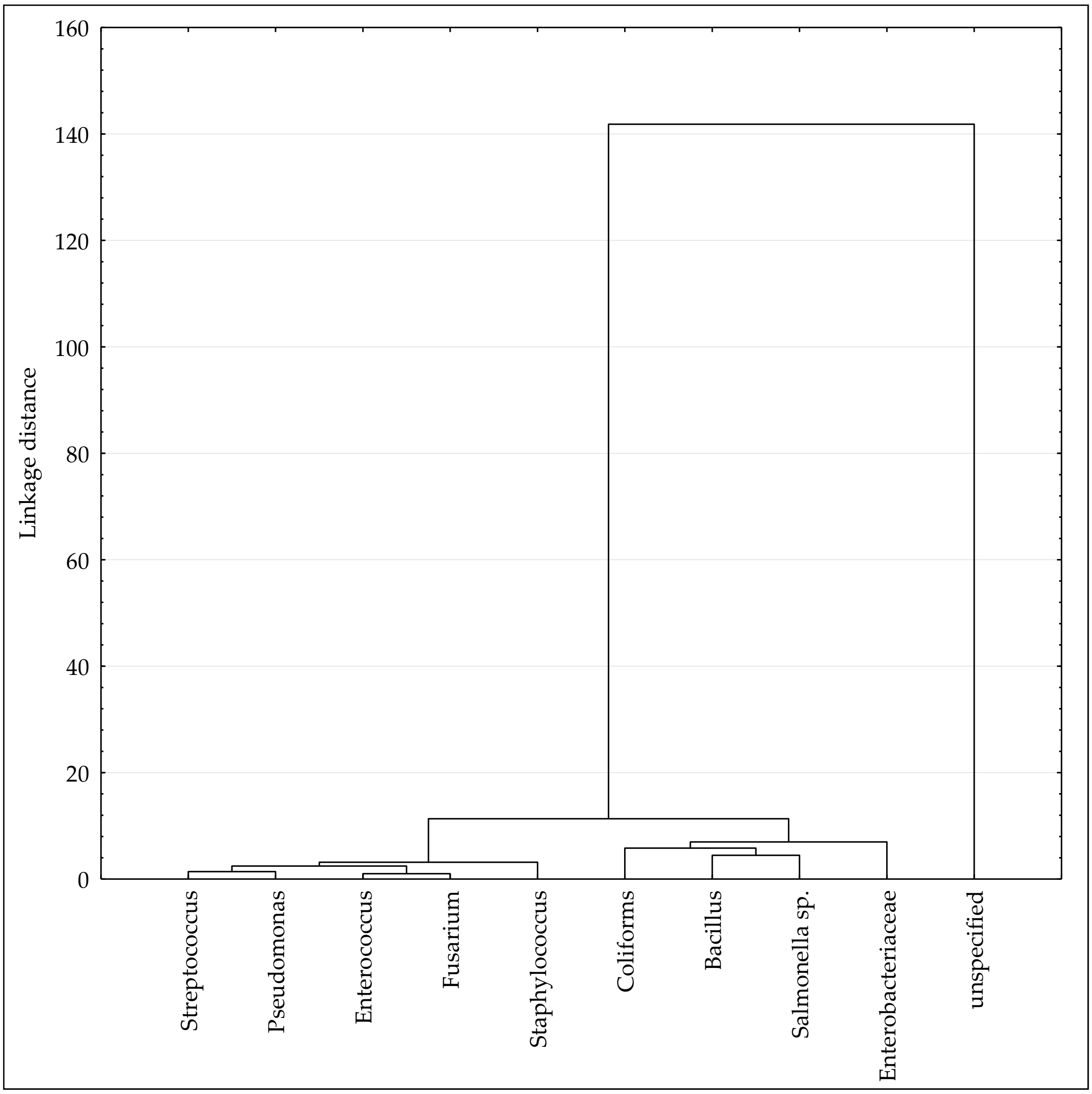

Figure S52. Similarities of RASFF notifications on non-pathogenic microorganisms and distribution status within feed using joining.

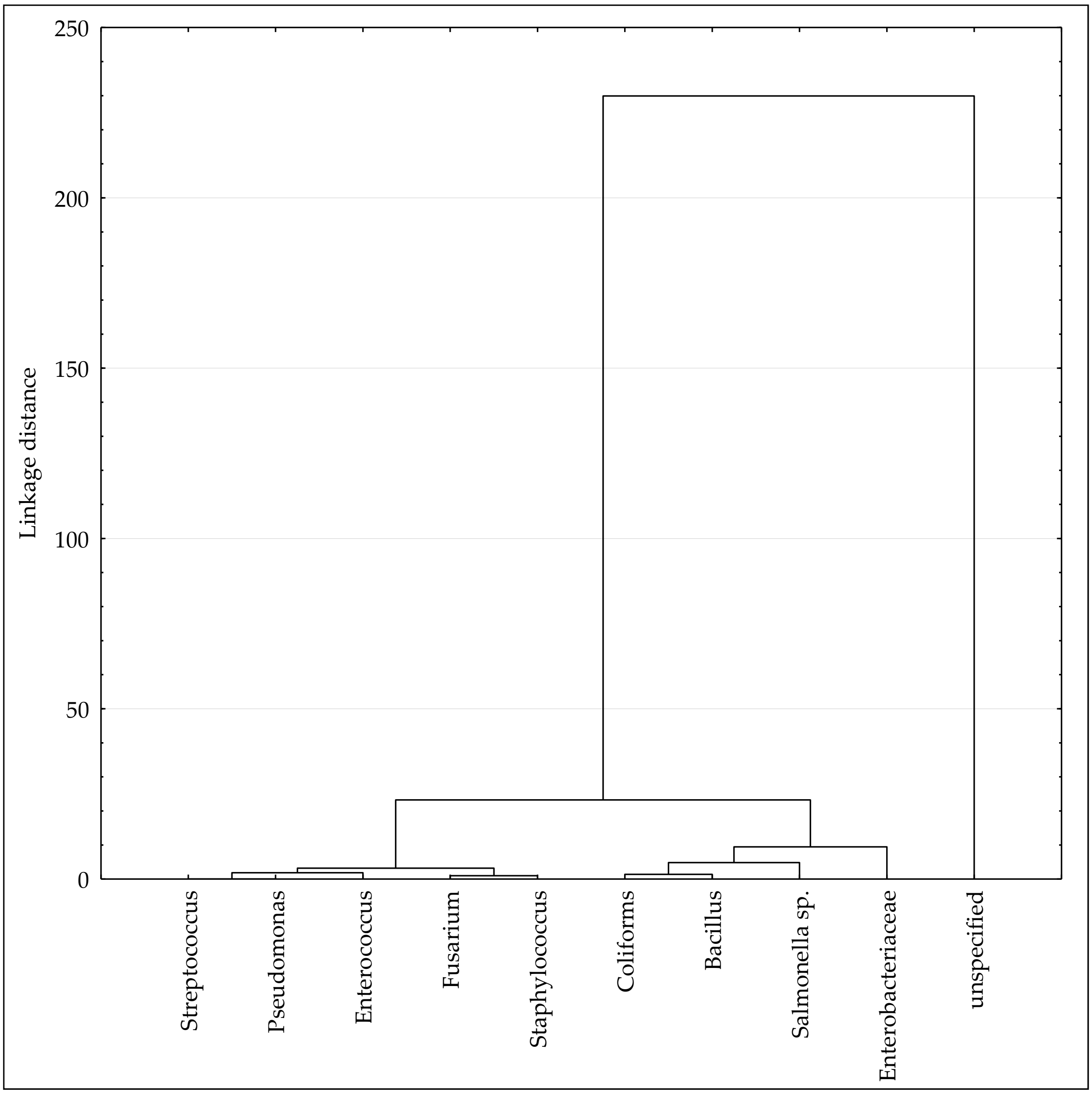

Figure S53. Similarities of RASFF notifications on non-pathogenic microorganisms and risk decision within feed using joining.

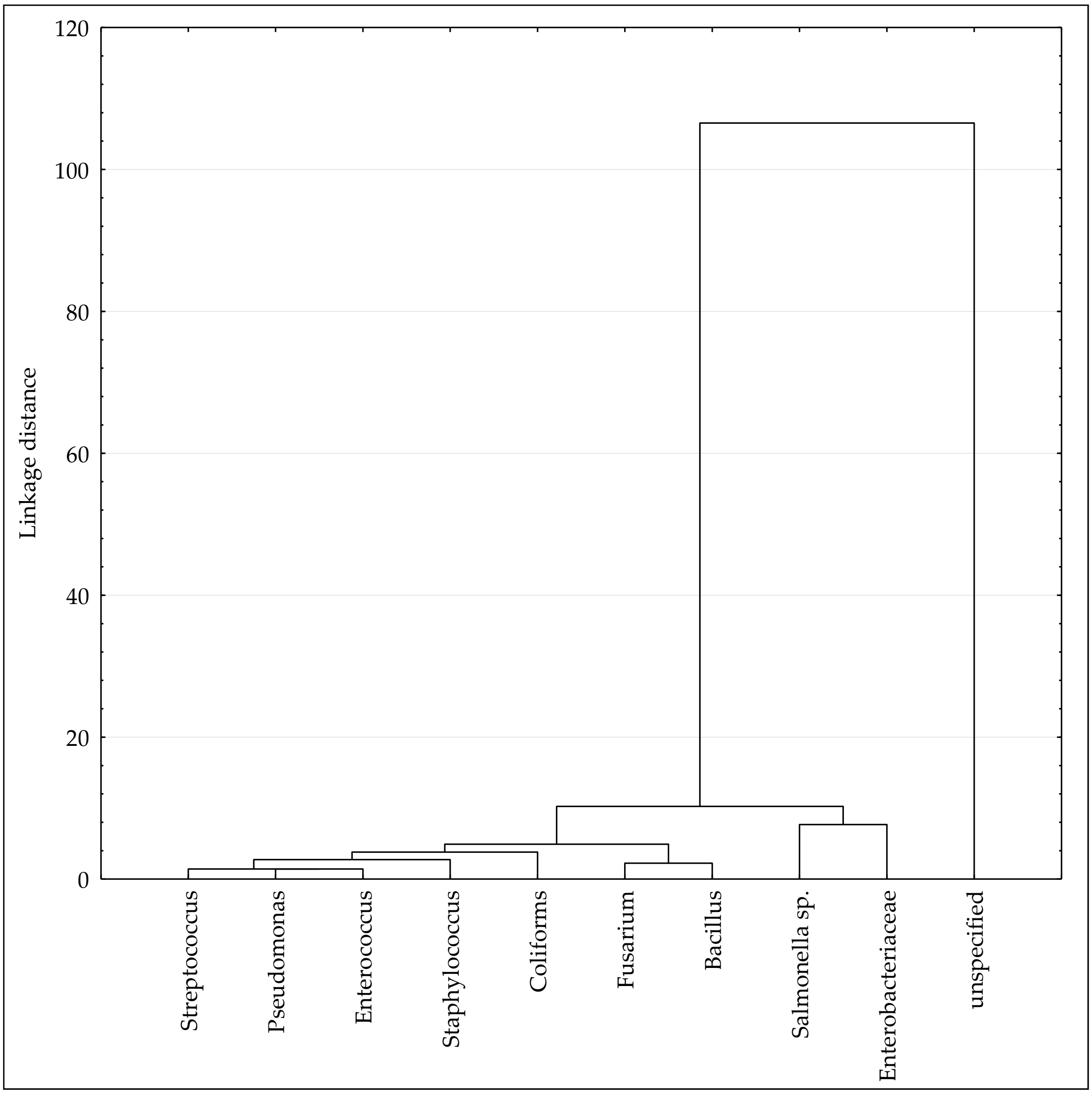

Figure S54. Similarities of RASFF notifications on non-pathogenic microorganisms and action taken within feed using joining.

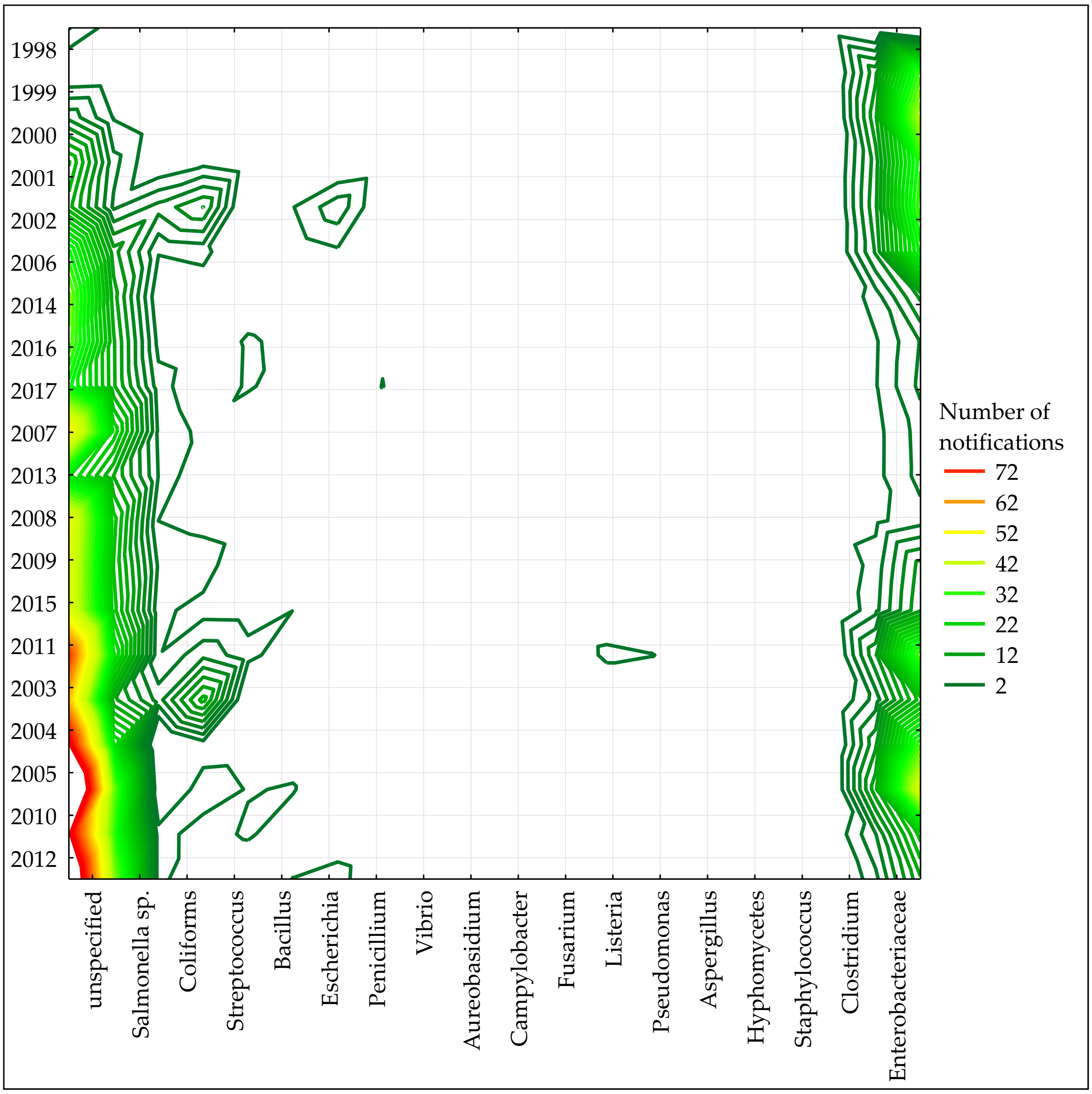

Figure S55. Similarities of RASFF notifications on non-pathogenic microorganisms and year within food using two-way joining.

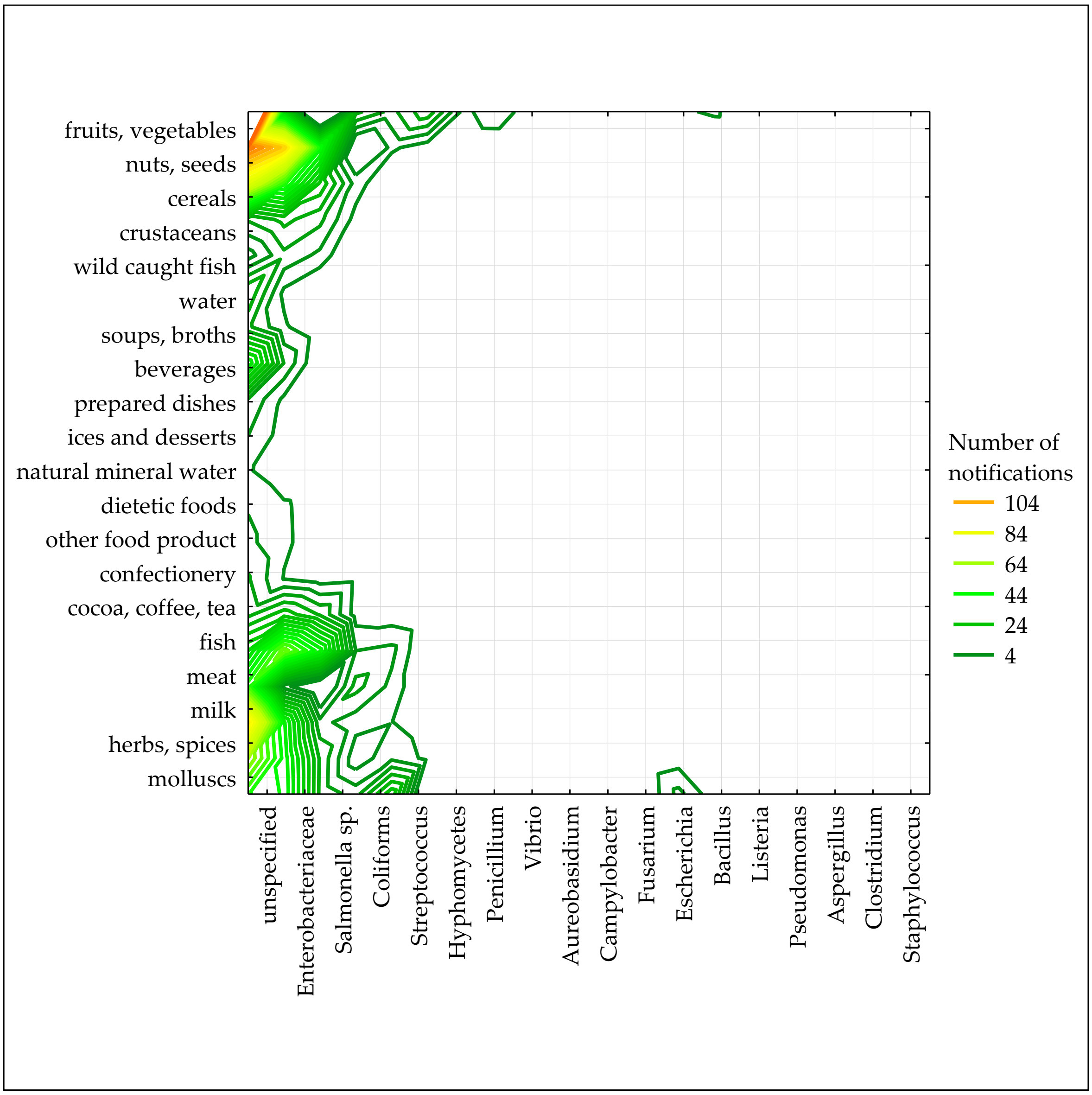

Figure S56. Similarities of RASFF notifications on non-pathogenic microorganisms and product category within food using two-way joining.

beverages – non-alcoholic beverages
cereals – cereals and bakery products
cocoa, coffee, tea – cocoa and cocoa preparations, coffee and tea
crustaceans – crustaceans and products thereof
dietetic foods – dietetic foods, food supplements, fortified foods
fish – fish and fish products
herbs, spices – herbs and spices
meat – meat and meat products (other than poultry)
milk – milk and milk products
molluscs – molluscs and products thereof - (obsolete)
nuts, seeds – nuts, nut products and seeds
other food product – other food product / mixed
prepared dishes – prepared dishes and snacks
soups, broths – soups, broths, sauces and condiments
water – water for human consumption (other)
wild caught fish – wild caught fish and products thereof (other than crustaceans and molluscs) - (obsolete)

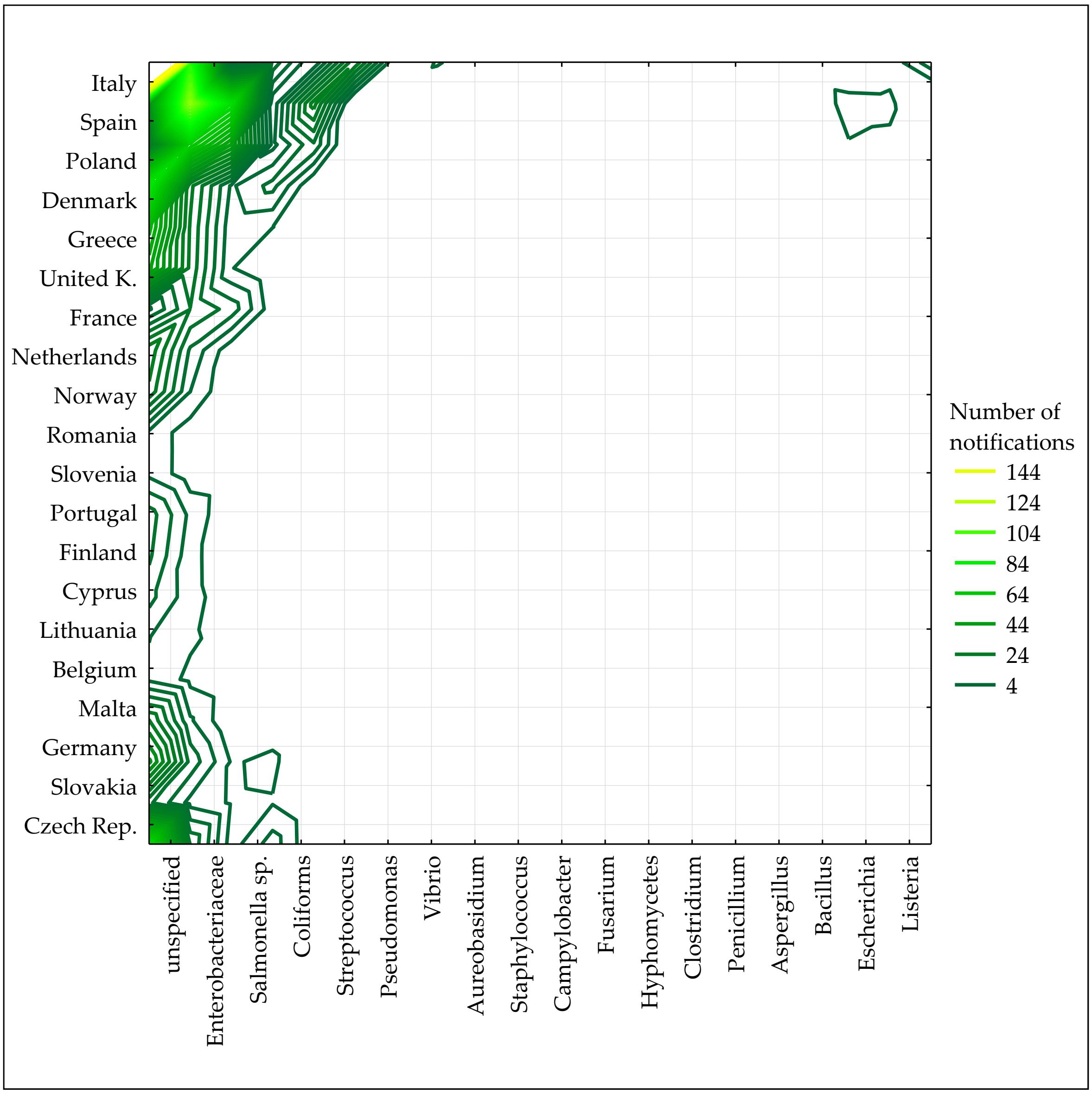

Figure S57. Similarities of RASFF notifications on non-pathogenic microorganisms and notifying country within food using two-way joining.

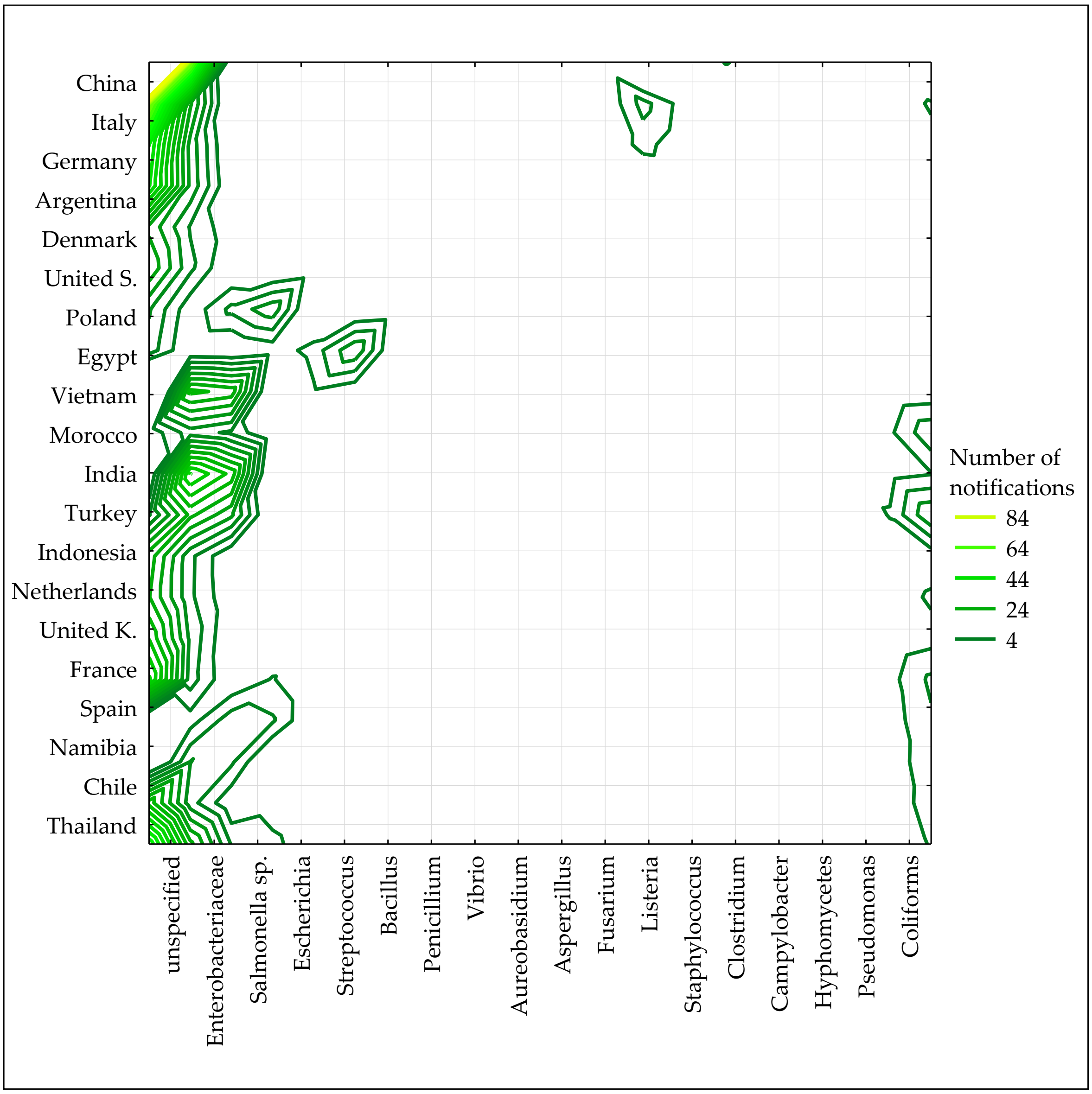

Figure S58. Similarities of RASFF notifications on non-pathogenic microorganisms and origin country within food using two-way joining.

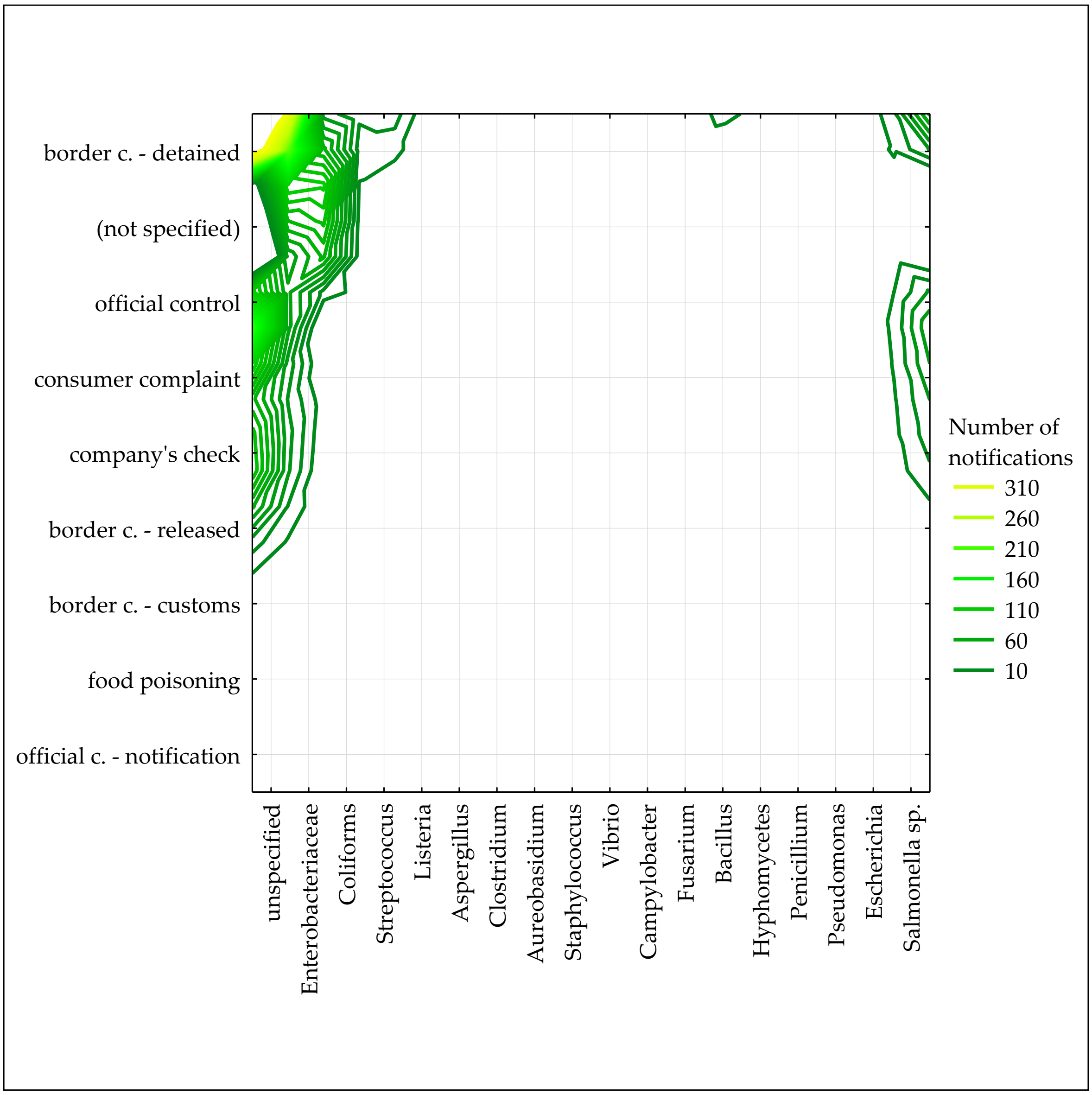

Figure S59. Similarities of RASFF notifications on non-pathogenic microorganisms and notification basis within food using two-way joining.

border c. - customs – border control - consignment under customs
border c. - detained – border control - consignment detained
border c. - released – border control - consignment released
company's check – company's own check
official c. - notification – official control following RASFF notification
official control – official control on the market

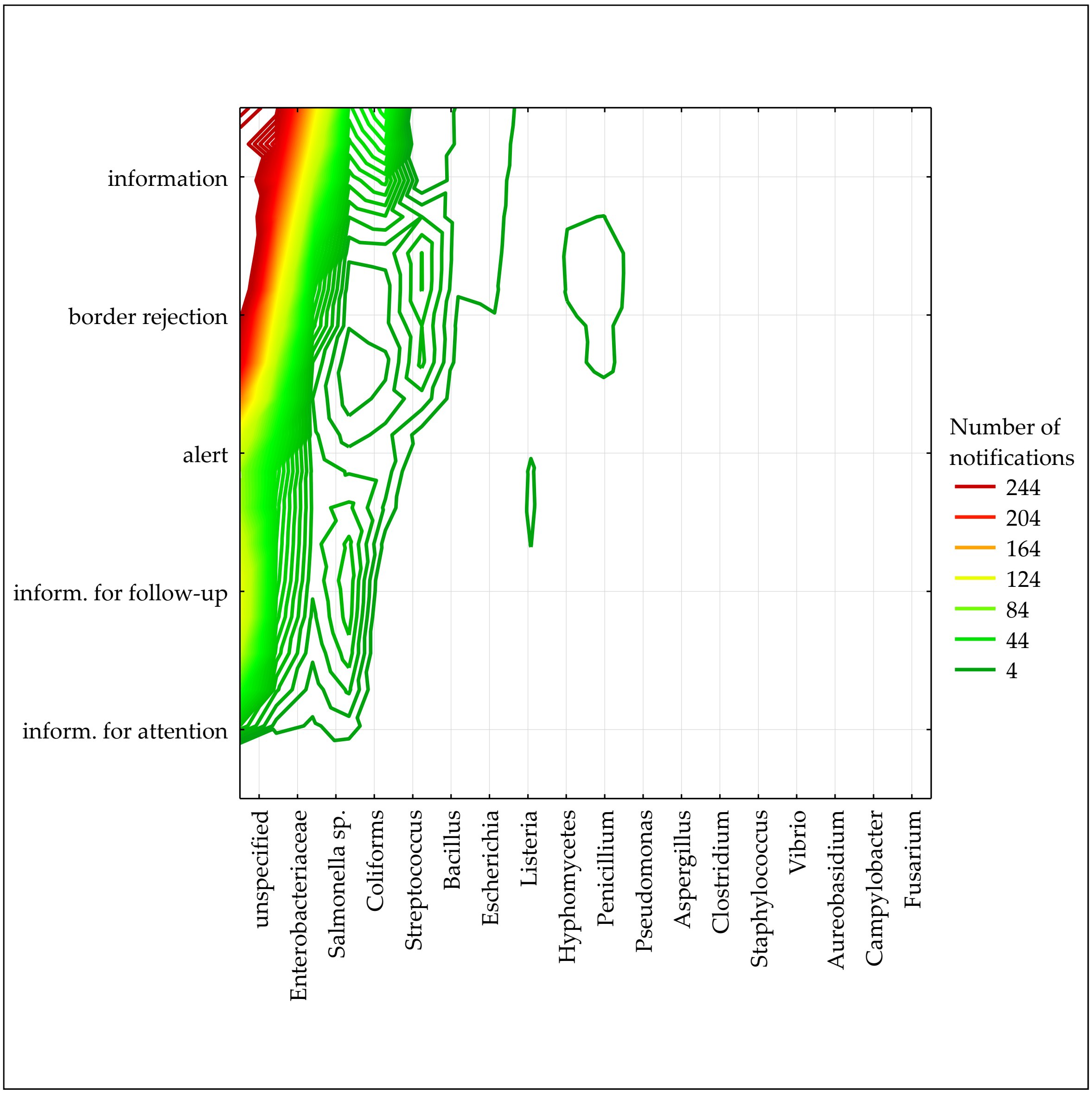

Figure S60. Similarities of RASFF notifications on non-pathogenic microorganisms and notification type within food using two-way joining.

inform. for attention – information for attention
inform. for follow-up – information for follow-up

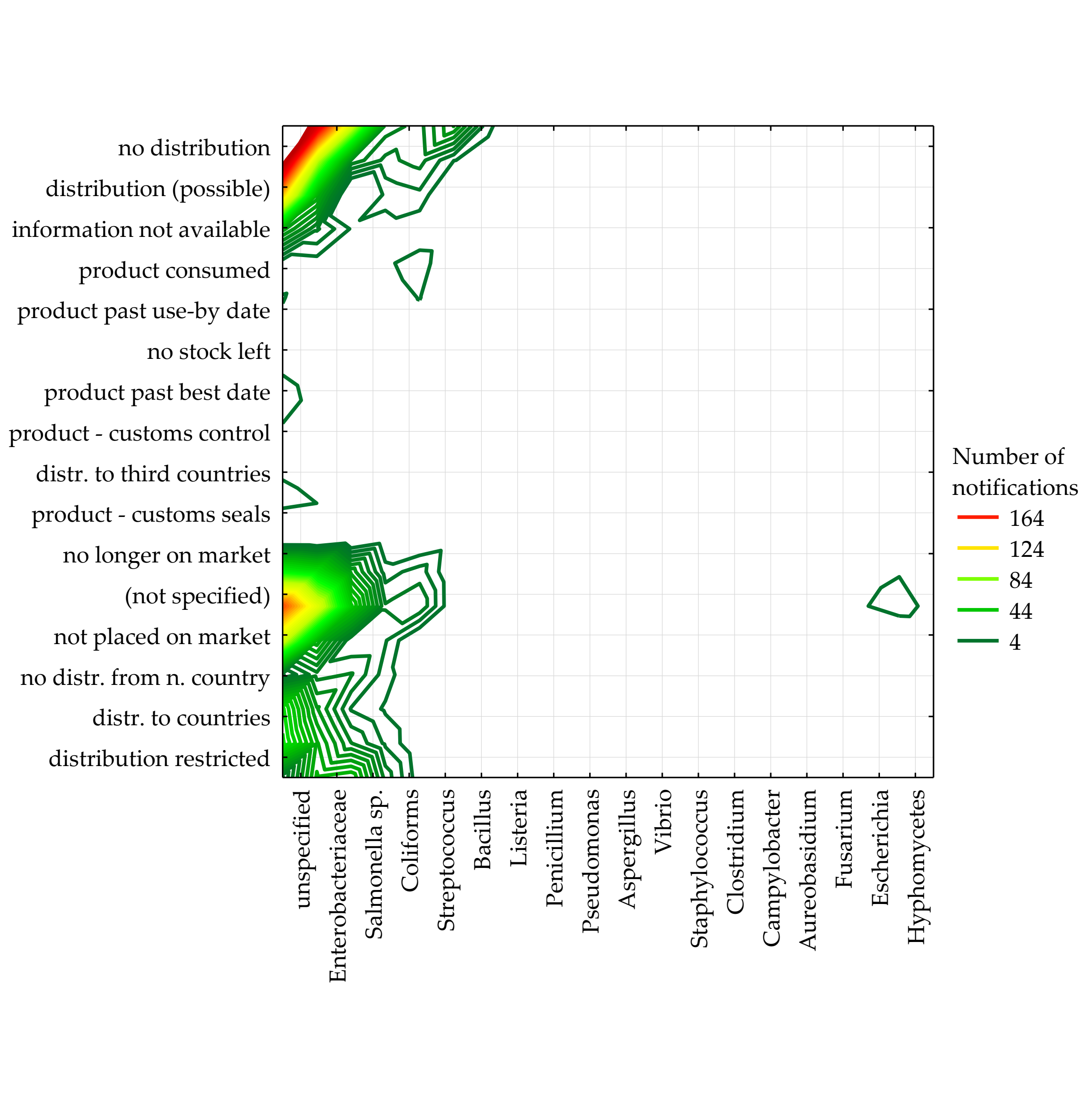

Figure S61. Similarities of RASFF notifications on non-pathogenic microorganisms and distribution status within food using two-way joining.

distr. to countries – distribution to other member countries
distr. to third countries – distribution to third countries
distribution (possible) – distribution on the market (possible)
distribution restricted – distribution restricted to notifying country
information not available – information on distribution not (yet) available
no distr. from n. country – no distribution from notifying country
no longer on market – product (presumably) no longer on the market
not placed on market – product not (yet) placed on the market
product - customs control – product under customs control
product - customs seals – product allowed to travel to destination under customs seals
product consumed – product already consumed
product past best date – product past best before date

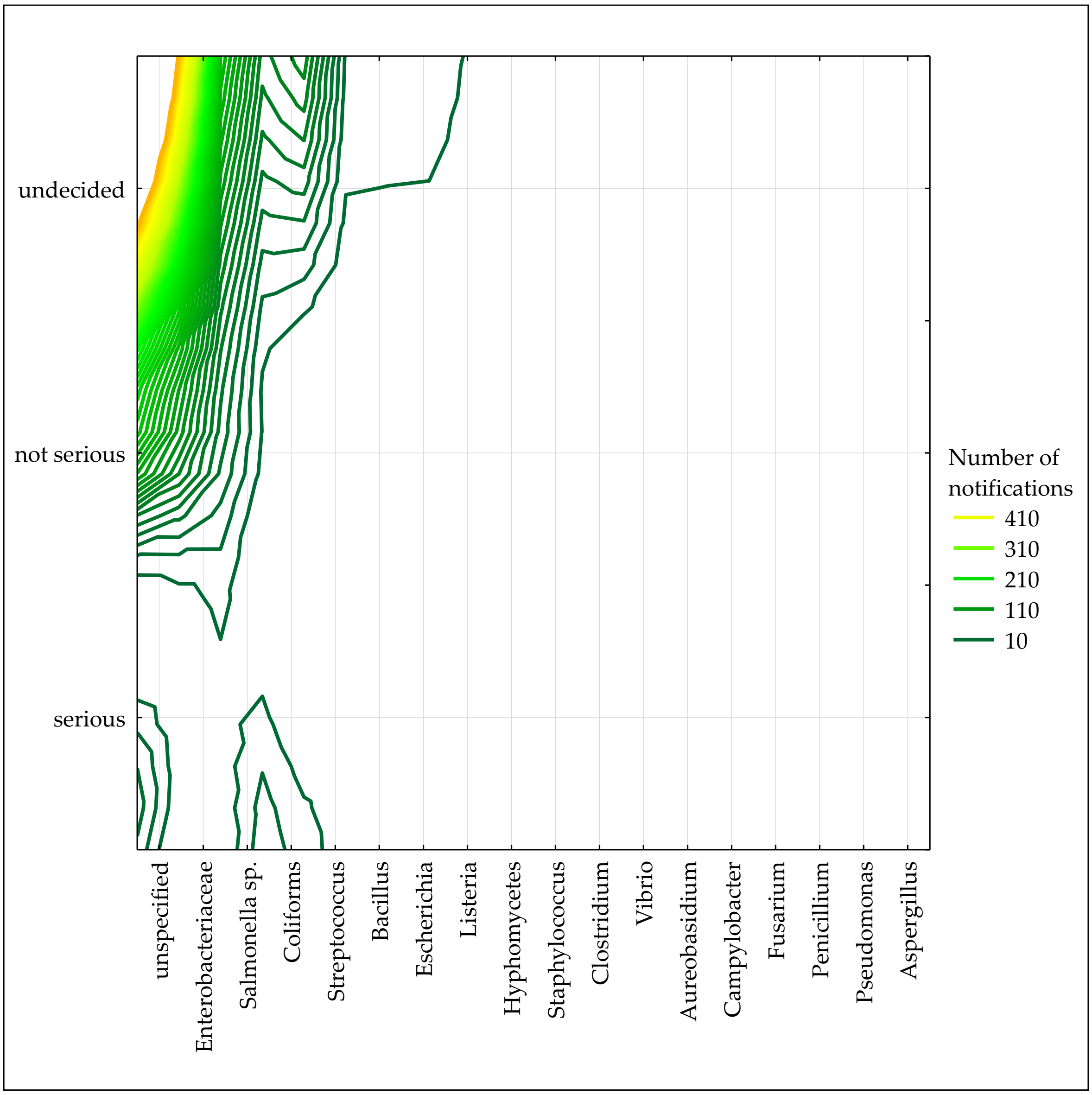

Figure S62. Similarities of RASFF notifications on non-pathogenic microorganisms and risk decision within food using two-way joining.

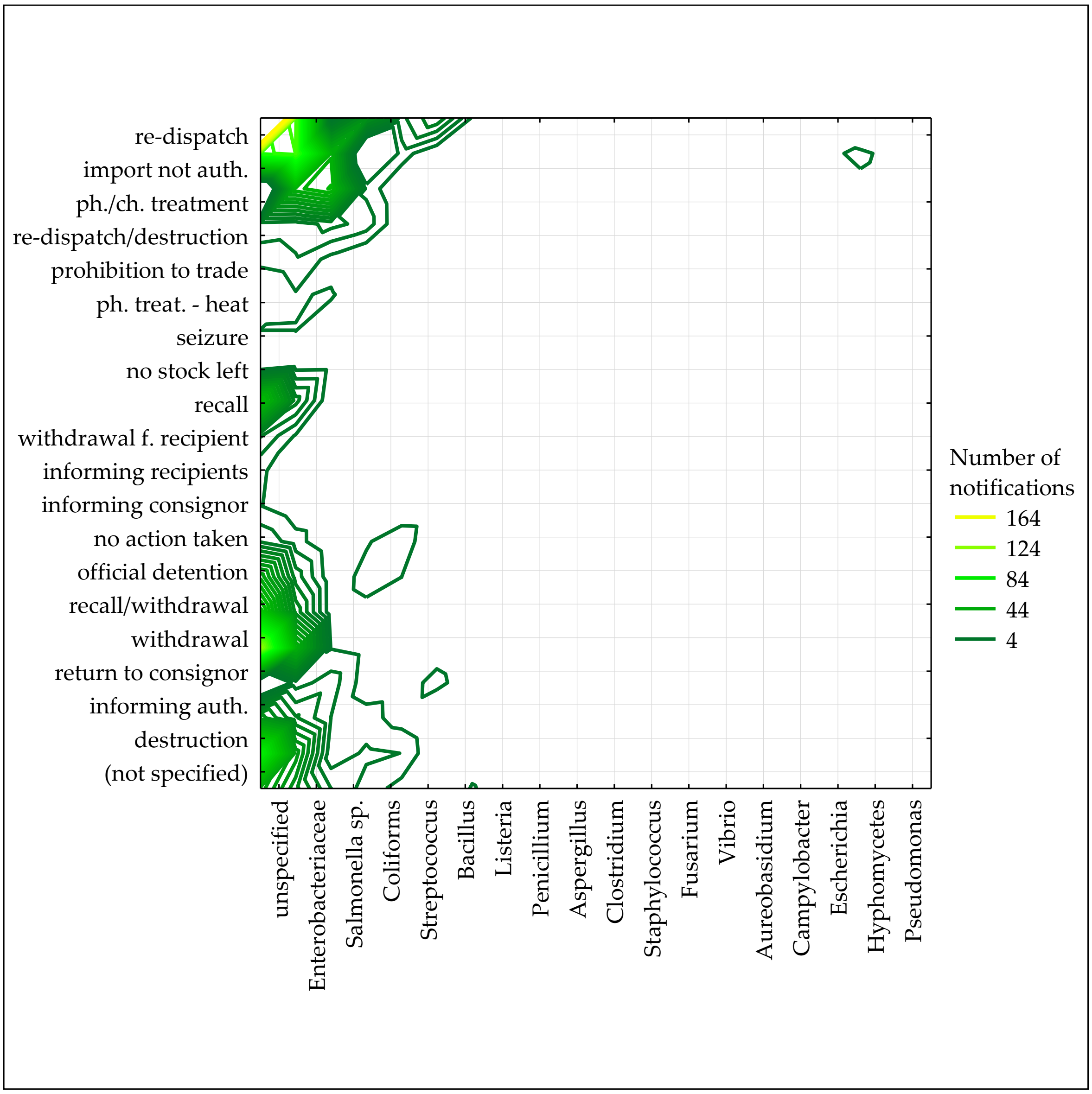

Figure S63. Similarities of RASFF notifications on non-pathogenic microorganisms and action taken within food using two-way joining.

import not auth. – import not authorised
informing auth. – informing authorities
ph. treat. - heat – physical treatment - heat treatment
ph./ch. treatment – physical/chemical treatment
prohibition to trade – prohibition to trade - sales ban
recall – recall from consumers
recall/withdrawal – product recall or withdrawal
re-dispatch/destruction – re-dispatch or destruction
withdrawal f. recipient – withdrawal from recipient(s)
withdrawal – withdrawal from the market

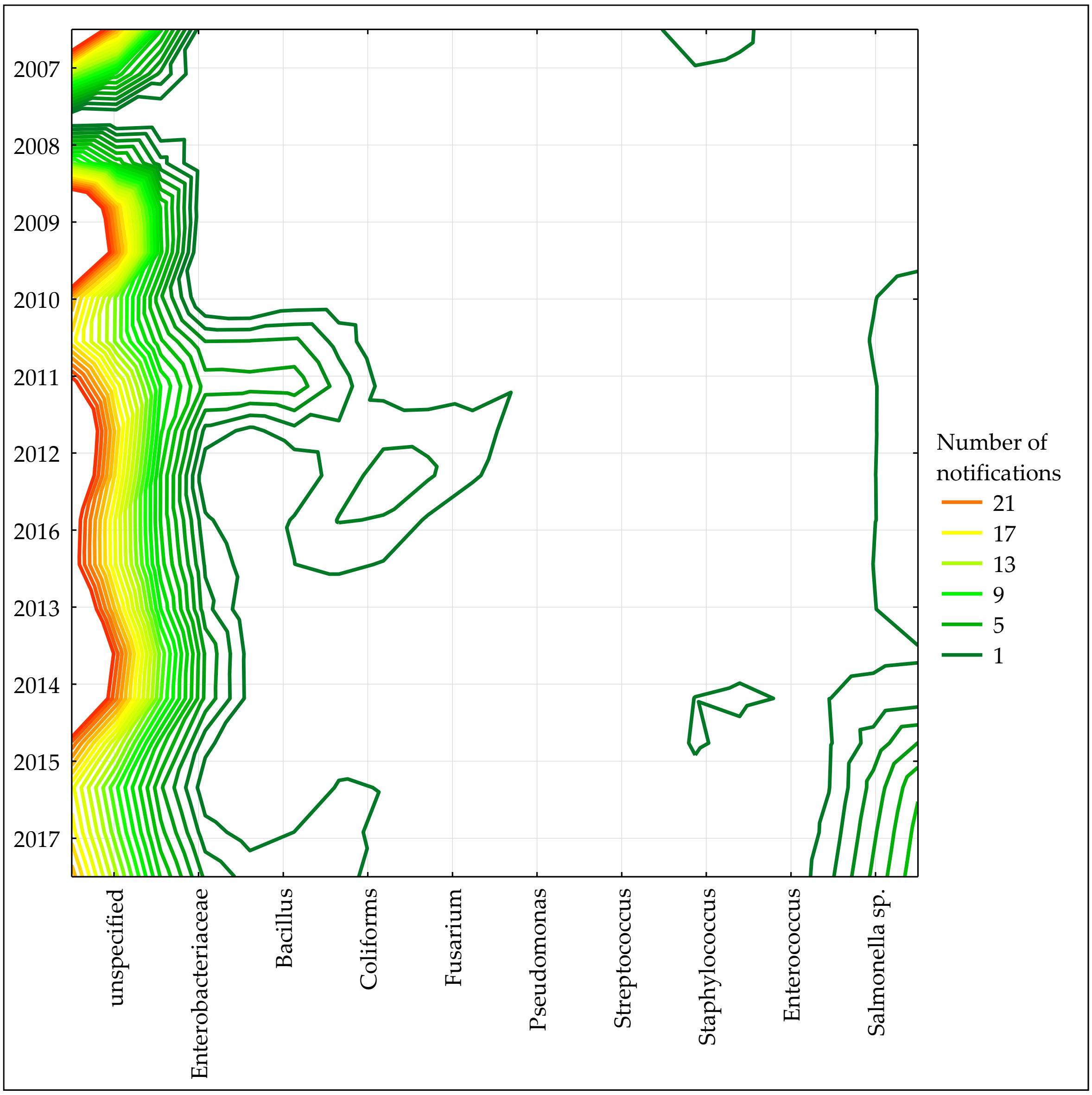

Figure S64. Similarities of RASFF notifications on non-pathogenic microorganisms and year within feed using two-way joining.

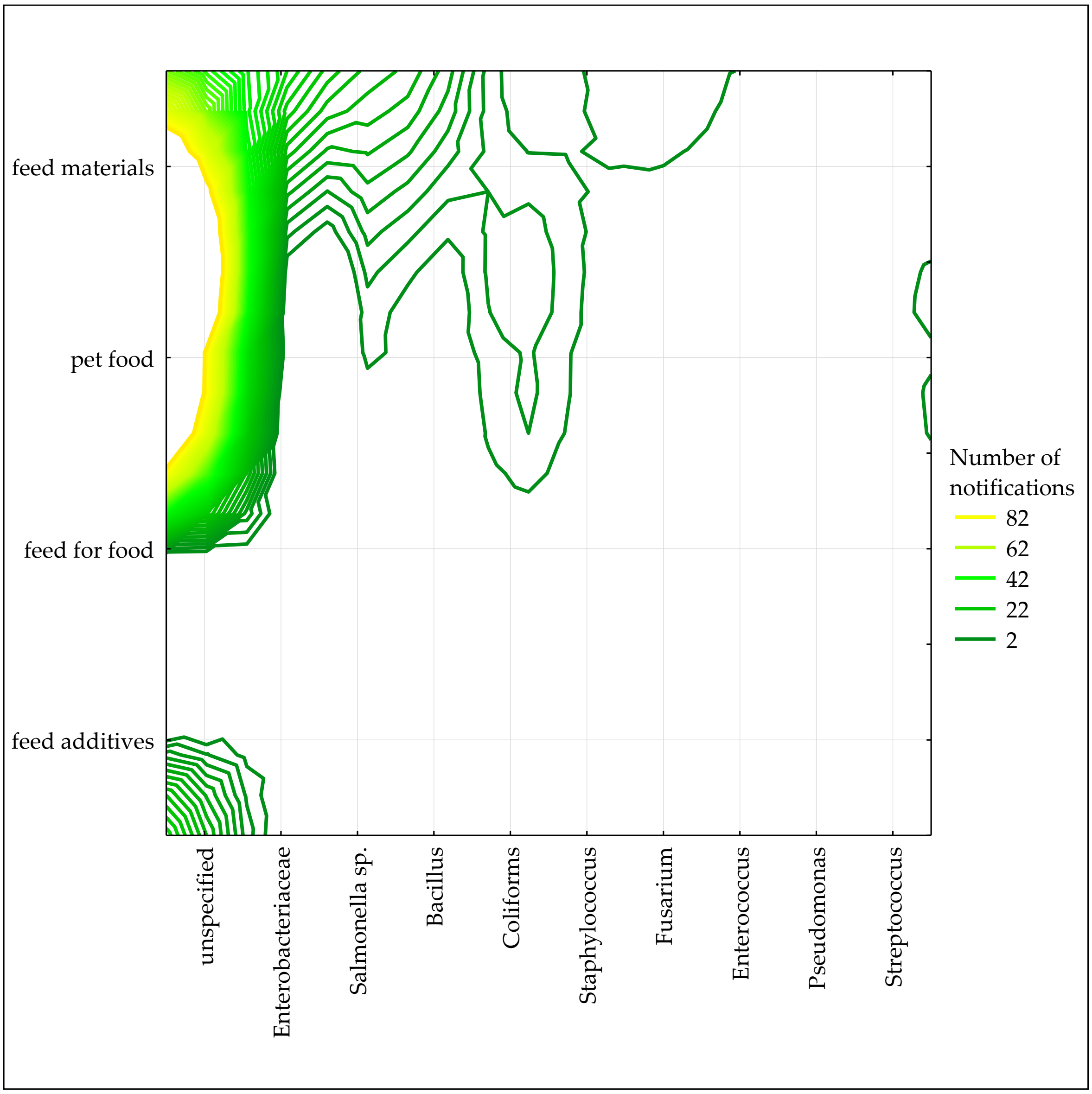

Figure S65. Similarities of RASFF notifications on non-pathogenic microorganisms and product category within feed using two-way joining.

feed for food – feed for food-producing animals - (obsolete)

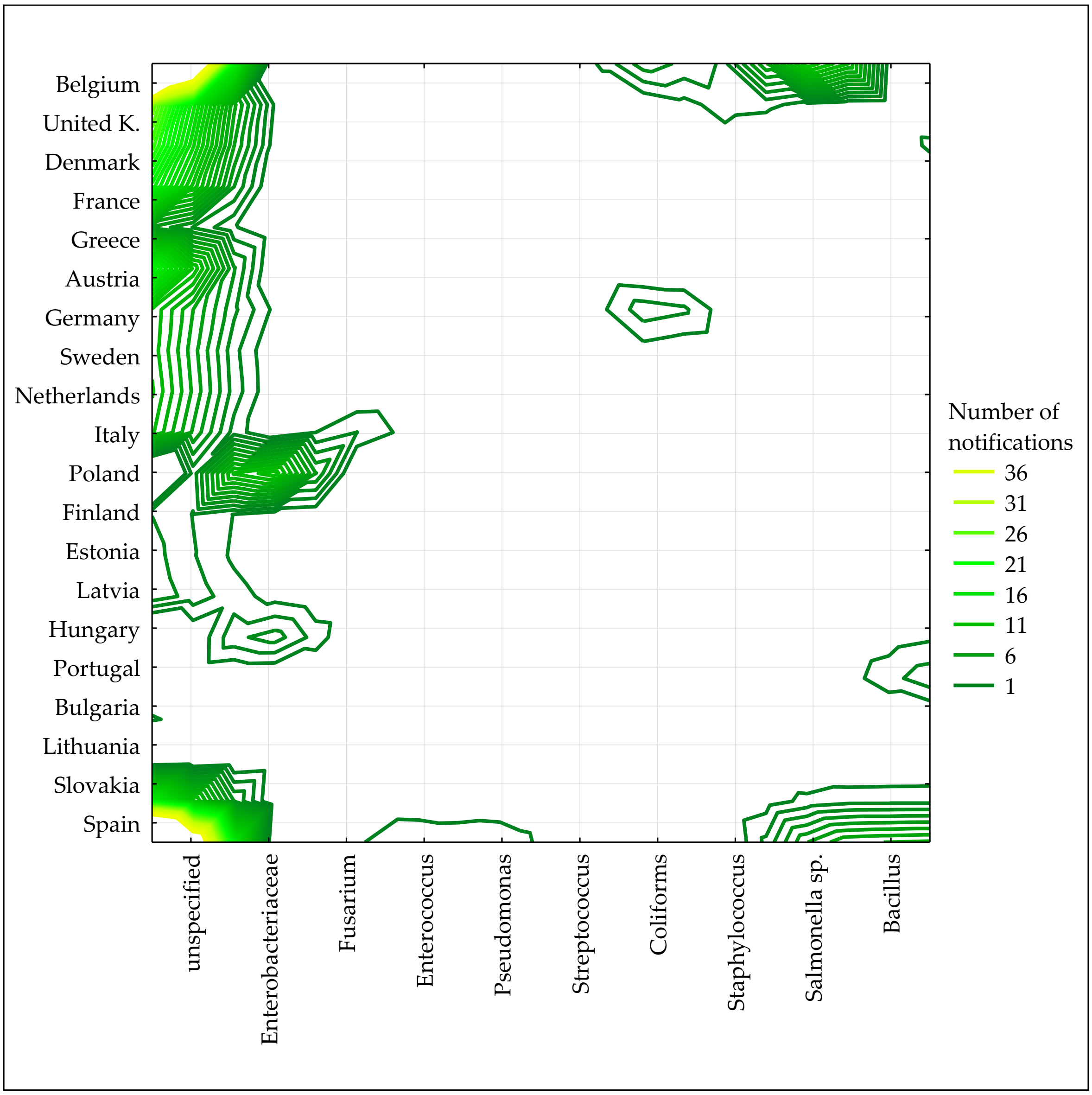

Figure S66. Similarities of RASFF notifications on non-pathogenic microorganisms and notifying country within feed using two-way joining.

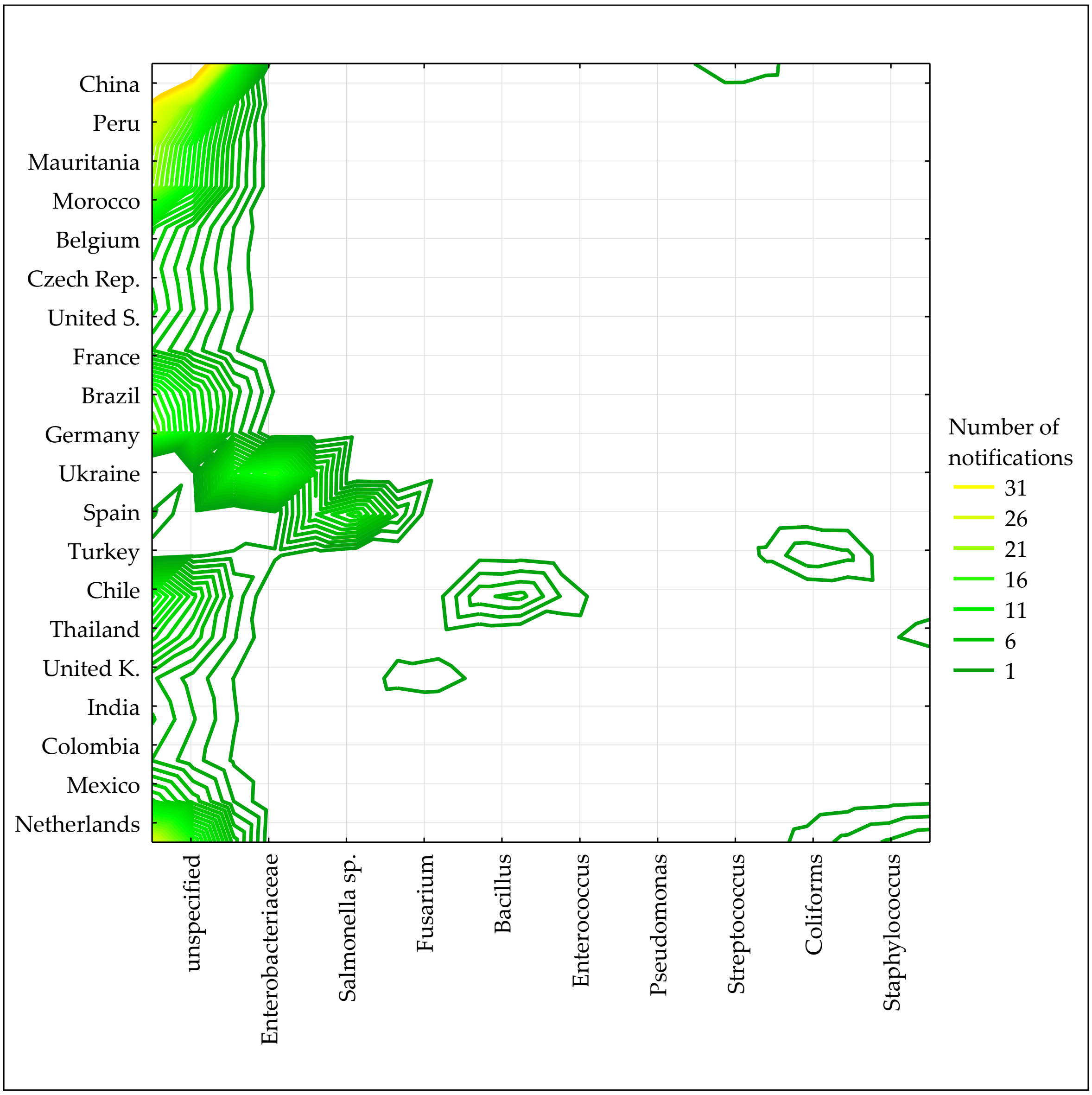

Figure S67. Similarities of RASFF notifications on non-pathogenic microorganisms and origin country within feed using two-way joining.

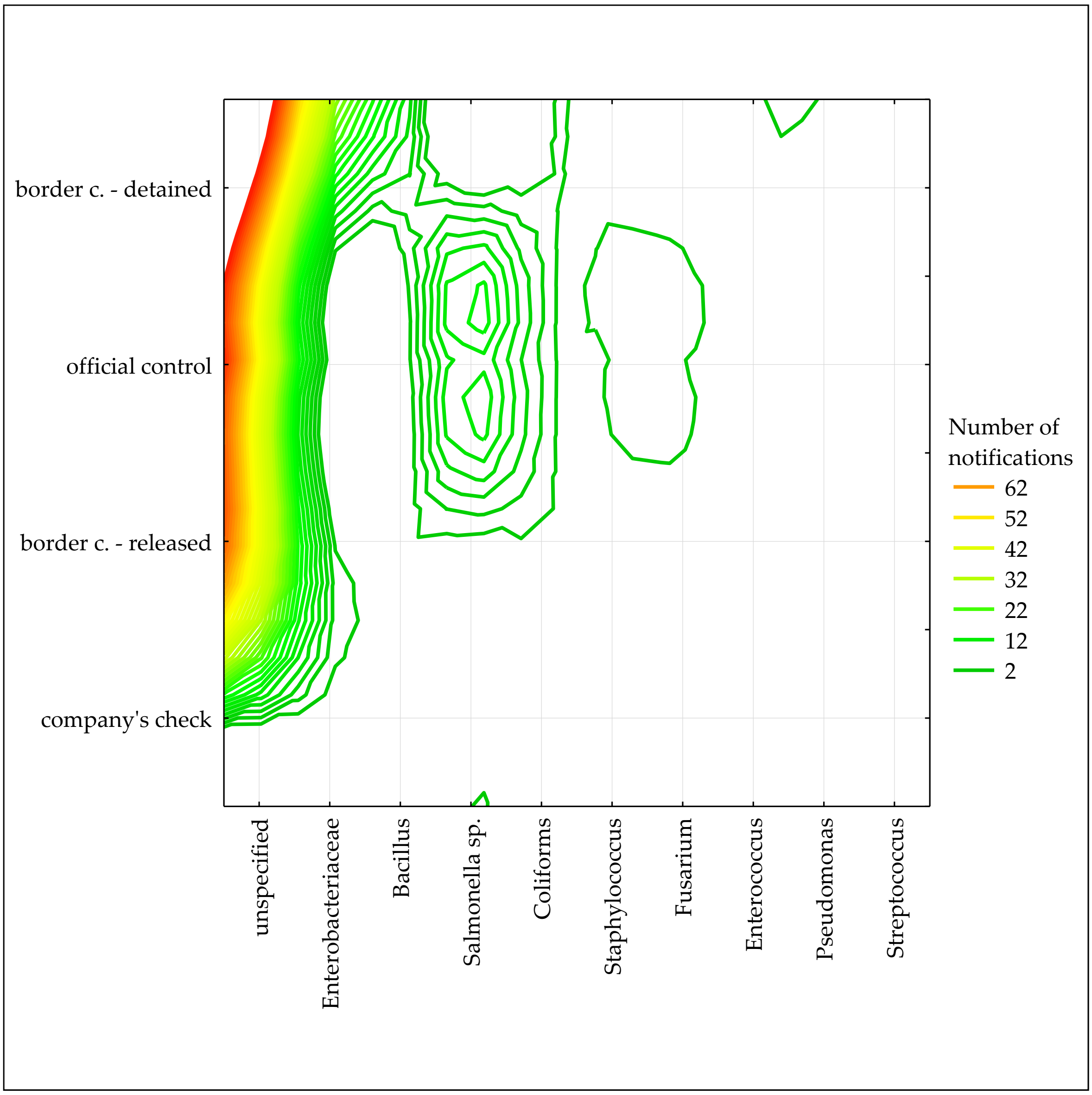

Figure S68. Similarities of RASFF notifications on non-pathogenic microorganisms and notification basis within feed using two-way joining.

border c. - detained – border control - consignment detained
border c. - released – border control - consignment released
company's check – company's own check
official control – official control on the market

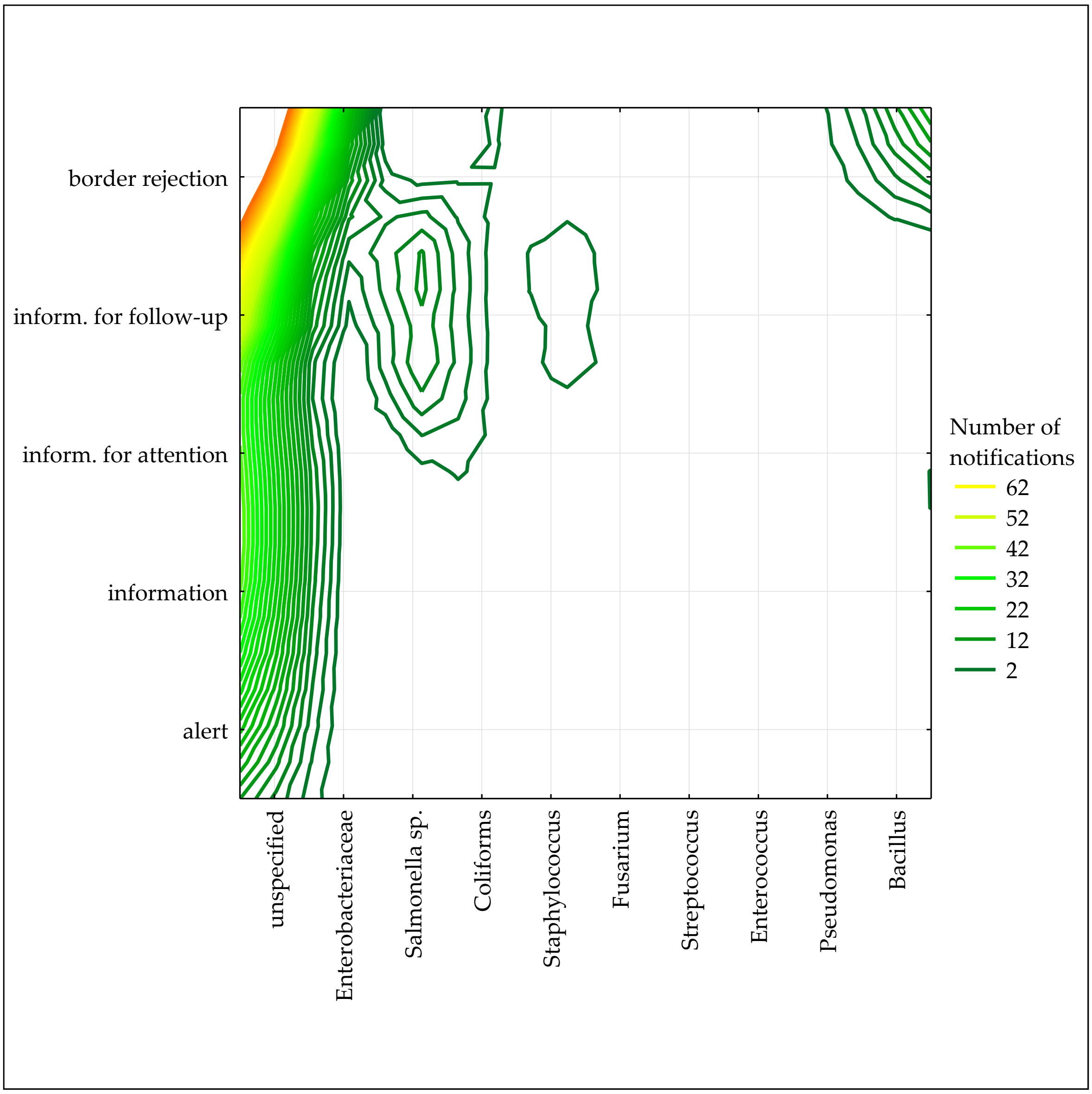

Figure S69. Similarities of RASFF notifications on non-pathogenic microorganisms and notification type within feed using two-way joining.

inform. for attention – information for attention
inform. for follow-up – information for follow-up

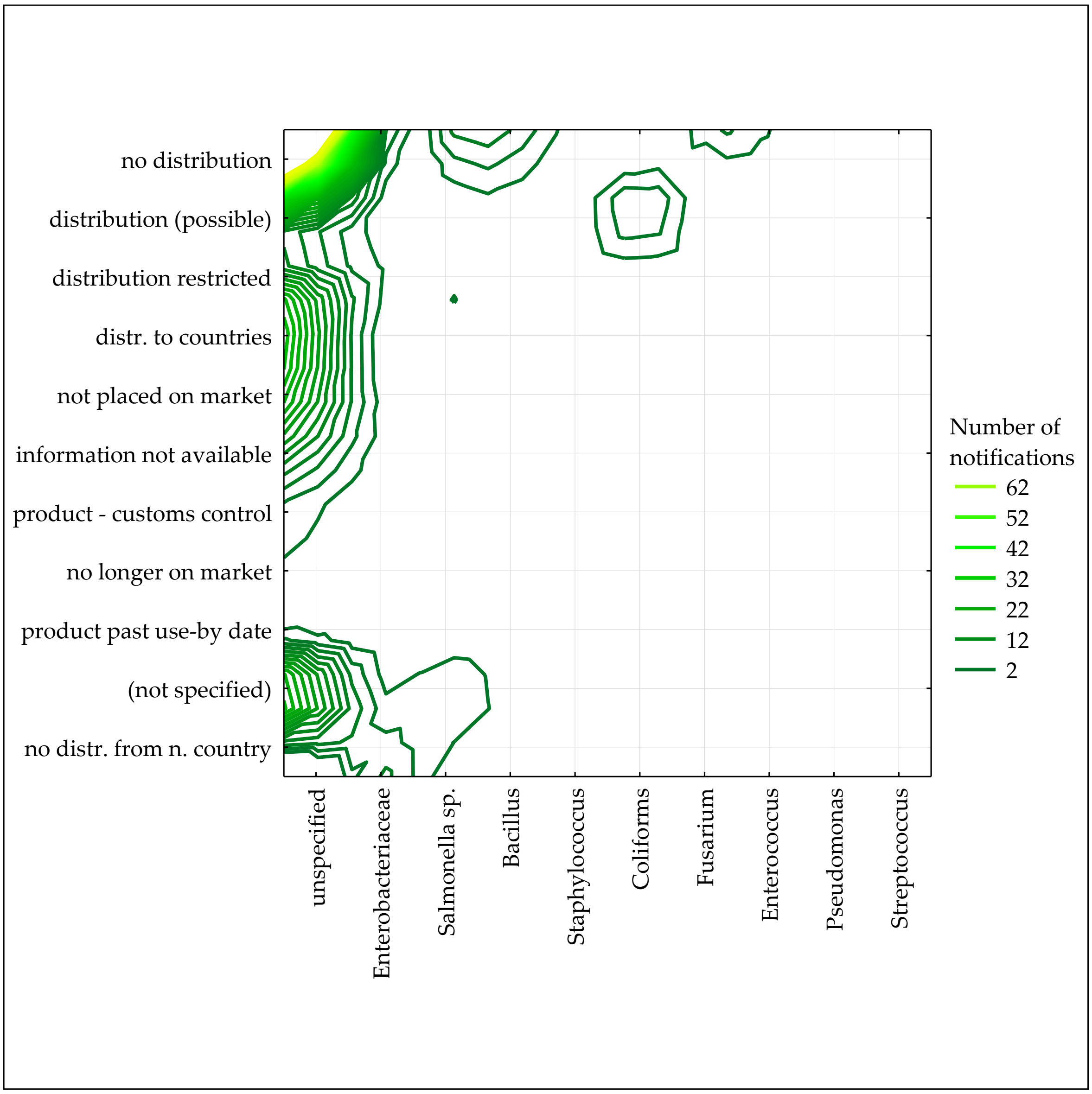

Figure S70. Similarities of RASFF notifications on non-pathogenic microorganisms and distribution status within feed using two-way joining.

distr. to countries – distribution to other member countries
distribution (possible) – distribution on the market (possible)
distribution restricted – distribution restricted to notifying country
information not available – information on distribution not (yet) available
no distr. from n. country – no distribution from notifying country
no longer on market – product (presumably) no longer on the market
not placed on market – product not (yet) placed on the market
product - customs control – product under customs control

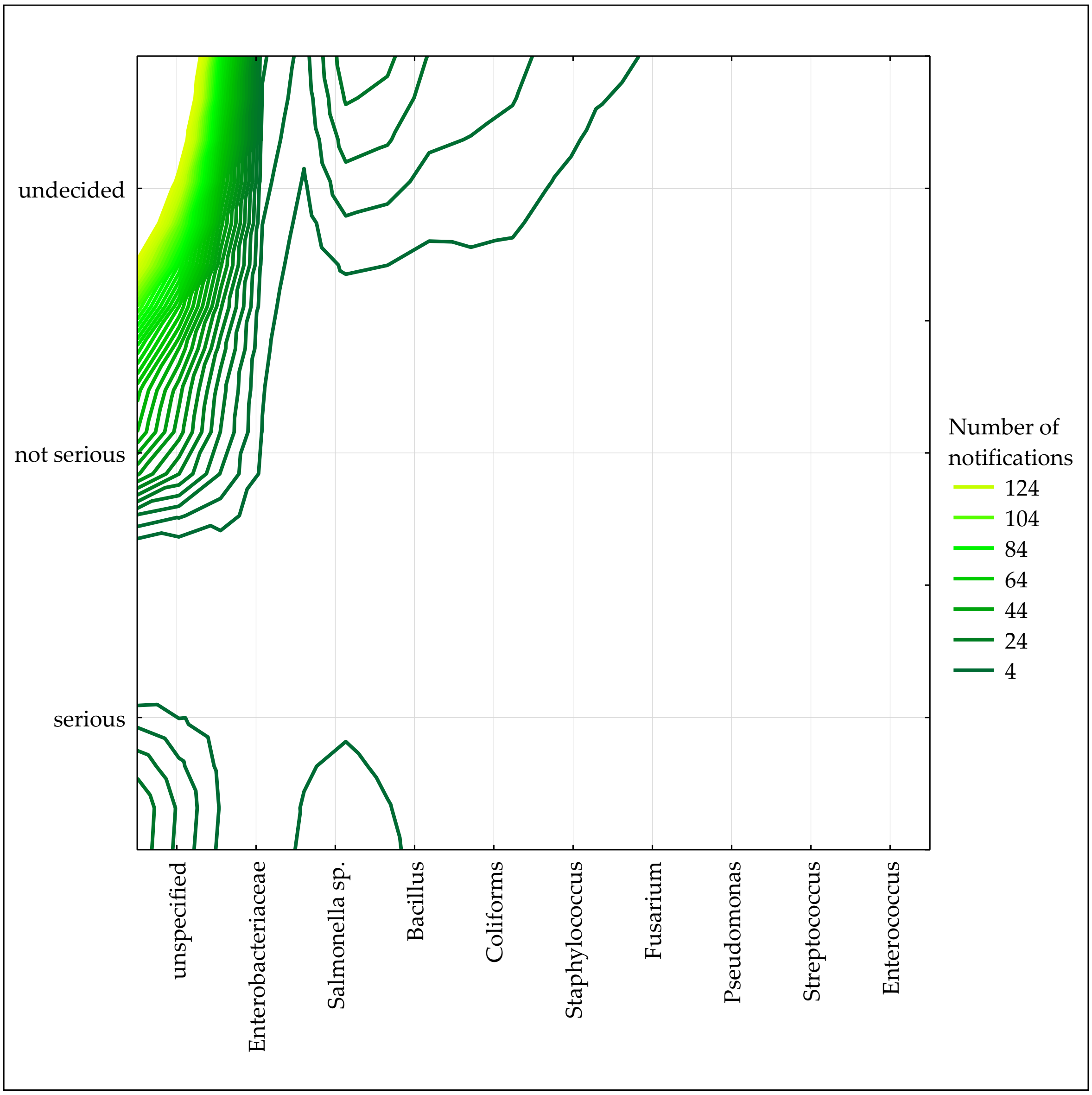

Figure S71. Similarities of RASFF notifications on non-pathogenic microorganisms and risk decision within feed using two-way joining.

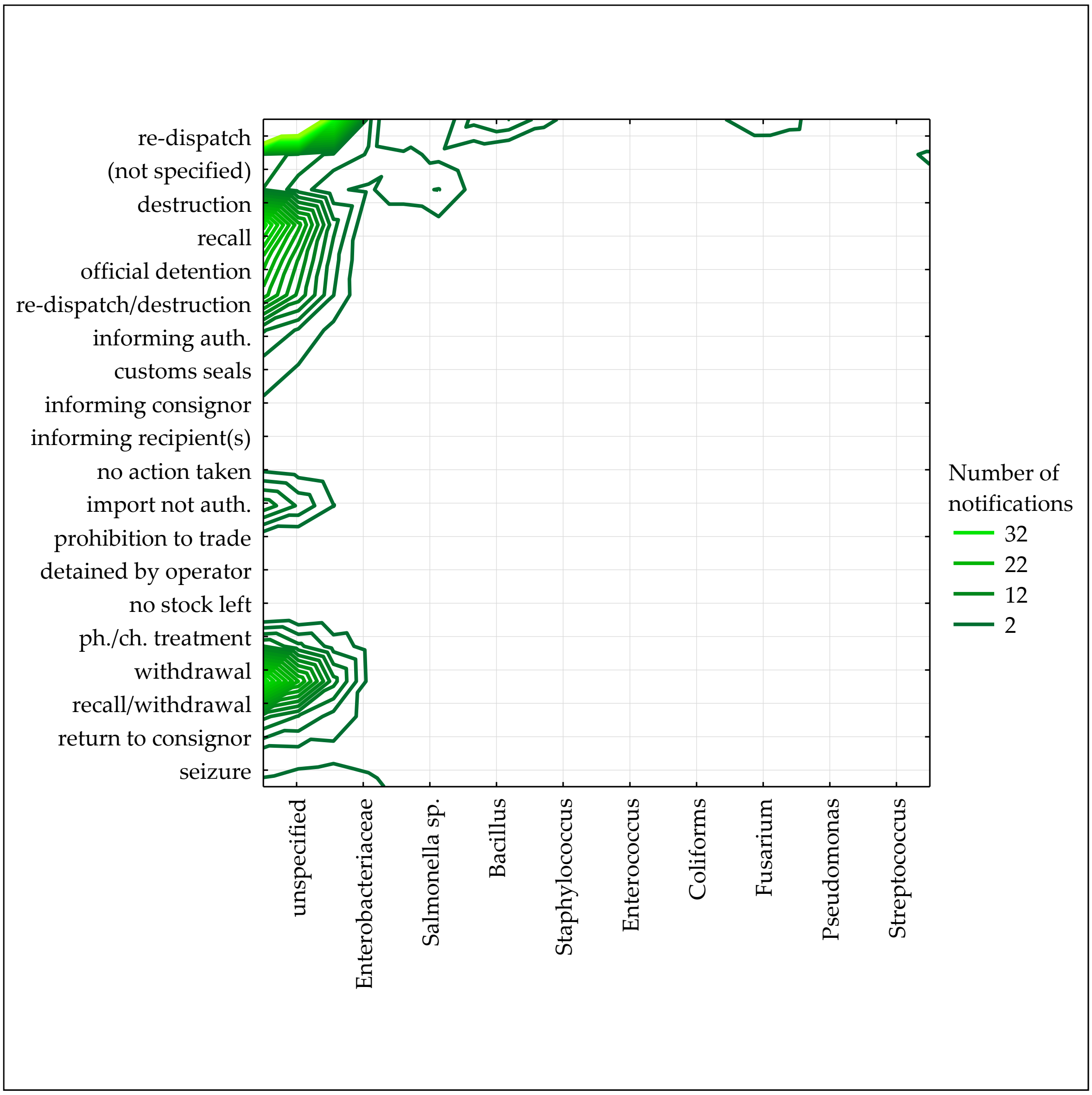

Figure S72. Similarities of RASFF notifications on non-pathogenic microorganisms and action taken within feed using two-way joining.

customs seals – placed under customs seals
import not auth. – import not authorised
informing auth. – informing authorities
ph./ch. treatment – physical/chemical treatment
prohibition to trade – prohibition to trade - sales ban
recall – recall from consumers
recall/withdrawal – product recall or withdrawal
re-dispatch/destruction – re-dispatch or destruction
withdrawal – withdrawal from the market
